# Supplementary material for: Prediction of glycopeptide fragment mass spectra by deep learning
Source: Nat Commun. 2024 Mar 19;15:2448. doi: 10.1038/s41467-024-46771-1 (PMC10951270; doi:10.1038/s41467-024-46771-1)
Supplement: Supplementary file 1 — Supplementary information [file 41467_2024_46771_MOESM1_ESM.pdf]

Supplementary Information for

Prediction of glycopeptide fragment mass spectra by deep learning

Yi Yang et al.

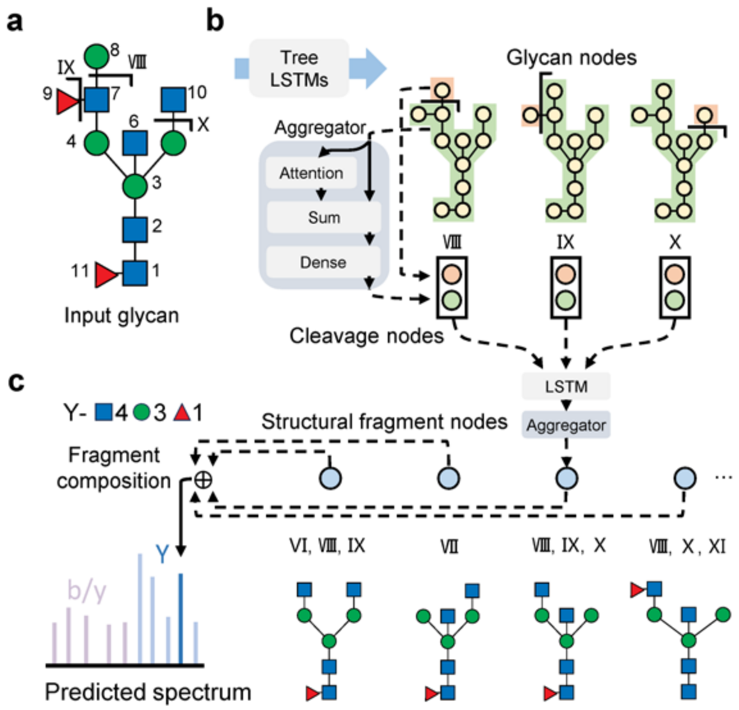

Supplementary Fig. 1. Illustration of the glycan fragmentation graph for prediction of Y ions.

(**a**) The glycan tree of an input glycopeptide, where monosaccharides are numbered in Arabic numerals and the cleavage site at the non-reducing end of each monosaccharide is represented in Roman numerals. (**b**) Monosaccharide nodes, with features generated by the tree LSTM networks, are split into lost nodes and those retained in the Y fragment for each potential cleavage. Features of the lost nodes and retained nodes are combined by an aggregator with attention mechanism, respectively, generating the feature of the cleavage. Cleavage nodes are then aggregated to structure-specific fragment nodes through an LSTM followed by an aggregator. (**c**) Relative intensities of Y ions are predicted, where isomeric fragments with the same monosaccharide composition are summed up.

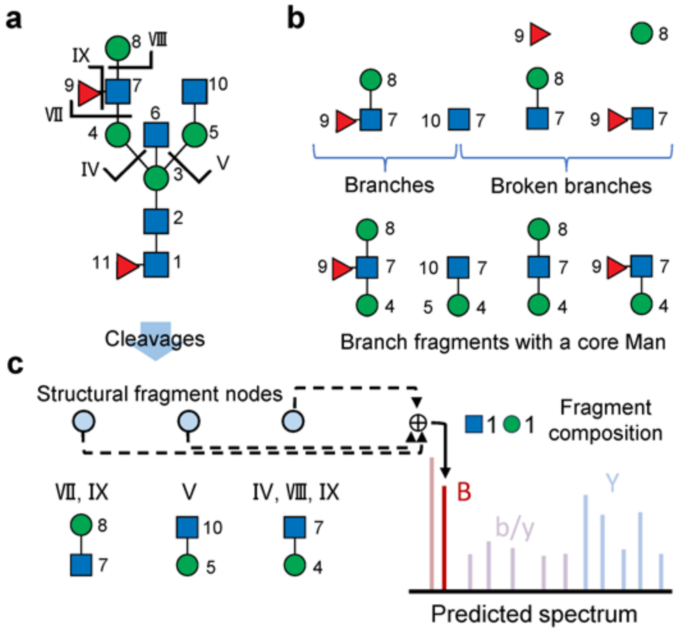

Supplementary Fig. 2. Illustration of the glycan fragmentation graph for prediction of B ions.

(**a**) The glycan tree of an input glycopeptide, where monosaccharides are numbered in Arabic numerals and the cleavage site at the non-reducing end of each monosaccharide is represented in Roman numerals. (**b**) The model considers fragment ions from the glycan branches, including the whole branch falling off the glycan core, fragments originated from cleavages within the branch, as well as the branch and its fragments with the adjacent core mannose. (**c**) Relative intensities of B ions are predicted by feature aggregation from cleavage nodes to structure-specific fragment nodes, where isomeric fragments with the same monosaccharide composition are finally summed up.

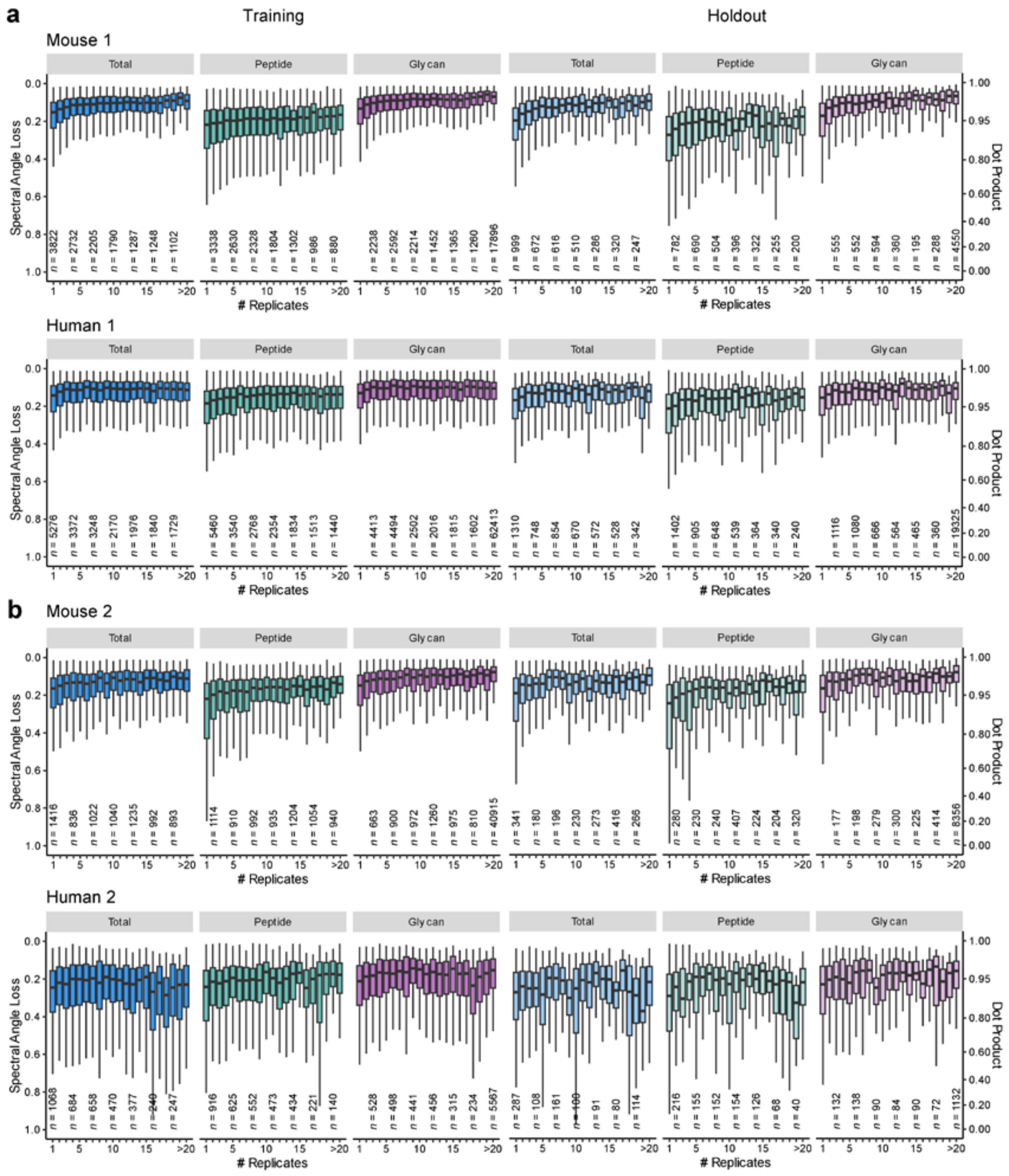

Supplementary Fig. 3. Performance of glycopeptide fragment spectrum prediction evaluated on all replicate spectra.

(**a**) Distributions of spectral similarities between predicted and experimental fragment ion intensities for glycopeptides contained in the training or holdout set of Mouse 1 and Human 1. (**b**) Distributions of spectral similarities for Mouse 2 and Human 2 after model finetuning. Related to **Fig. 2a** and **2c**. The center lines indicate the median values. The lower/upper hinges of the boxes indicate the first/third quartiles, and the lower/upper whiskers extend from the hinges to the smallest/largest value no further than 1.5 times the interquartile range. The data are grouped by the number of replicate spectra of each glycopeptide precursor, and the number of spectra (*n*) in each group is indicated. Source data are provided as a Source Data file.

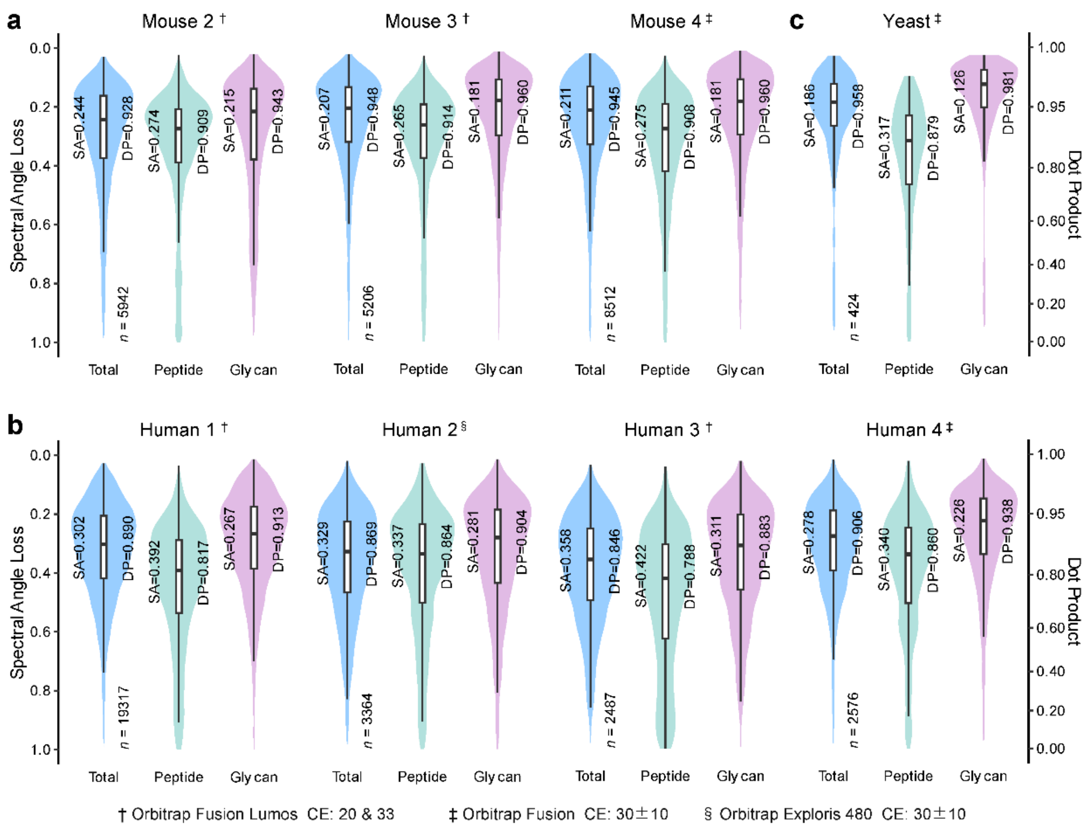

Supplementary Fig. 4. Performance of glycopeptide fragment spectrum prediction using a model trained with Mouse 1 evaluated on consensus spectra.

(**a**) Distributions of spectral similarities between predicted and experimental fragment ion intensities, tested on other mouse datasets. (**b**) Results tested on human datasets. (**c**) Results tested on the yeast dataset. Instrument settings of each dataset are marked. Spectral similarities are computed for peptide b/y ions and glycan Y ions separately, as well as for the total spectrum of peptide and glycan ions. The median values of spectral angle loss (SA) and dot product (DP), as well as data size (*n*), are indicated. The lower/upper hinges of the boxes indicate the first/third quartiles, and the lower/upper whiskers extend from the hinges to the smallest/largest value no further than 1.5 times the interquartile range. Source data are provided as a Source Data file.

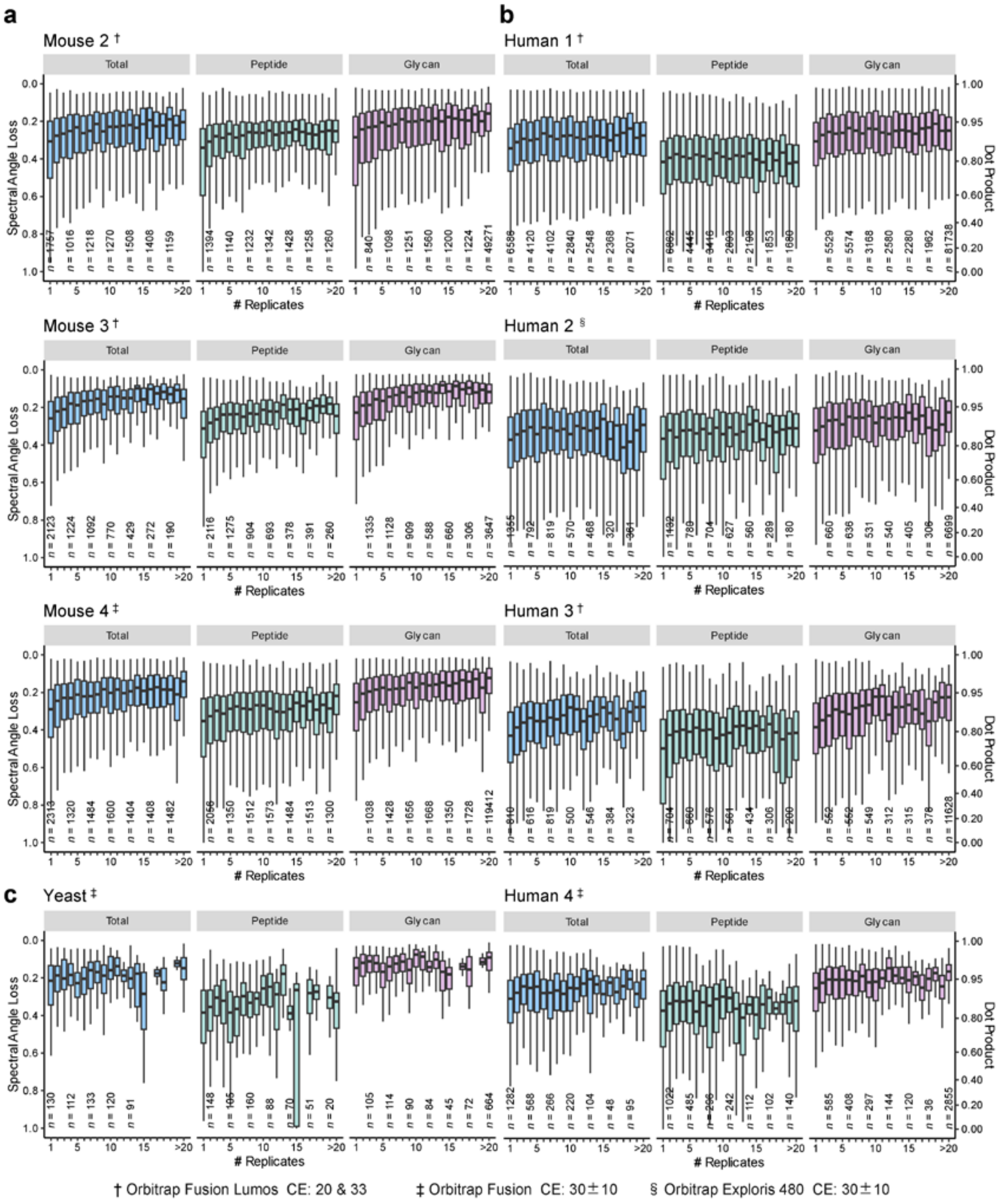

Supplementary Fig. 5. Performance of glycopeptide fragment spectrum prediction using the model trained with Mouse 1 evaluated on all replicate spectra.

(**a**) Distributions of spectral similarities between predicted and experimental fragment ion intensities, tested on other mouse datasets. (**b**) Results tested on human datasets. (**c**) Results tested on the yeast dataset. Related to **Supplementary Fig. 4**. The center lines indicate the median values. The lower/upper hinges of the boxes indicate the first/third quartiles, and the lower/upper whiskers extend from the hinges to the smallest/largest value no further than 1.5 times the interquartile range. The data are grouped by the number of replicate spectra of each glycopeptide precursor, and the number of spectra (n) in each group is indicated. Source data are provided as a Source Data file.

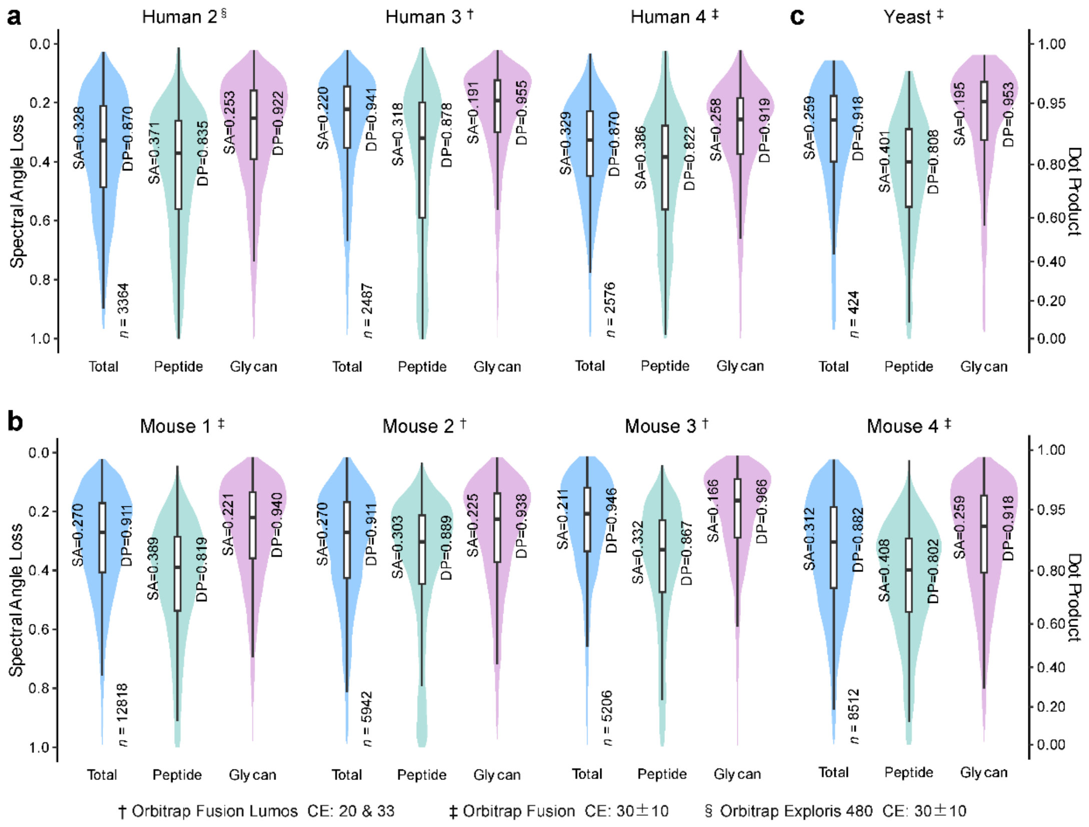

Supplementary Fig. 6. Performance of glycopeptide fragment spectrum prediction using a model trained with Human 1 evaluated on consensus spectra.

(**a**) Distributions of spectral similarities between predicted and experimental fragment ion intensities, tested on other human datasets. (**b**) Results tested on mouse datasets. (**c**) Results tested on the yeast dataset. Instrument settings of each dataset are marked. Spectral similarities are computed for peptide b/y ions and glycan Y ions separately, as well as for the total spectrum of peptide and glycan ions. The median values of spectral angle loss (SA) and dot product (DP), as well as data size (*n*), are indicated. The lower/upper hinges of the boxes indicate the first/third quartiles, and the lower/upper whiskers extend from the hinges to the smallest/largest value no further than 1.5 times the interquartile range. Source data are provided as a Source Data file.

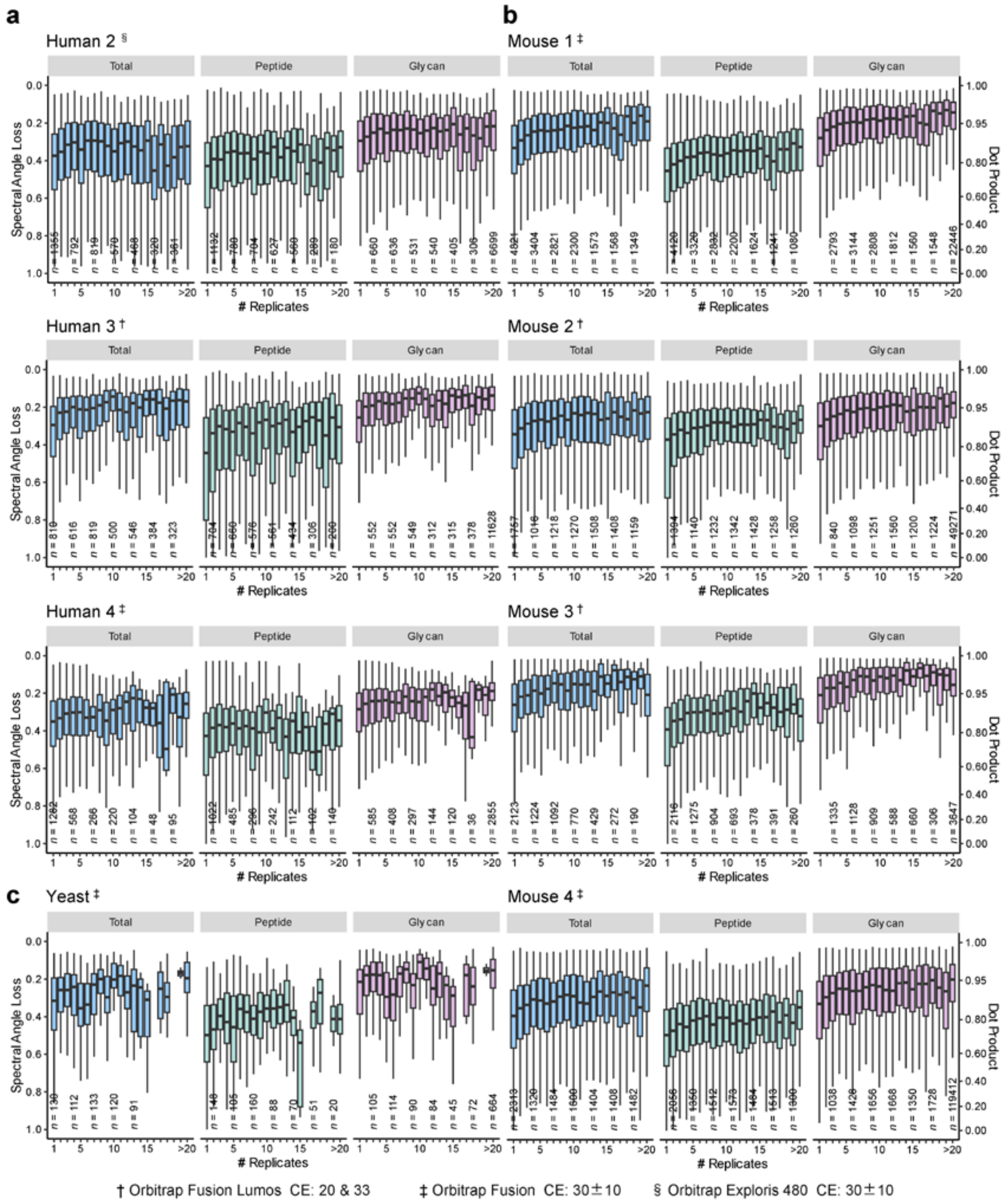

Supplementary Fig. 7. Performance of glycopeptide fragment spectrum prediction using the model trained with Human 1 evaluated on all replicate spectra.

(**a**) Distributions of spectral similarities between predicted and experimental fragment ion intensities, tested on other mouse datasets. (**b**) Results tested on mouse datasets. (**c**) Results tested on the yeast dataset. Related to **Supplementary Fig. 6**. The center lines indicate the median values. The lower/upper hinges of the boxes indicate the first/third quartiles, and the lower/upper whiskers extend from the hinges to the smallest/largest value no further than 1.5 times the interquartile range. The data are grouped by the number of replicate spectra of each glycopeptide precursor, and the number of spectra (n) in each group is indicated. Source data are provided as a Source Data file.

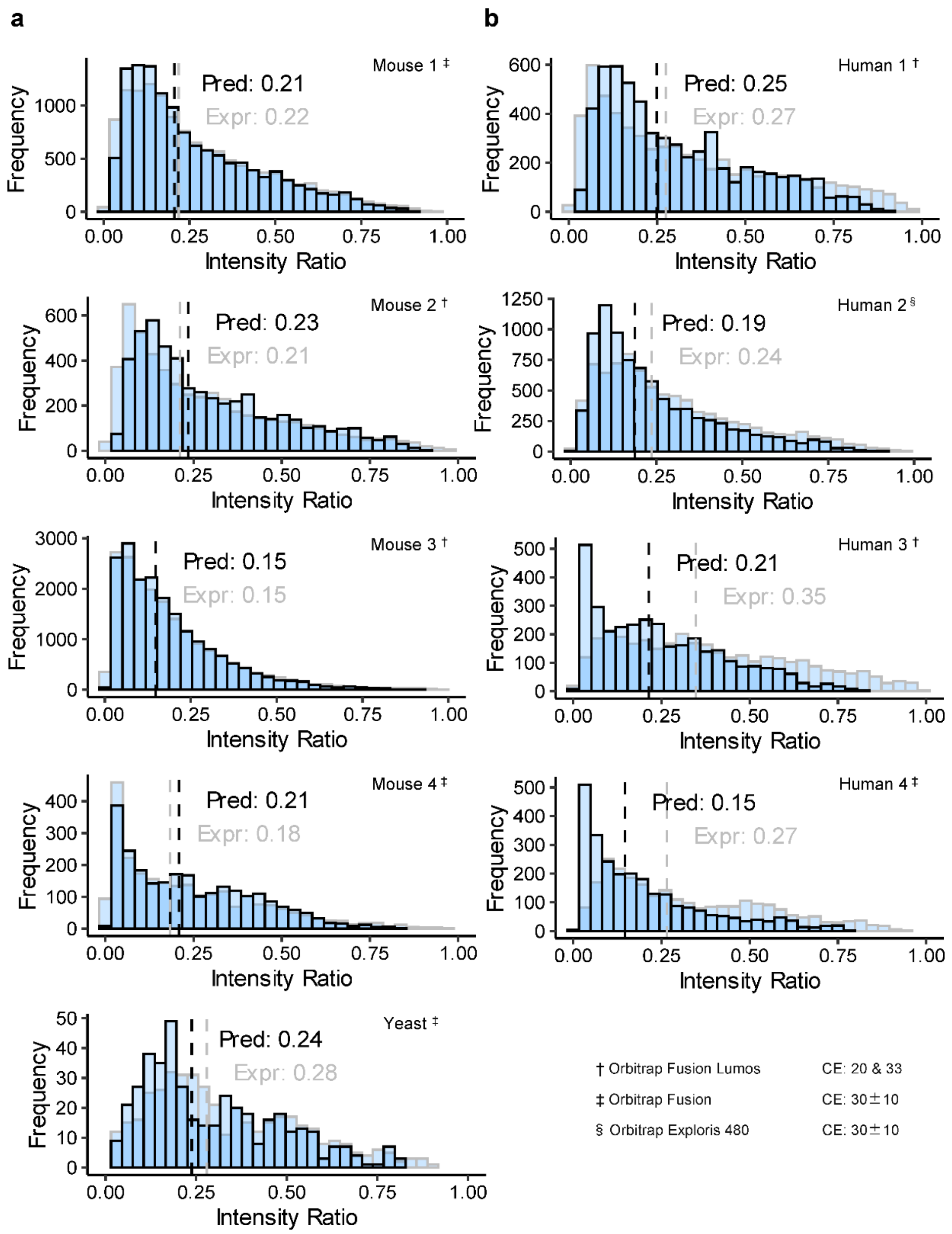

Supplementary Fig. 8. Intensity ratio between peptide and glycan fragments calculated on consensus spectra.

(**a**) Distributions of intensity ratio values in the mouse and yeast datasets. Spectra prediction was performed using the model trained with Mouse 1. (**b**) Distributions for the human datasets. Spectra prediction was performed using the model trained with Human 1. The median values of the predicted (Pred) and experimental (Expr) intensity ratio are indicated. Source data are provided as a Source Data file.

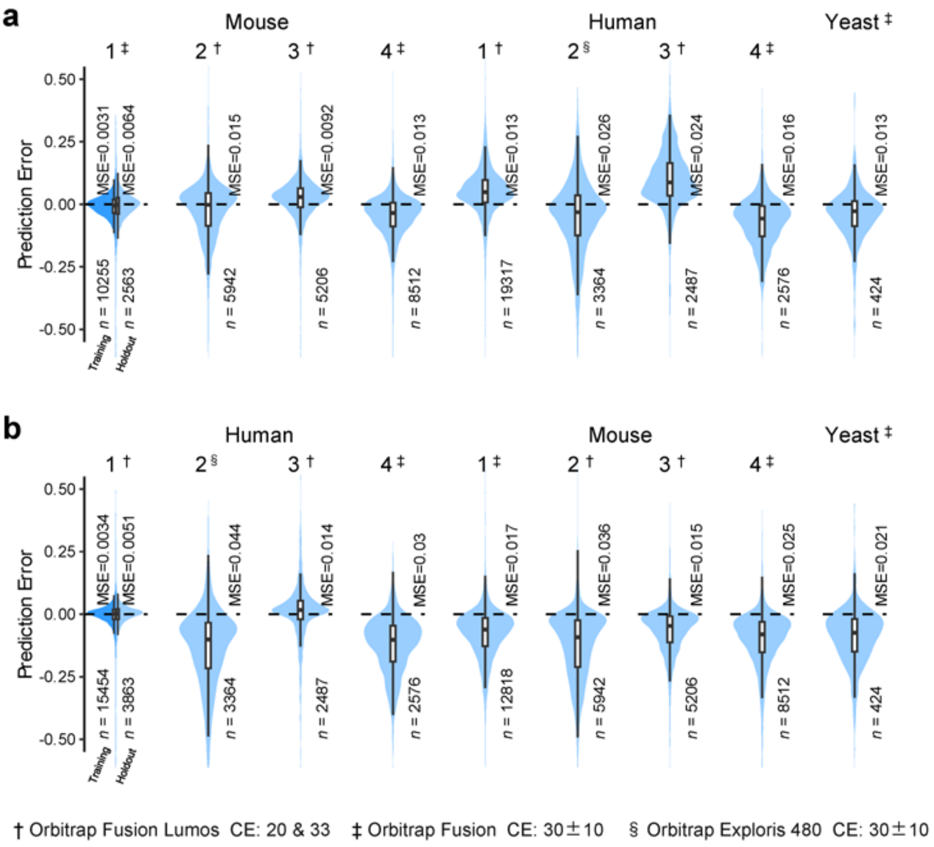

Supplementary Fig. 9. Prediction error of intensity ratio between peptide and glycan fragments evaluated on consensus spectra.

(**a**) Distributions of prediction error between predicted and experimental intensity ratio values using the model trained with Mouse 1. (**b**) Results predicted using the model trained with Human 1. The mean squared error (MSE) and data size (*n*) are indicated. The lower/upper hinges of the boxes indicate the first/third quartiles, and the lower/upper whiskers extend from the hinges to the smallest/largest value no further than 1.5 times the interquartile range. Source data are provided as a Source Data file.

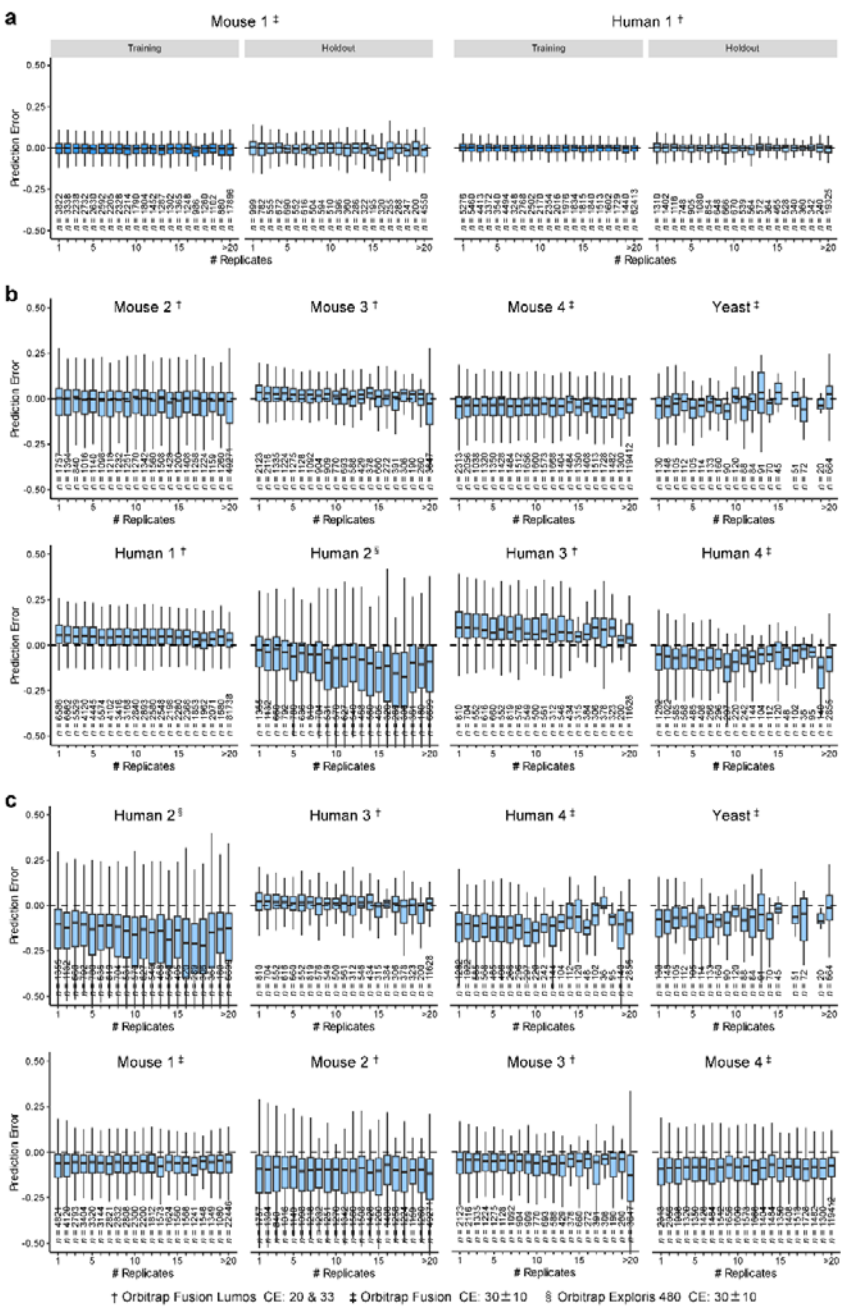

Supplementary Fig. 10. Prediction error of intensity ratio between peptide and glycan fragments evaluated on all replicate spectra.

(**a**) Distributions of prediction error between predicted and experimental intensity ratio values for glycopeptides contained in the training or holdout set of Mouse 1 and Human 1. (**b**) Results predicted using the model trained with Mouse 1. (**c**) Results predicted using the model trained with Human 1. Related to **Supplementary Fig. 9**. The center lines indicate the median values. The lower/upper hinges of the boxes indicate the first/third quartiles, and the lower/upper whiskers extend from the hinges to the smallest/largest value no further than 1.5 times the interquartile range. The data are grouped by the number of replicate spectra of each glycopeptide precursor, and the number of spectra (*n*) in each group is indicated. Source data are provided as a Source Data file.

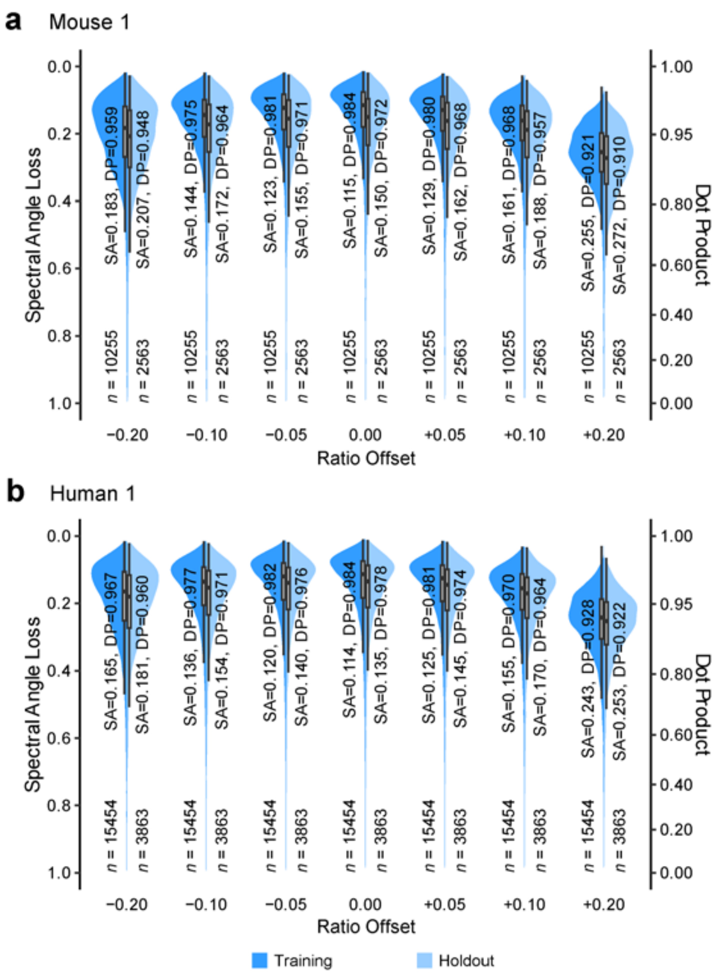

Supplementary Fig. 11. Impact of intensity ratio on performance of glycopeptide fragment spectrum prediction evaluated on consensus spectra.

(**a**) Distributions of spectral similarities between predicted and experimental fragment ion intensities when an offset is added to the predicted intensity ratio, tested on the Mouse 1 dataset. (**b**) Results tested on the Human 1 dataset. The median values of spectral angle loss (SA) and dot product (DP), as well as data size (*n*), are indicated. The lower/upper hinges of the boxes indicate the first/third quartiles, and the lower/upper whiskers extend from the hinges to the smallest/largest value no further than 1.5 times the interquartile range. Source data are provided as a Source Data file.

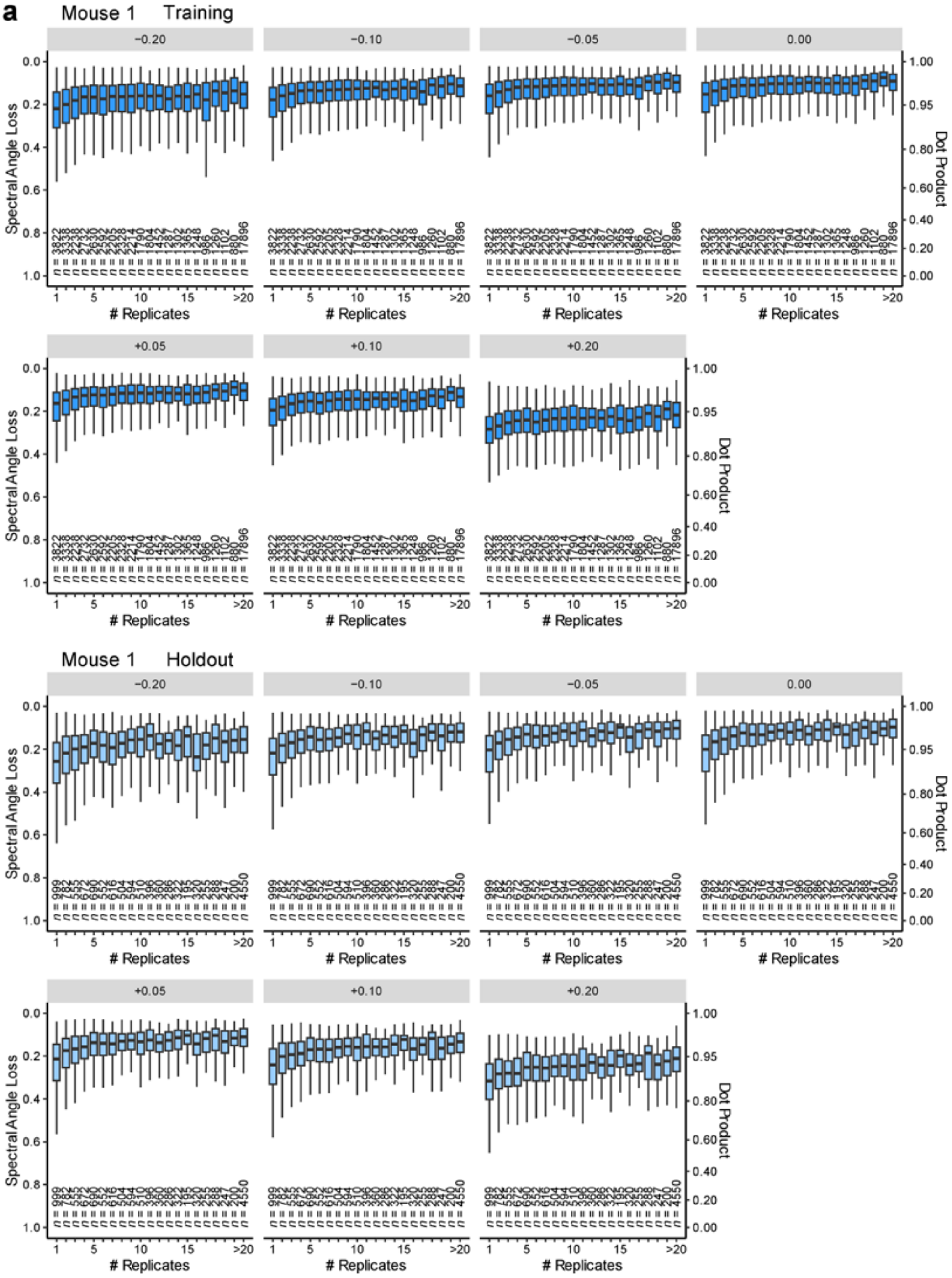

Supplementary Fig. 12. Impact of intensity ratio on performance of glycopeptide fragment spectrum prediction evaluated on all replicate spectra.

(**a**) Distributions of spectral similarities between predicted and experimental fragment ion intensities when an offset is added to the predicted intensity ratio, tested on the Mouse 1 dataset. Related to **Supplementary Fig. 11a**. (Continued on next page)

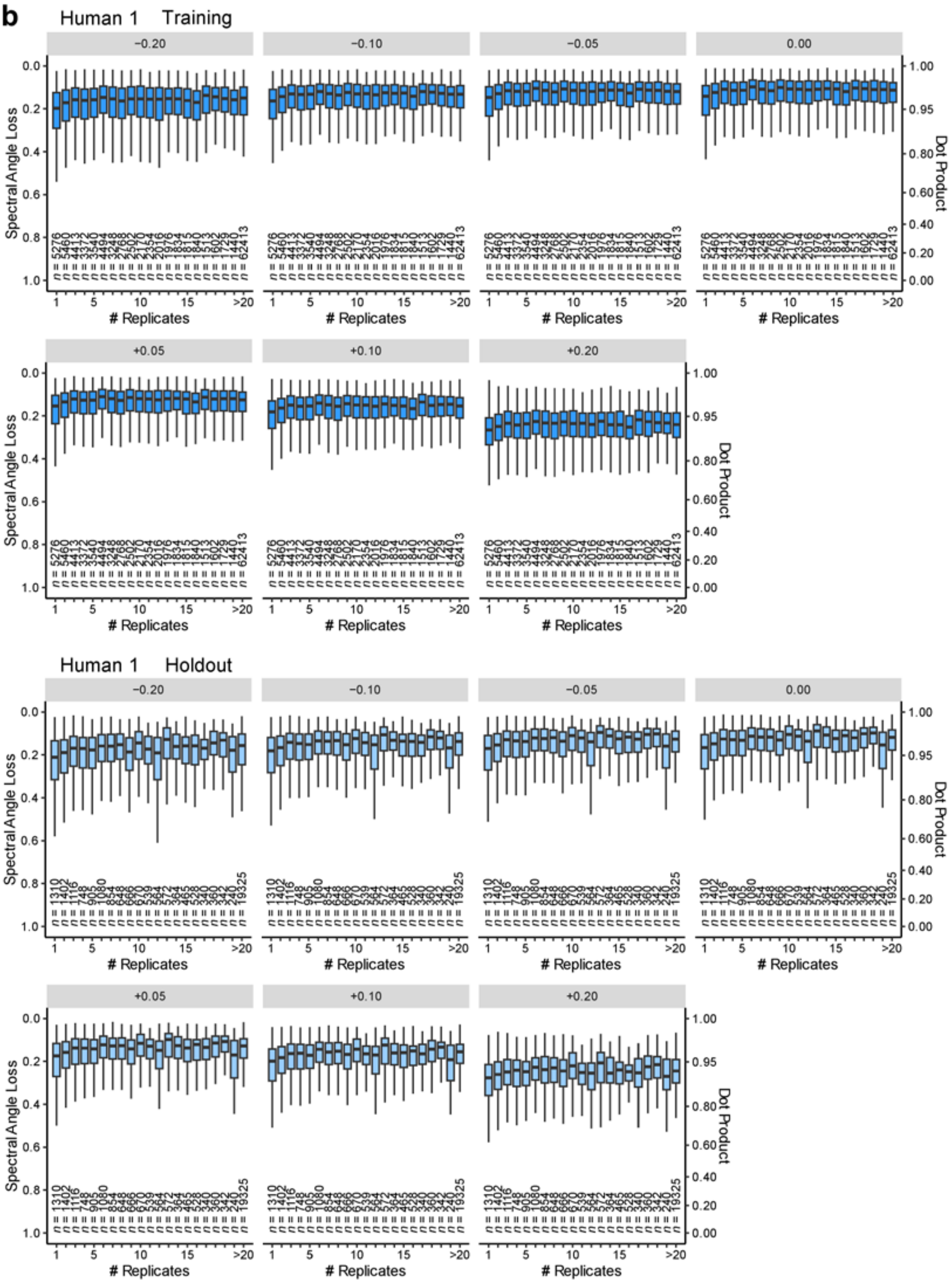

Supplementary Fig. 12 (Continued).

(**b**) Results tested on the Human 1 dataset. Related to **Supplementary Fig. 11b**. The center lines indicate the median values. The lower/upper hinges of the boxes indicate the first/third quartiles, and the lower/upper whiskers extend from the hinges to the smallest/largest value no further than 1.5 times the interquartile range. The data are grouped by the number of replicate spectra of each glycopeptide precursor, and the number of spectra (*n*) in each group is indicated. Source data are provided as a Source Data file.

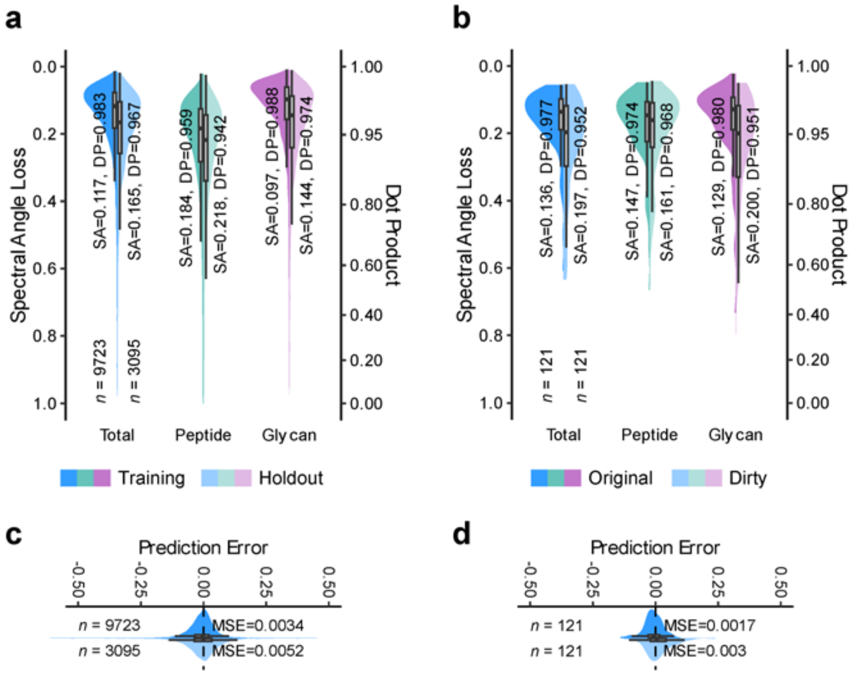

Supplementary Fig. 13. Impact of incorrectly identified spectra in the training data on performance of glycopeptide fragment spectrum prediction evaluated on consensus spectra.

(**a**) Distributions of spectral similarities between predicted and experimental fragment ion intensities, where spectra are predicted using a model trained with a dirty dataset (containing intentional incorrect GPSMs) and compared to experimental spectra in the Mouse 1 dataset. (**b**) Performance comparison between the models trained with the original Mouse 1 dataset and the dirty dataset, evaluated on the glycopeptides related to the incorrect GPSMs. (**c**) Distributions of prediction error of the intensity ratio using the dirty model, evaluated on the Mouse1 dataset. (**d**) Prediction error of the intensity ratio evaluated on the glycopeptides related to the incorrect GPSMs. The median values of spectral angle loss (SA) and dot product (DP), as well as data size (*n*) and mean squared error (MSE), are indicated. The lower/upper hinges of the boxes indicate the first/third quartiles, and the lower/upper whiskers extend from the hinges to the smallest/largest value no further than 1.5 times the interquartile range. Source data are provided as a Source Data file.

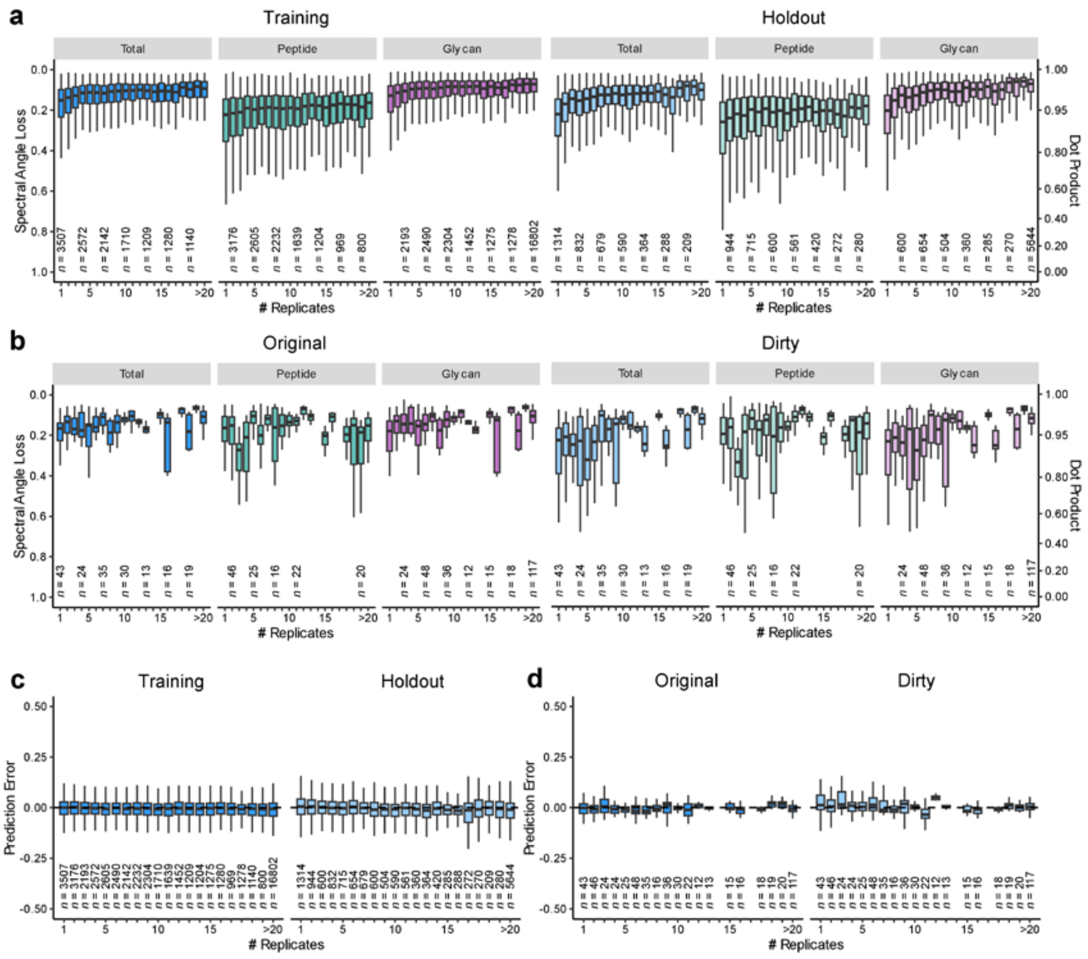

Supplementary Fig. 14. Impact of incorrectly identified spectra in the training data on performance of glycopeptide fragment spectrum prediction evaluated on all replicate spectra.

(**a**) Distributions of spectral similarities between predicted and experimental fragment ion intensities, where spectra are predicted using a model trained with a dirty dataset (containing intentional incorrect GPSMs) and compared to experimental spectra in the Mouse 1 dataset. (**b**) Performance comparison between the models trained with the original Mouse 1 dataset and the dirty dataset, evaluated on the glycopeptides related to the incorrect GPSMs. (**c**) Distributions of prediction error of the intensity ratio using the dirty model, evaluated on the Mouse1 dataset. (**d**) Prediction error of the intensity ratio evaluated on the glycopeptides related to the incorrect GPSMs. Related to **Supplementary Fig. 13**. The center lines indicate the median values. The lower/upper hinges of the boxes indicate the first/third quartiles, and the lower/upper whiskers extend from the hinges to the smallest/largest value no further than 1.5 times the interquartile range. The data are grouped by the number of replicate spectra of each glycopeptide precursor, and the number of spectra (*n*) in each group is indicated. Source data are provided as a Source Data file.

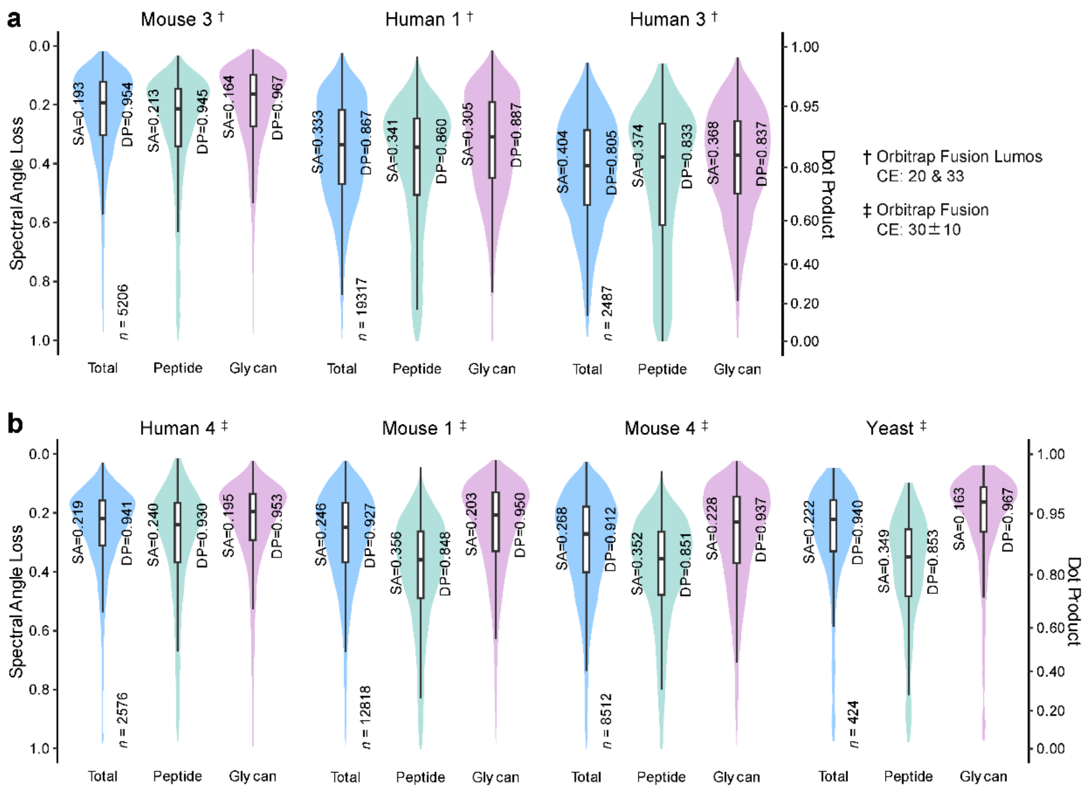

Supplementary Fig. 15. Performance of glycopeptide fragment spectrum prediction using finetuned models evaluated on consensus spectra.

(**a**) Distributions of spectral similarities between predicted and experimental fragment ion intensities, where spectra are predicted using the model trained with Mouse 1 and finetuned with Mouse 2. (**b**) Results using the model trained with Human 1 and finetuned with Human 2. Instrument settings of each dataset are marked. Spectral similarities are computed for peptide b/y ions and glycan Y ions separately, as well as for the total spectrum of peptide and glycan ions. The median values of spectral angle loss (SA) and dot product (DP), as well as data size (*n*), are indicated. The lower/upper hinges of the boxes indicate the first/third quartiles, and the lower/upper whiskers extend from the hinges to the smallest/largest value no further than 1.5 times the interquartile range. Source data are provided as a Source Data file.

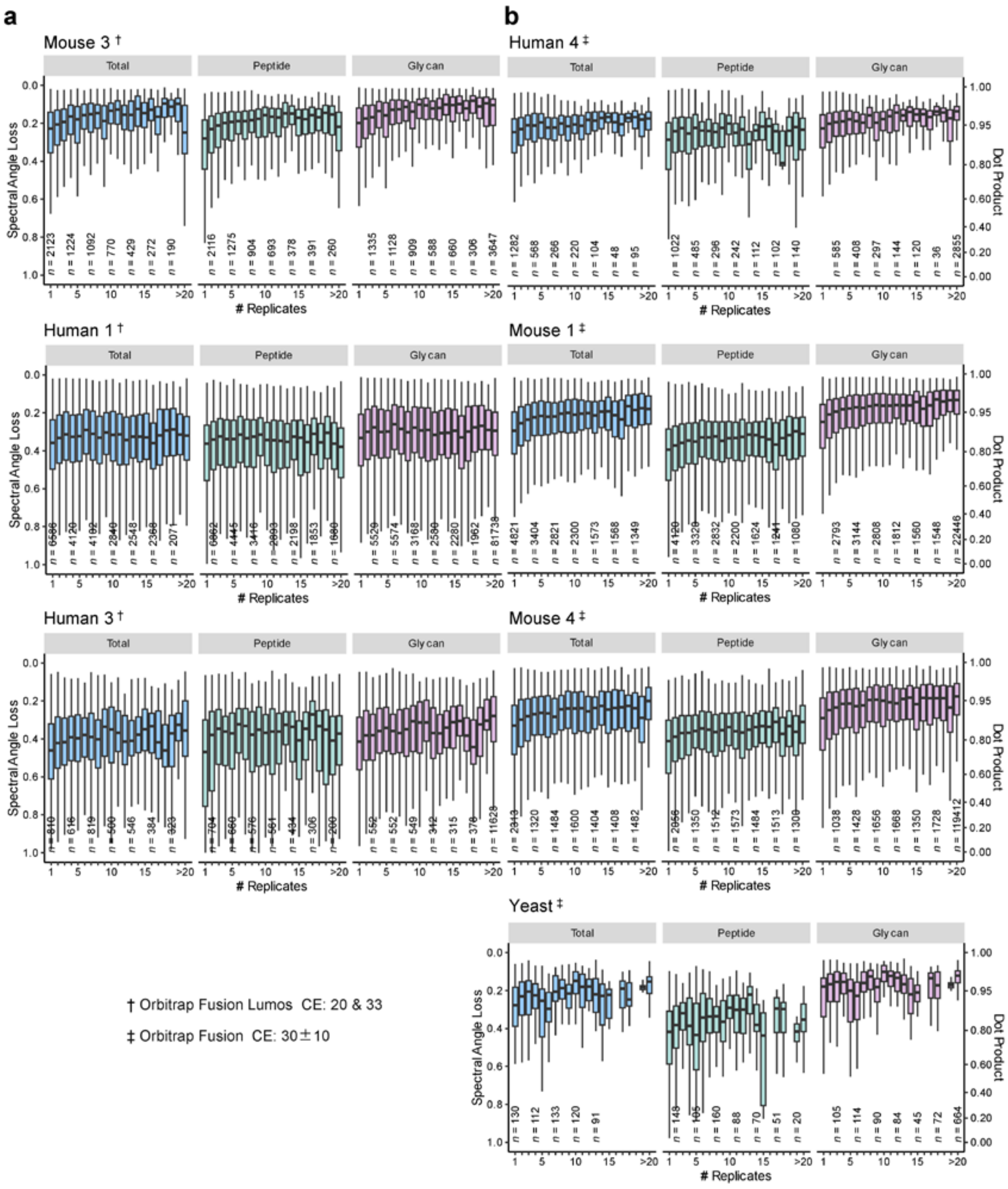

Supplementary Fig. 16. Performance of glycopeptide fragment spectrum prediction using finetuned models evaluated on all replicate spectra.

(**a**) Distributions of spectral similarities between predicted and experimental fragment ion intensities, where spectra are predicted using the model trained with Mouse 1 and finetuned with Mouse 2. (**b**) Results using the model trained with Human 1 and finetuned with Human 2. Related to **Supplementary Fig. 15**. The center lines indicate the median values. The lower/upper hinges of the boxes indicate the first/third quartiles, and the lower/upper whiskers extend from the hinges to the smallest/largest value no further than 1.5 times the interquartile range. The data are grouped by the number of replicate spectra of each glycopeptide precursor, and the number of spectra (*n*) in each group is indicated. Source data are provided as a Source Data file.

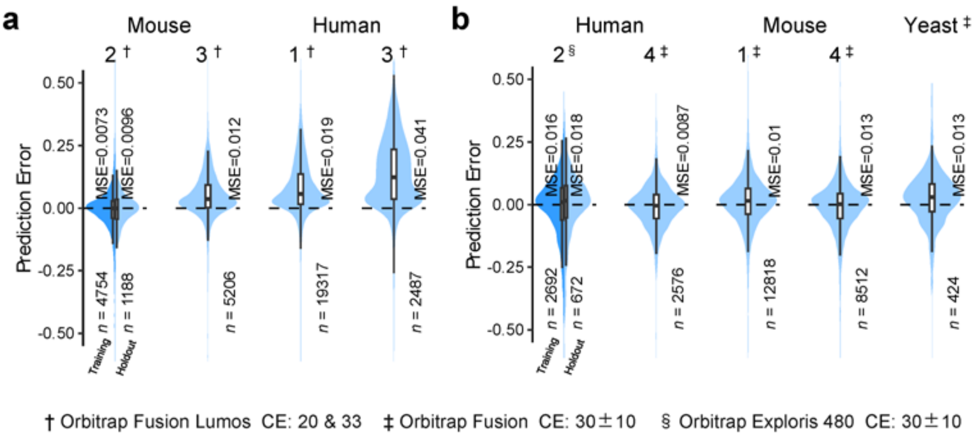

Supplementary Fig. 17. Prediction error of intensity ratio between peptide and glycan fragments using finetuned models evaluated on consensus spectra.

(**a**) Distributions of prediction error between predicted and experimental intensity ratio values, where spectra are predicted using the model trained with Mouse 1 and finetuned with Mouse 2. (**b**) Results predicted using the model trained with Human 1 and finetuned with Human 2. The mean squared error (MSE) and data size (*n*) are indicated. The lower/upper hinges of the boxes indicate the first/third quartiles, and the lower/upper whiskers extend from the hinges to the smallest/largest value no further than 1.5 times the interquartile range. Source data are provided as a Source Data file.

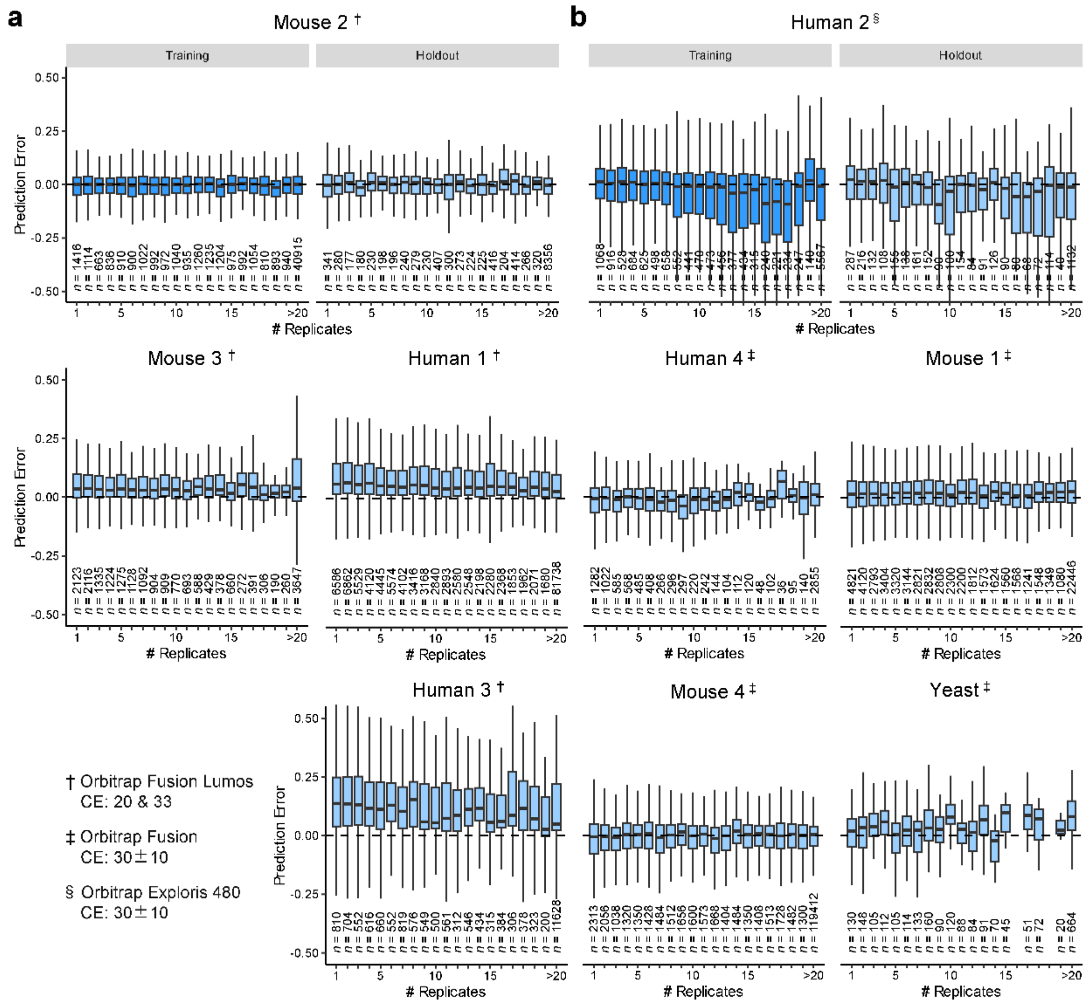

Supplementary Fig. 18. Prediction error of intensity ratio between peptide and glycan fragments using finetuned models evaluated on all replicate spectra.

(**a**) Distributions of prediction error between predicted and experimental intensity ratio values, where spectra are predicted using the model trained with Mouse 1 and finetuned with Mouse 2. (**b**) Results predicted using the model trained with Human 1 and finetuned with Human 2. Related to **Supplementary Fig. 17**. The center lines indicate the median values. The lower/upper hinges of the boxes indicate the first/third quartiles, and the lower/upper whiskers extend from the hinges to the smallest/largest value no further than 1.5 times the interquartile range. The data are grouped by the number of replicate spectra of each glycopeptide precursor, and the number of spectra (*n*) in each group is indicated. Source data are provided as a Source Data file.

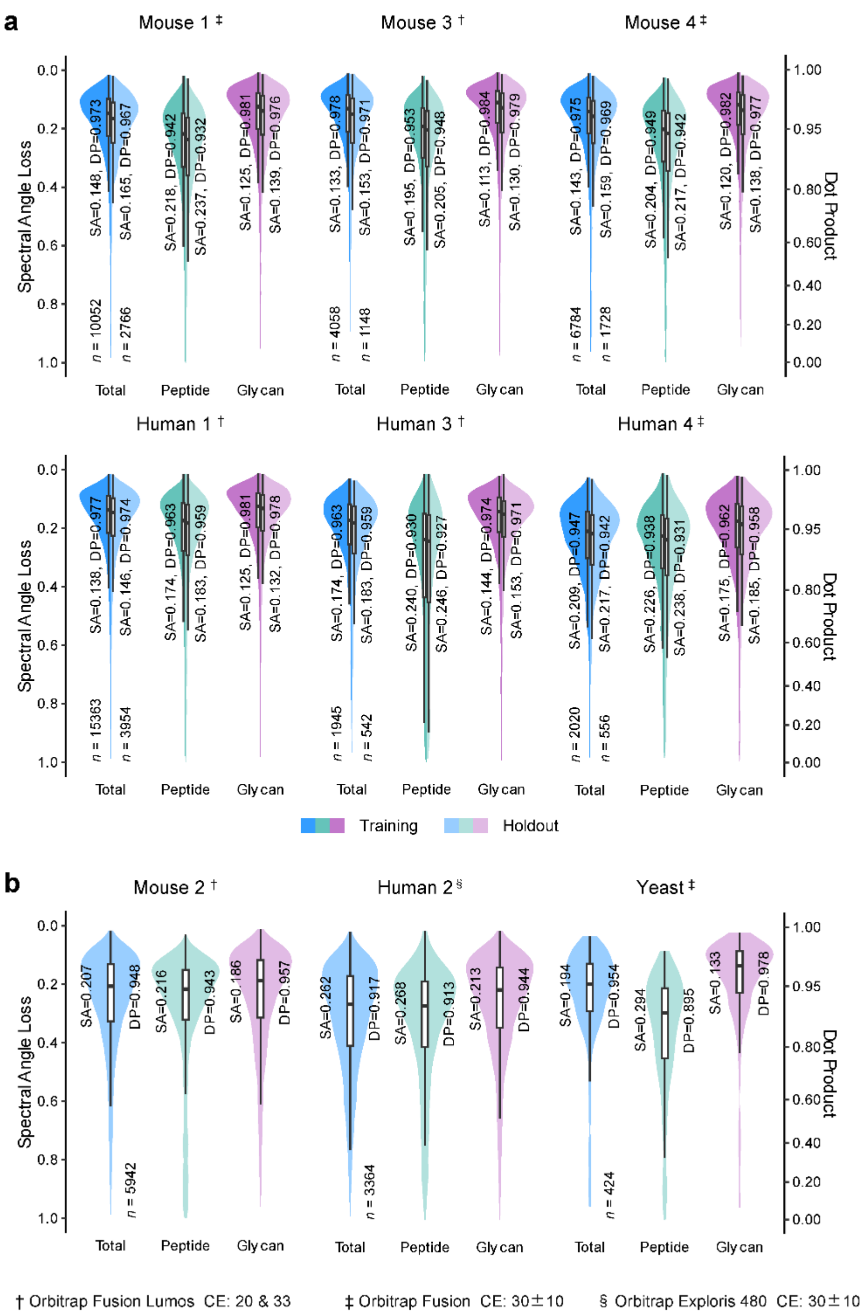

Supplementary Fig. 19. Performance of glycopeptide fragment spectrum prediction using a model trained with a combined dataset evaluated on consensus spectra.

(**a**) Distributions of spectral similarities between predicted and experimental fragment ion intensities for glycopeptides contained in the six datasets that have been merged into the combined dataset. (**b**) Results tested on the other three datasets. Instrument settings of each dataset are marked. Spectral similarities are computed for peptide b/y ions and glycan Y ions separately, as well as for the total spectrum of peptide and glycan ions. The median values of spectral angle loss (SA) and dot product (DP), as well as data size (*n*), are indicated. The lower/upper hinges of the boxes indicate the first/third quartiles, and the lower/upper whiskers extend from the hinges to the smallest/largest value no further than 1.5 times the interquartile range. Source data are provided as a Source Data file.

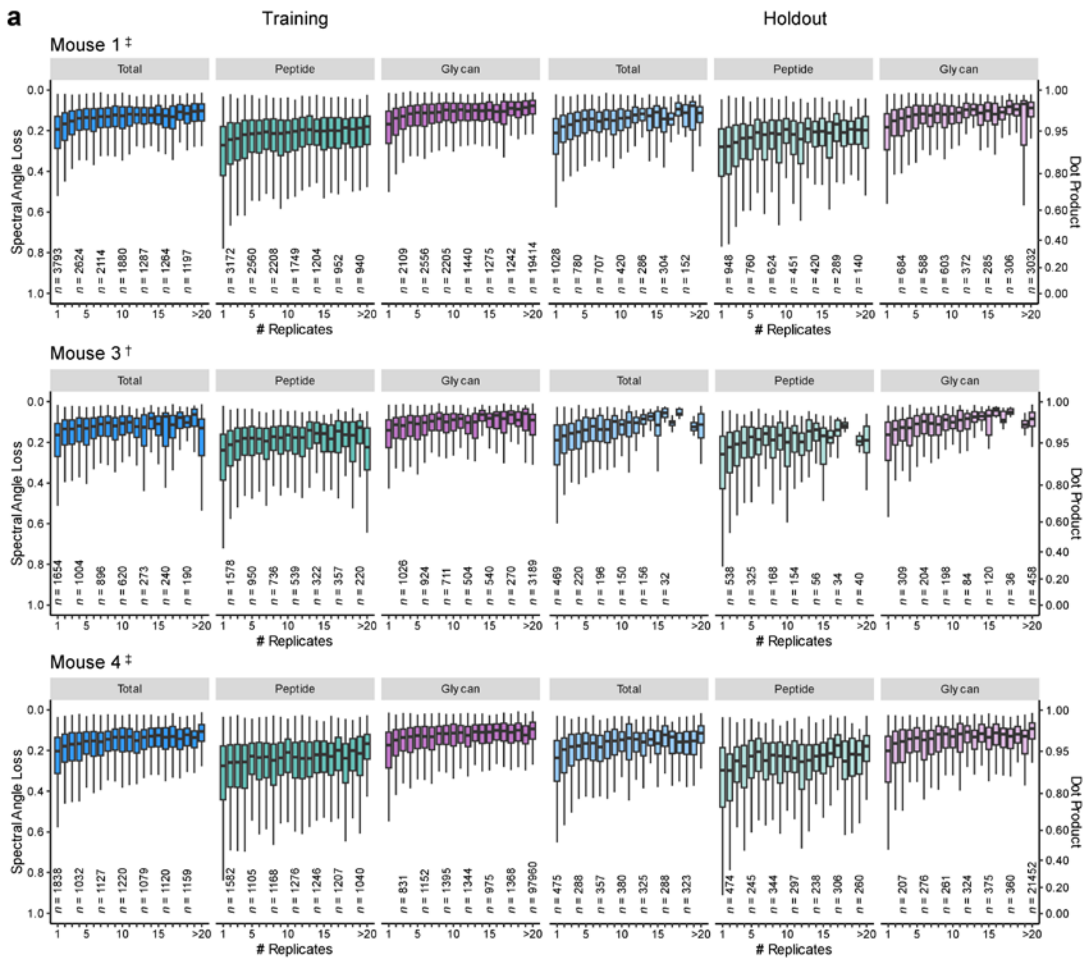

Supplementary Fig. 20. Performance of glycopeptide fragment spectrum prediction using the model trained with a combined dataset evaluated on all replicate spectra.

(**a**) Distributions of spectral similarities between predicted and experimental fragment ion intensities for glycopeptides contained in the three mouse datasets that have been merged into the combined dataset. Related to **Supplementary Fig. 19a**. (Continued on next page)

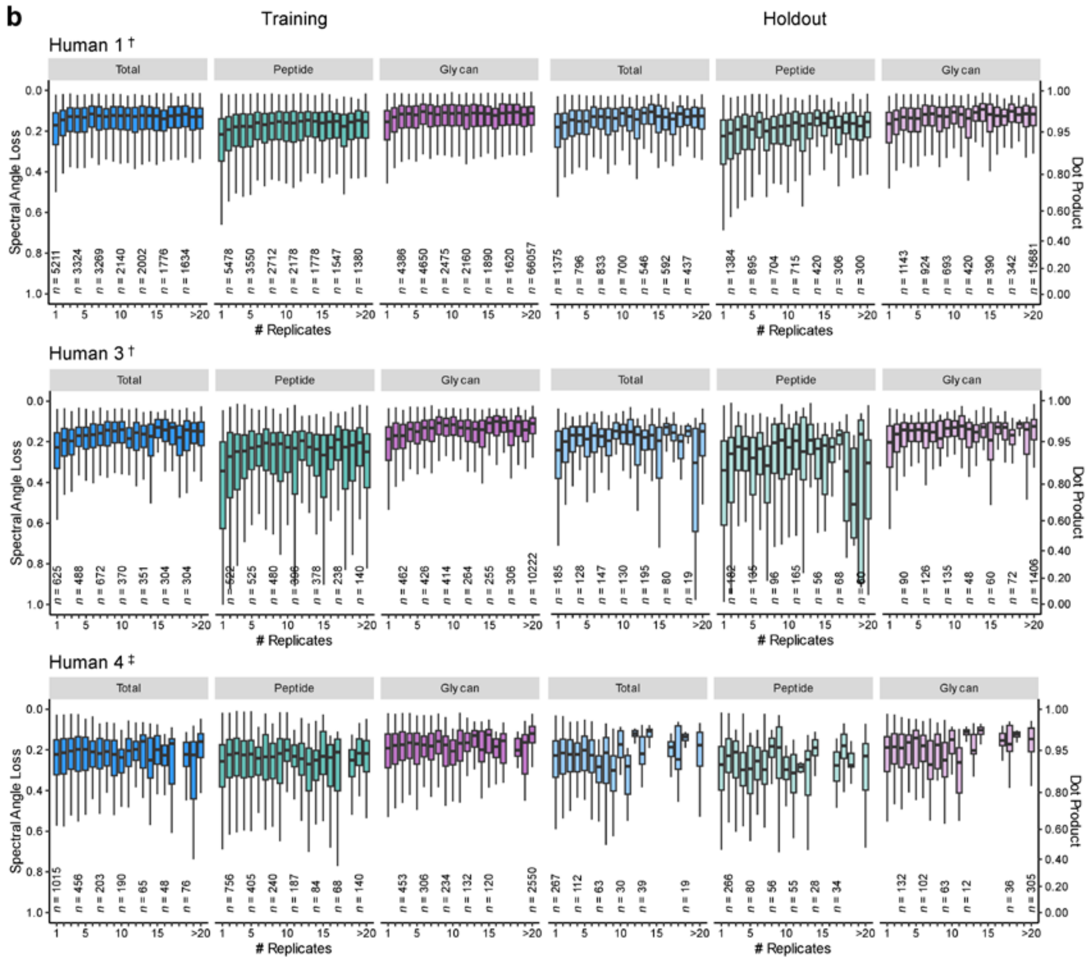

Supplementary Fig. 20 (Continued).

(**a**) Results for glycopeptides contained in the three human datasets that have been merged into the combined dataset. Related to **Supplementary Fig. 19a**. (Continued on next page)

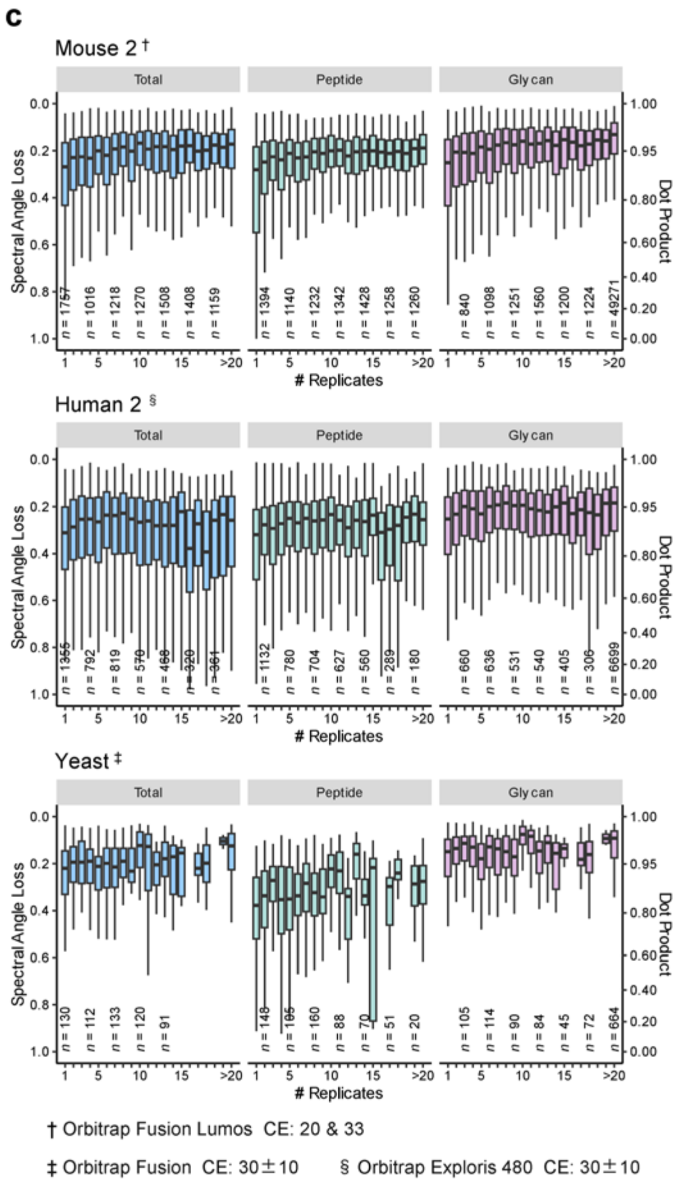

Supplementary Fig. 20 (Continued).

(**c**) Results tested on the other three datasets. Related to **Supplementary Fig. 19b**. The center lines indicate the median values. The lower/upper hinges of the boxes indicate the first/third quartiles, and the lower/upper whiskers extend from the hinges to the smallest/largest value no further than 1.5 times the interquartile range. The data are grouped by the number of replicate spectra of each glycopeptide precursor, and the number of spectra (*n*) in each group is indicated. Source data are provided as a Source Data file.

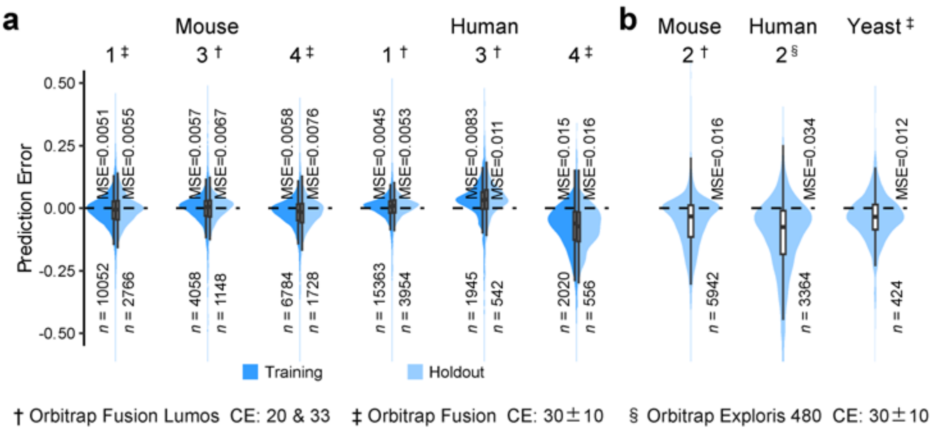

Supplementary Fig. 21. Prediction error of intensity ratio between peptide and glycan fragments using the model trained with a combined dataset evaluated on consensus spectra.

(**a**) Distributions of prediction error between predicted and experimental intensity ratio values for glycopeptides contained in the six datasets that have been merged into the combined dataset. (**b**) Results tested on the other three datasets. The mean squared error (MSE) and data size (*n*) are indicated. The lower/upper hinges of the boxes indicate the first/third quartiles, and the lower/upper whiskers extend from the hinges to the smallest/largest value no further than 1.5 times the interquartile range. Source data are provided as a Source Data file.

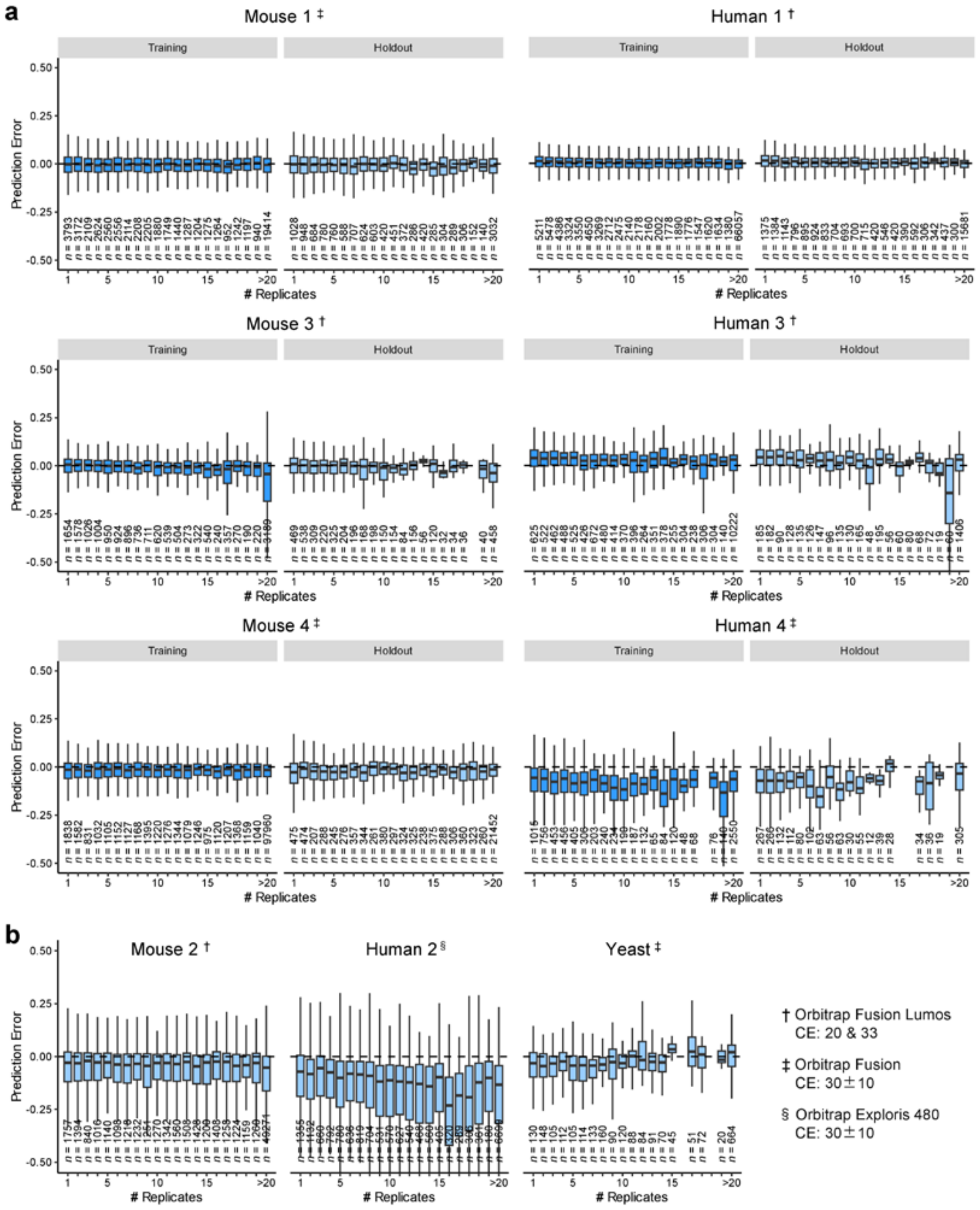

Supplementary Fig. 22. Prediction error of intensity ratio between peptide and glycan fragments using the model trained with a combined dataset evaluated on all replicate spectra.

(**a**) Distributions of prediction error between predicted and experimental intensity ratio values for glycopeptides contained in the six datasets that have been merged into the combined dataset. (**b**) Results tested on the other three datasets. Related to **Supplementary Fig. 21**. The center lines indicate the median values. The lower/upper hinges of the boxes indicate the first/third quartiles, and the lower/upper whiskers extend from the hinges to the smallest/largest value no further than 1.5 times the interquartile range. The data are grouped by the number of replicate spectra of each glycopeptide precursor, and the number of spectra (*n*) in each group is indicated. Source data are provided as a Source Data file.

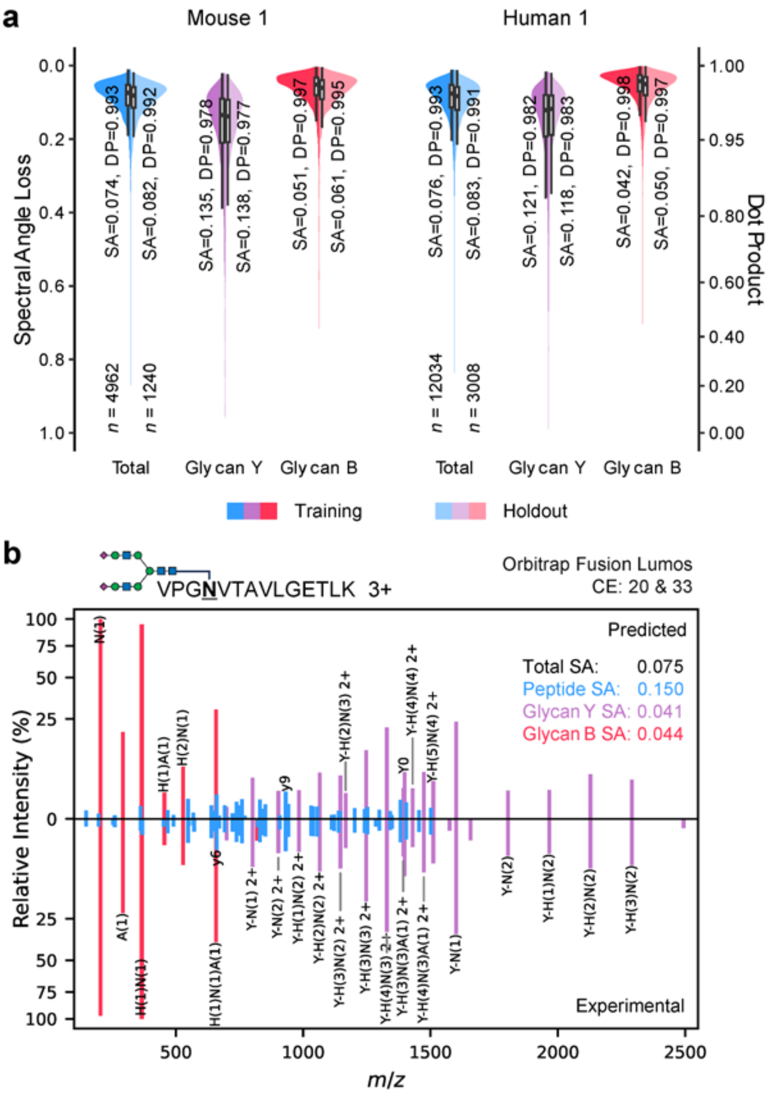

Supplementary Fig. 23. Performance of glycopeptide fragment spectrum prediction using models with B ions evaluated on consensus spectra.

(**a**) Distributions of spectral similarities between predicted and experimental fragment ion intensities for glycopeptides contained in the training or holdout set of Mouse1 and Human1. Glycopeptides with high-mannose type glycans are excluded. Spectral similarities are computed for glycan Y ions and B ions separately, as well as for the total spectrum of peptide b/y ions, glycan Y ions and B ions. The median values of spectral angle loss (SA) and dot product (DP), as well as data size (*n*), are indicated. The lower/upper hinges of the boxes indicate the first/third quartiles, and the lower/upper whiskers extend from the hinges to the smallest/largest value no further than 1.5 times the interquartile range. (**b**) Mirror plot of a glycopeptide spectral match comparing predicted fragment intensities to experimental fragment intensities in Human1. Source data are provided as a Source Data file.

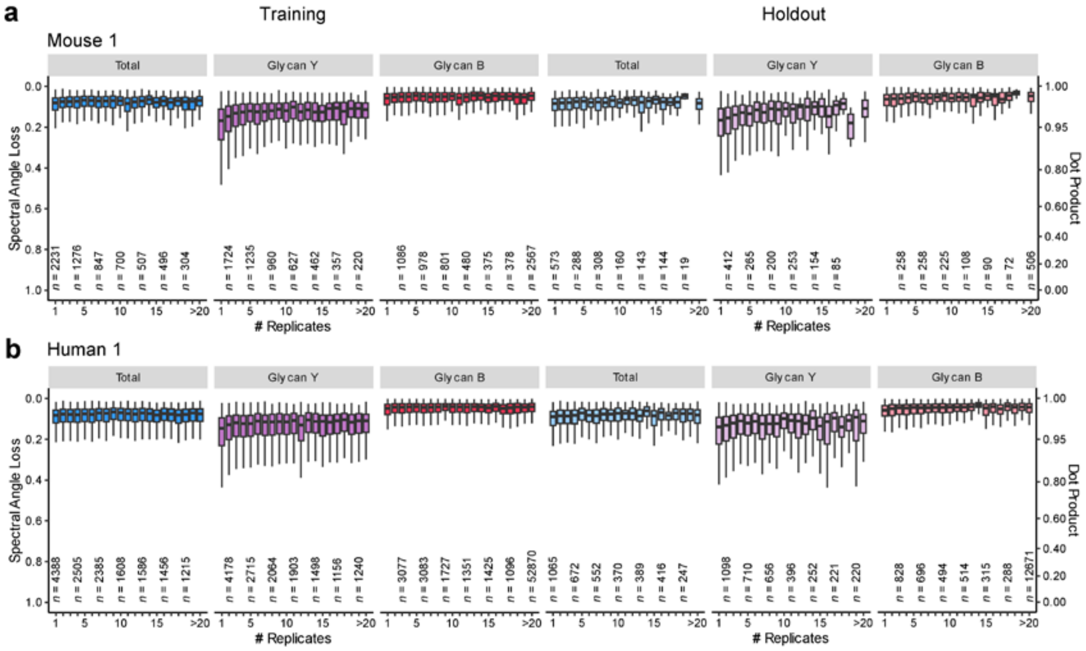

Supplementary Fig. 24. Performance of glycopeptide fragment spectrum prediction using models with B ions evaluated on all replicate spectra.

(**a**) Distributions of spectral similarities between predicted and experimental fragment ion intensities for glycopeptides contained in the training or holdout subset of Mouse1. (**b**) Distributions of spectral similarities for Human1. Related to **Supplementary Fig. 23**. The center lines indicate the median values. The lower/upper hinges of the boxes indicate the first/third quartiles, and the lower/upper whiskers extend from the hinges to the smallest/largest value no further than 1.5 times the interquartile range. The data are grouped by the number of replicate spectra of each glycopeptide precursor, and the number of spectra (*n*) in each group is indicated. Source data are provided as a Source Data file.

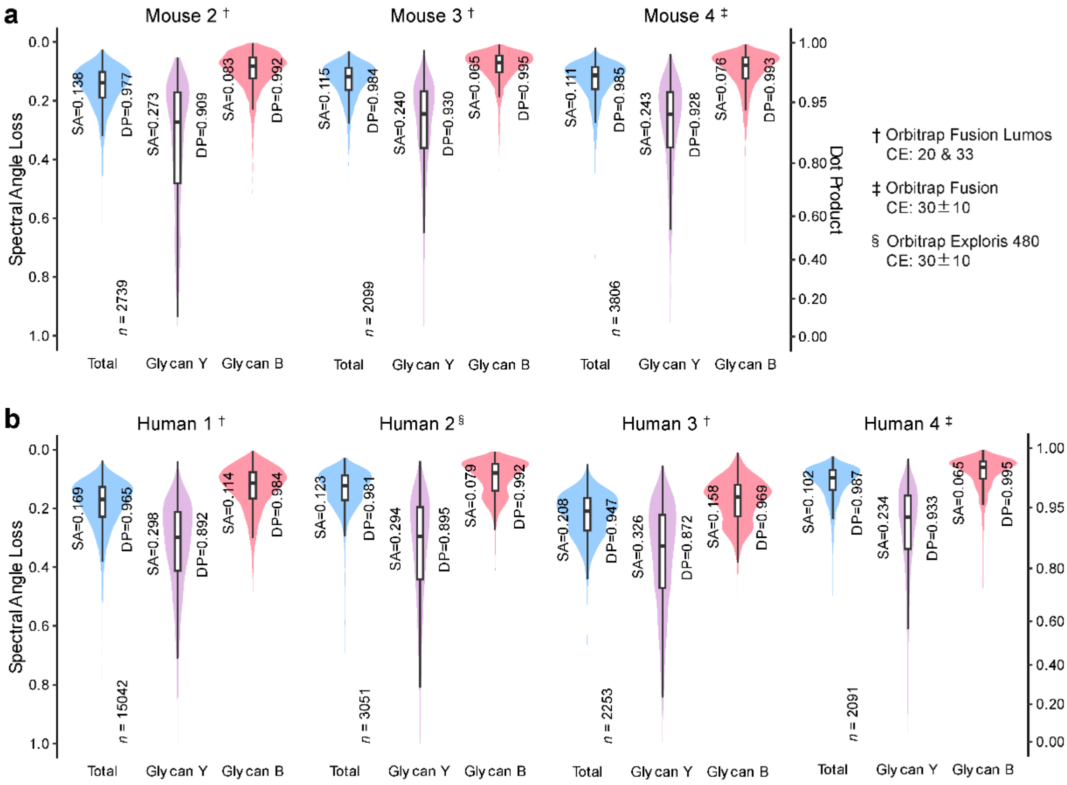

Supplementary Fig. 25. Performance of glycopeptide fragment spectrum prediction using a model with B ions trained with Mouse1 evaluated on consensus spectra.

(**a**) Distributions of spectral similarities between predicted and experimental fragment ion intensities, tested on other mouse datasets. (**b**) Results tested on human datasets. Glycopeptides with high-mannose type glycans are excluded. Instrument settings of each dataset are marked. Spectral similarities are computed for glycan Y ions and B ions separately, as well as for the total spectrum of peptide b/y ions, glycan Y ions and B ions. The median values of spectral angle loss (SA) and dot product (DP), as well as data size (*n*), are indicated. The lower/upper hinges of the boxes indicate the first/third quartiles, and the lower/upper whiskers extend from the hinges to the smallest/largest value no further than 1.5 times the interquartile range. Source data are provided as a Source Data file.

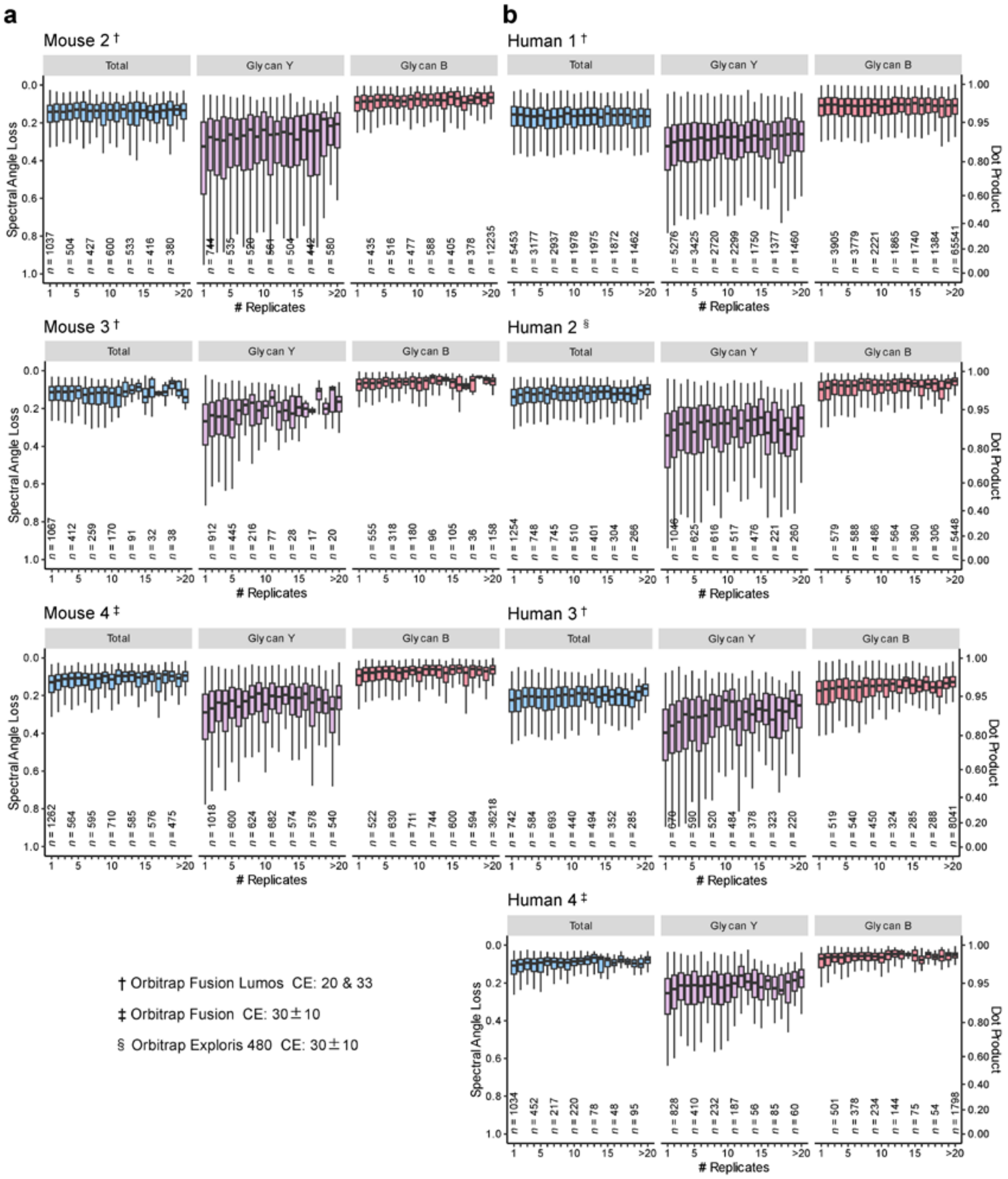

Supplementary Fig. 26. Performance of glycopeptide fragment spectrum prediction using the model with B ions trained with Mouse1 evaluated on all replicate spectra.

(**a**) Distributions of spectral similarities between predicted and experimental fragment ion intensities, tested on other mouse datasets. (**b**) Results tested on human datasets. Related to **Supplementary Fig. 25**. The center lines indicate the median values. The lower/upper hinges of the boxes indicate the first/third quartiles, and the lower/upper whiskers extend from the hinges to the smallest/largest value no further than 1.5 times the interquartile range. The data are grouped by the number of replicate spectra of each glycopeptide precursor, and the number of spectra (*n*) in each group is indicated. Source data are provided as a Source Data file.

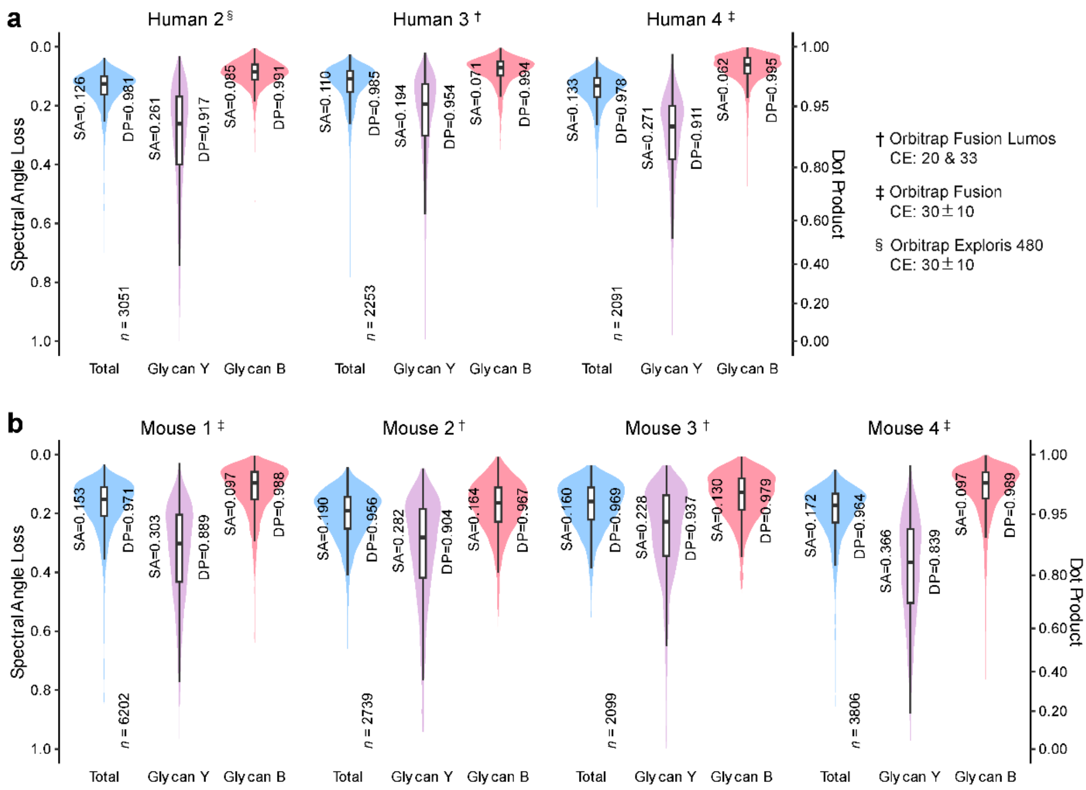

Supplementary Fig. 27. Performance of glycopeptide fragment spectrum prediction using a model with B ions trained with Human1 evaluated on consensus spectra.

(**a**) Distributions of spectral similarities between predicted and experimental fragment ion intensities, tested on other human datasets. (**b**) Results tested on mouse datasets. Glycopeptides with high-mannose type glycans are excluded. Instrument settings of each dataset are marked. Spectral similarities are computed for glycan Y ions and B ions separately, as well as for the total spectrum of peptide b/y ions, glycan Y ions and B ions. The median values of spectral angle loss (SA) and dot product (DP), as well as data size (*n*), are indicated. The lower/upper hinges of the boxes indicate the first/third quartiles, and the lower/upper whiskers extend from the hinges to the smallest/largest value no further than 1.5 times the interquartile range. Source data are provided as a Source Data file.

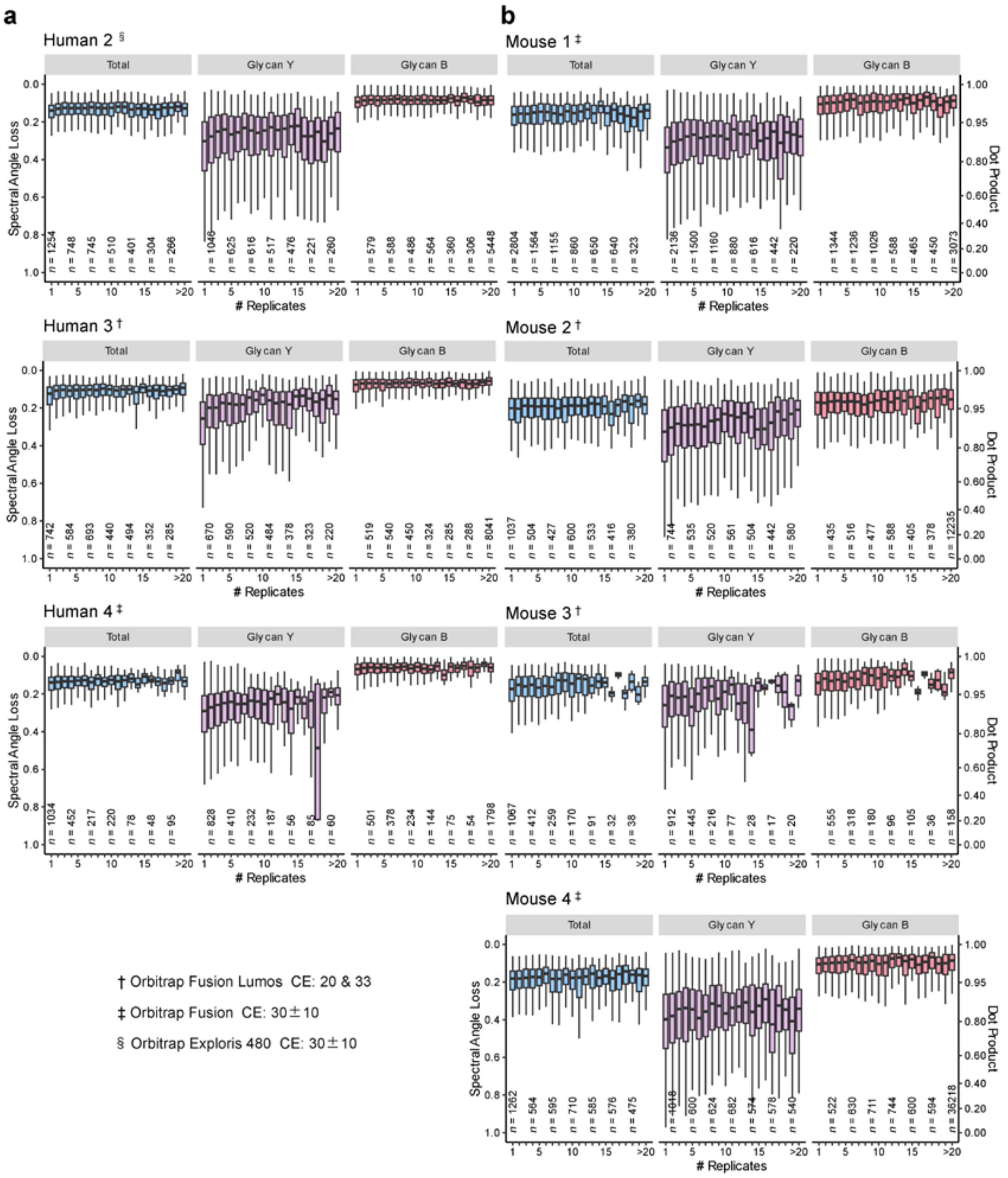

Supplementary Fig. 28. Performance of glycopeptide fragment spectrum prediction using the model with B ions trained with Human1 evaluated on all replicate spectra.

(**a**) Distributions of spectral similarities between predicted and experimental fragment ion intensities, tested on other human datasets. (**b**) Results tested on mouse datasets. Related to **Supplementary Fig. 27**. The center lines indicate the median values. The lower/upper hinges of the boxes indicate the first/third quartiles, and the lower/upper whiskers extend from the hinges to the smallest/largest value no further than 1.5 times the interquartile range. The data are grouped by the number of replicate spectra of each glycopeptide precursor, and the number of spectra (*n*) in each group is indicated. Source data are provided as a Source Data file.

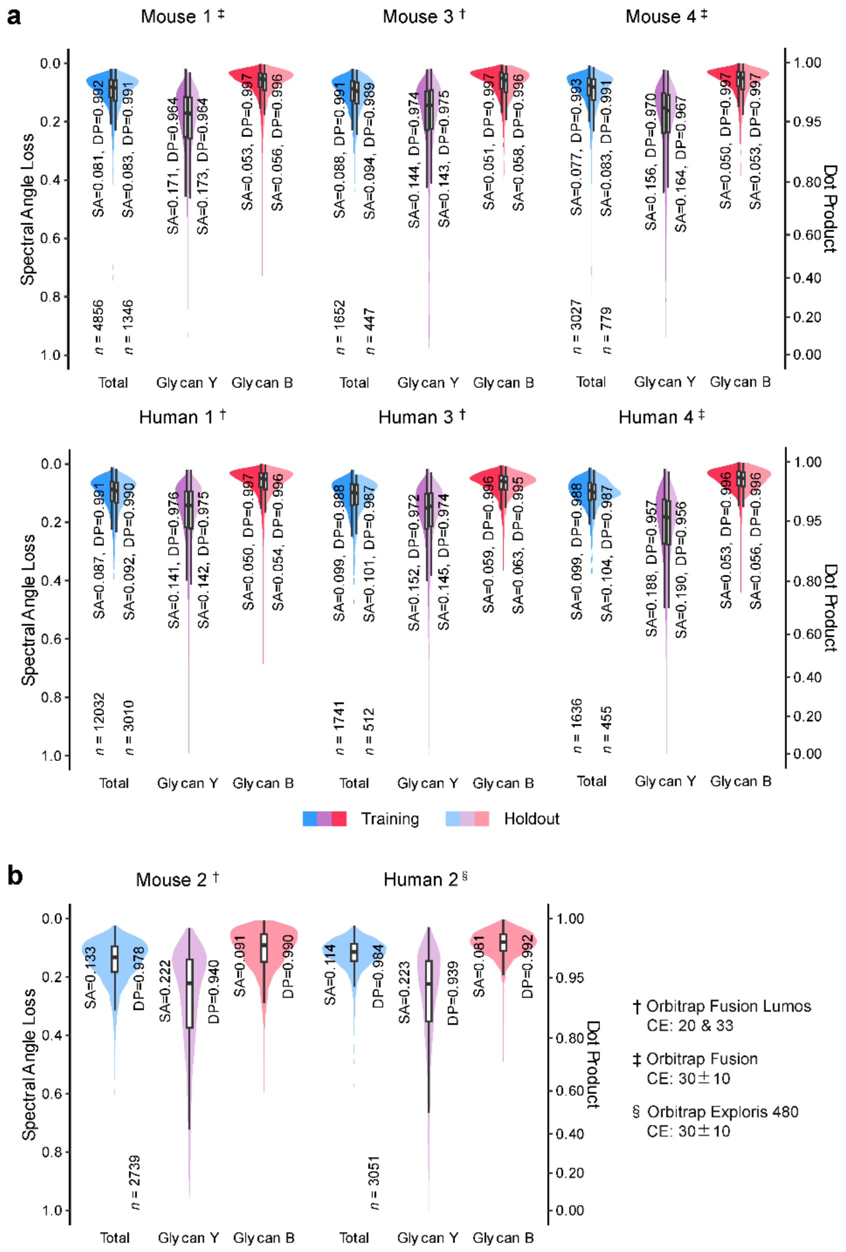

Supplementary Fig. 29. Performance of glycopeptide fragment spectrum prediction using a model with B ions trained with a combined dataset evaluated on consensus spectra.

(**a**) Distributions of spectral similarities between predicted and experimental fragment ion intensities for glycopeptides contained in the six datasets that have been merged into the combined dataset. (**b**) Results tested on the other two datasets. Instrument settings of each dataset are marked. Spectral similarities are computed for glycan Y ions and B ions separately, as well as for the total spectrum of peptide b/y ions, glycan Y ions and B ions. The median values of spectral angle loss (SA) and dot product (DP), as well as data size (*n*), are indicated. The lower/upper hinges of the boxes indicate the first/third quartiles, and the lower/upper whiskers extend from the hinges to the smallest/largest value no further than 1.5 times the interquartile range. Source data are provided as a Source Data file.

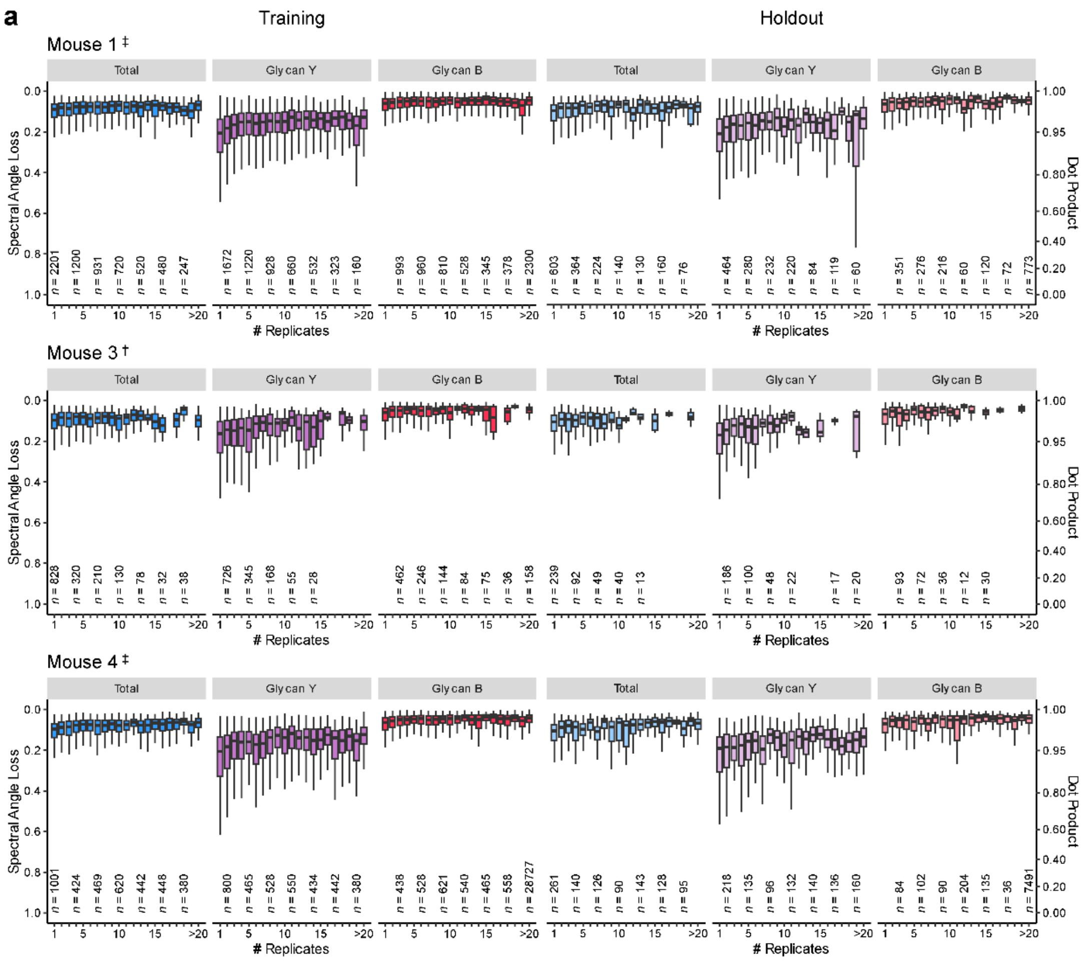

Supplementary Fig. 30. Performance of glycopeptide fragment spectrum prediction using the model with B ions trained with a combined dataset evaluated on all replicate spectra.

(**a**) Distributions of spectral similarities between predicted and experimental fragment ion intensities for glycopeptides contained in the three mouse datasets that have been merged into the combined dataset. Related to **Supplementary Fig. 29a**. (Continued on next page)

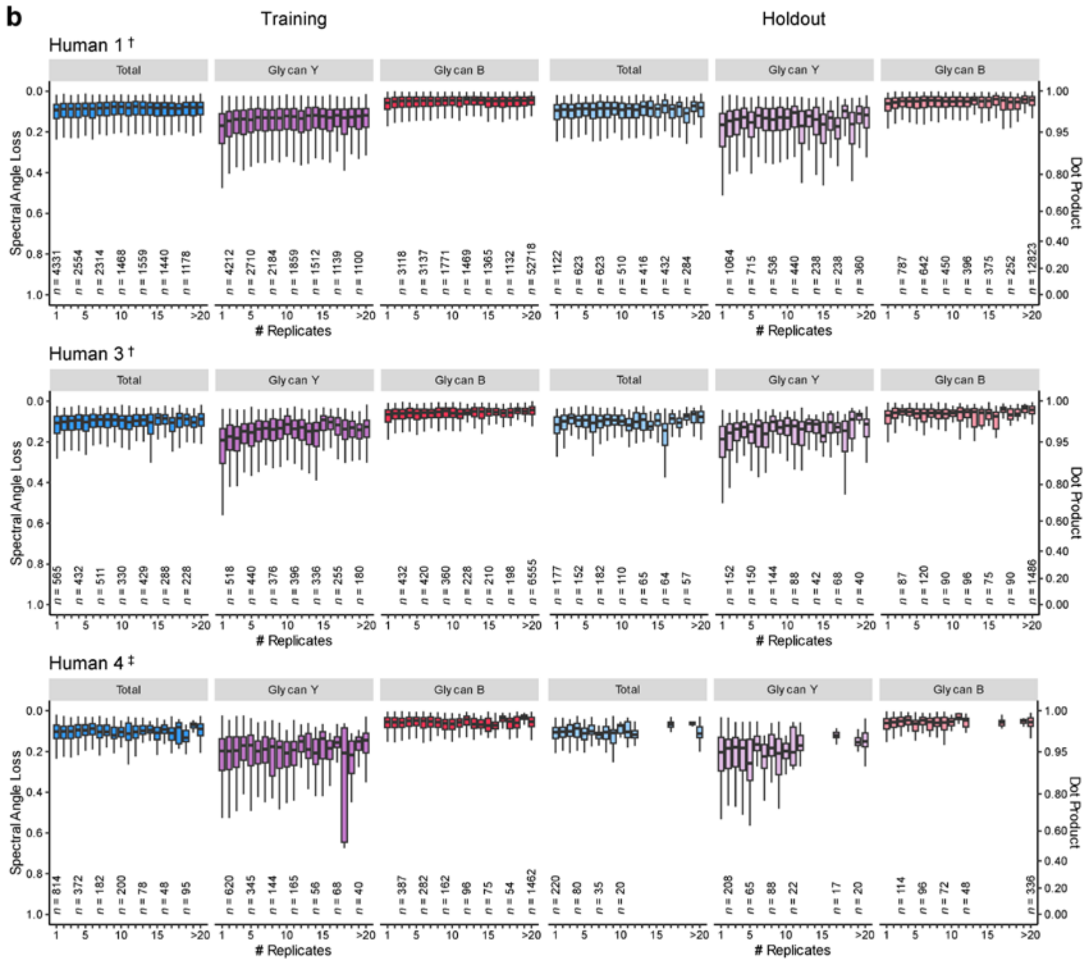

Supplementary Fig. 30 (Continued).

(**a**) Results for glycopeptides contained in the three human datasets that have been merged into the combined dataset. Related to **Supplementary Fig. 29a**. (Continued on next page)

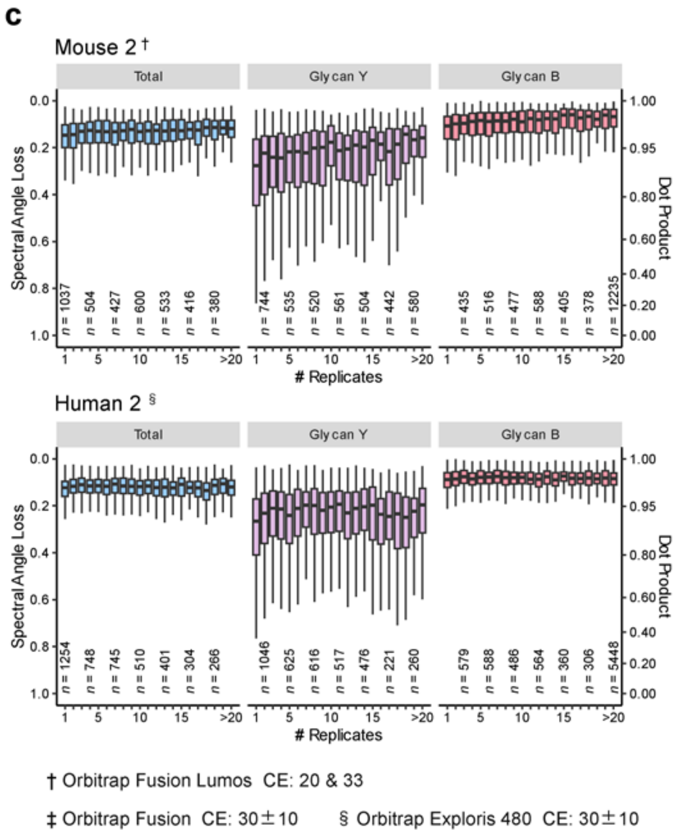

Supplementary Fig. 30 (Continued).

(**c**) Results tested on the other two datasets. Related to **Supplementary Fig. 29b**. The center lines indicate the median values. The lower/upper hinges of the boxes indicate the first/third quartiles, and the lower/upper whiskers extend from the hinges to the smallest/largest value no further than 1.5 times the interquartile range. The data are grouped by the number of replicate spectra of each glycopeptide precursor, and the number of spectra (*n*) in each group is indicated. Source data are provided as a Source Data file.

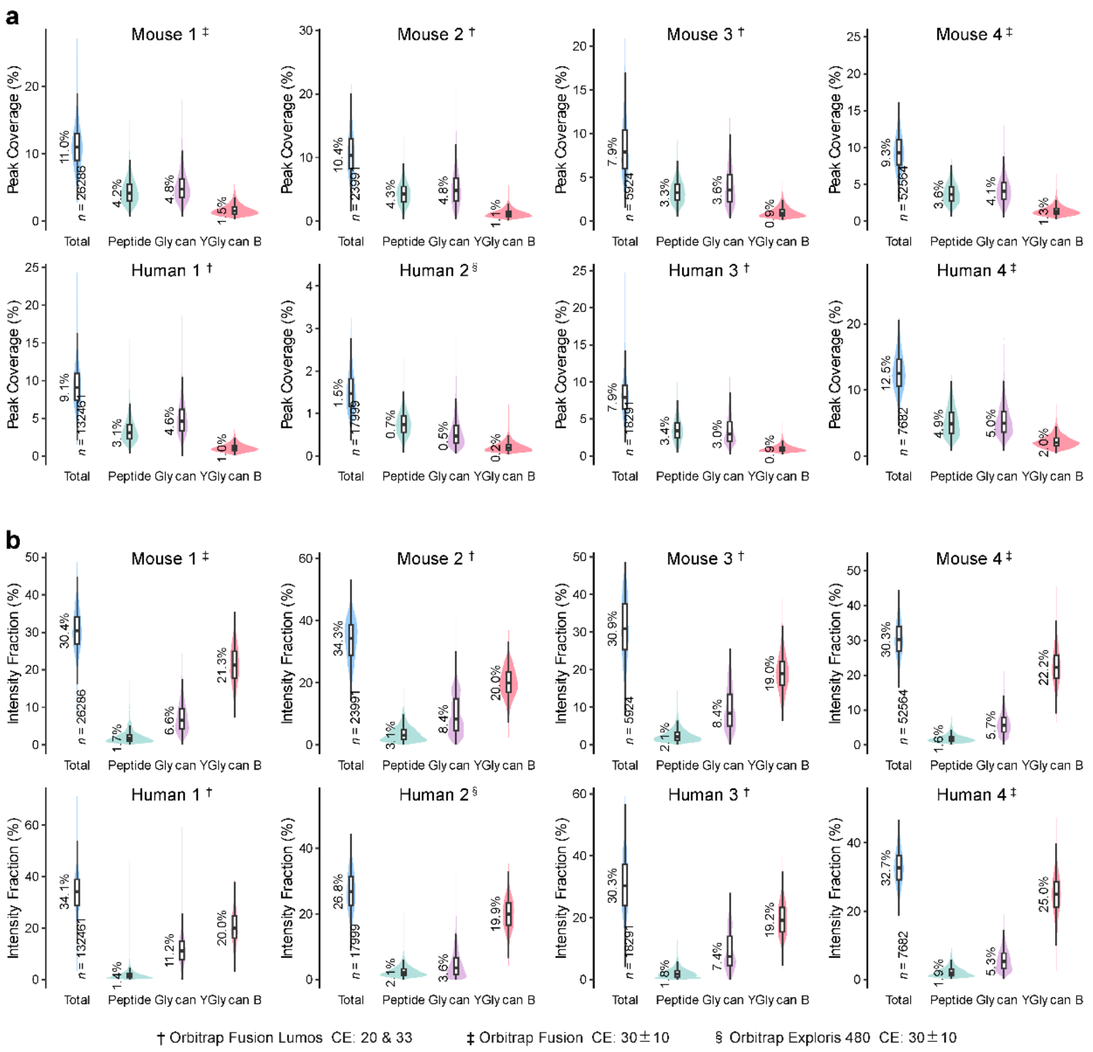

Supplementary Fig. 31. Coverage of the fragment peaks that were considered in this study among the raw experimental spectra.

(**a**) The coverage of peak numbers. (**b**) The fraction of the summed intensity of the covered peaks. The center lines indicate the median values. The lower/upper hinges of the boxes indicate the first/third quartiles, and the lower/upper whiskers extend from the hinges to the smallest/largest value no further than 1.5 times the interquartile range. The number of spectra (*n*) is indicated. Source data are provided as a Source Data file.

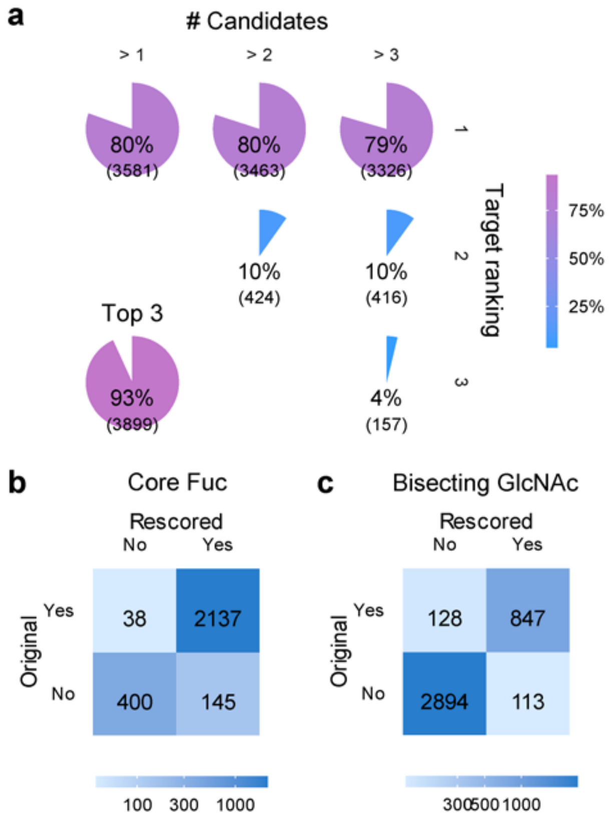

Supplementary Fig. 32. Differentiating structural isomeric glycopeptides from the holdout set of Mouse1 using predicted spectral libraries.

(**a**) Candidate ranking results. The percentage of spectra in which the correct identity was ranked as the first, second or third candidate are calculated out of the number of cases with more than 1, 2 or 3 candidates in total. The top-three chart shows the percentage of cases in which the correct identity is ranked among the top three candidates of the total of cases with more than three candidates. (**b**) Confusion matrix of core fucosylation recognition. (**c**) Confusion matrix of bisecting HexNAc recognition. GPSMs with all the candidates belonging to the same category are excluded when calculating the confusion matrices.

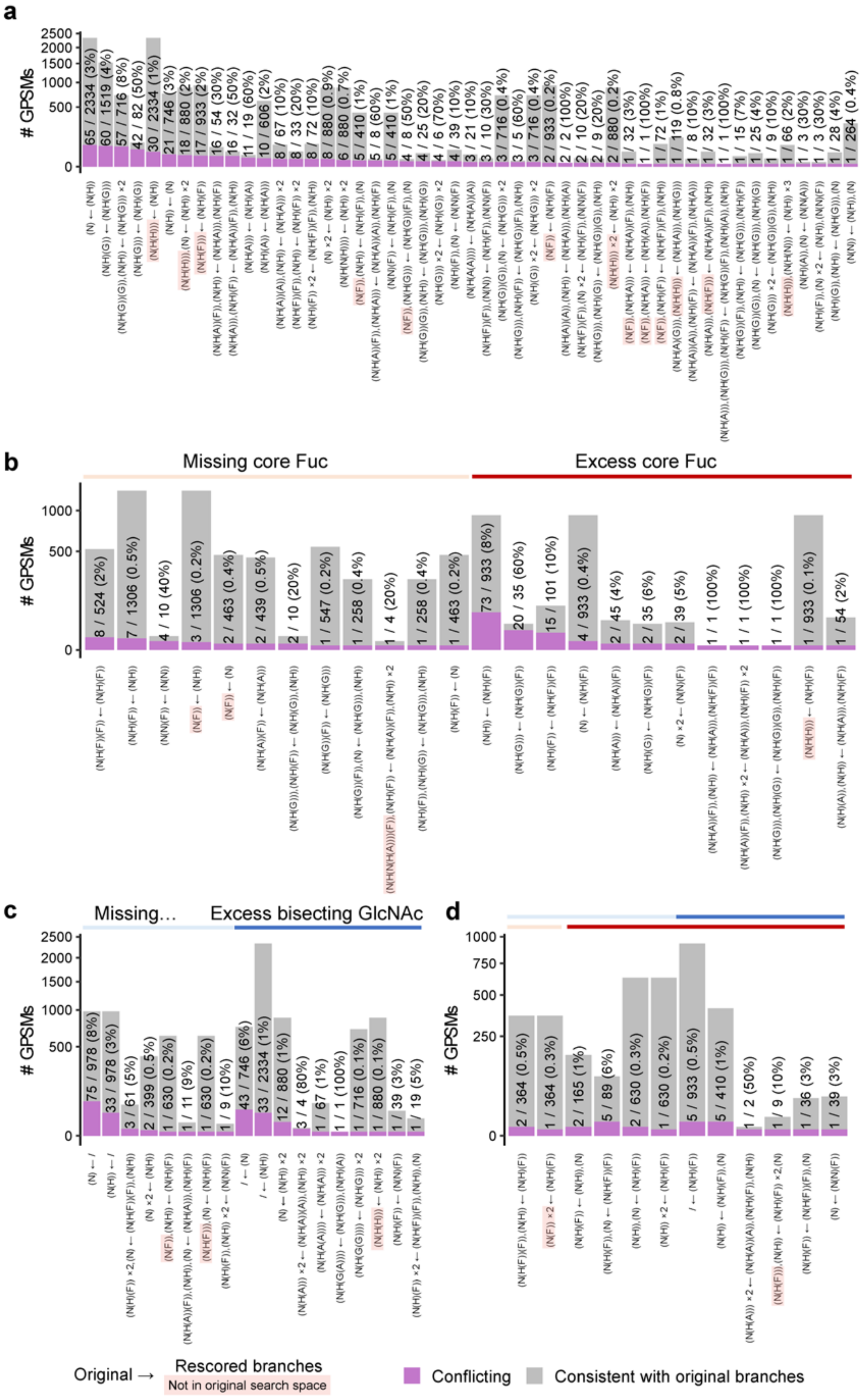
a
GPSMs
b
Missing core Fuc
Excess core Fuc
c
Missing…
Excess bisecting GlcNAc
d
Original → Rescored branches
Not in original search space
Conflicting
Consistent with original branches

Supplementary Fig. 33. Conflicting results between spectral library searching and the original StrucGP annotations from the holdout set of Mouse1.

(**a**) Results differing in branch identification only. (**b**) Results differing in bisecting HexNAc recognition. (**c**) Results differing in core fucosylation recognition. (**d**) Results differing in both bisecting HexNAc and core fucosylation recognition. Results are categorized into types of conflictions (labels of x-axis), defined by glycan branches (in pGlyco format) of the original annotations and the top candidates rescored by spectral library searching. For each type of branch confliction, the percentage of GPSMs with conflicting results (purple) is calculated out of those that have a possibility of this type of confliction, i.e., all the GPSMs whose original annotation includes the original glycan branches (as well as core fucosylation and bisecting HexNAc if any) of the type of confliction (purple + grey).

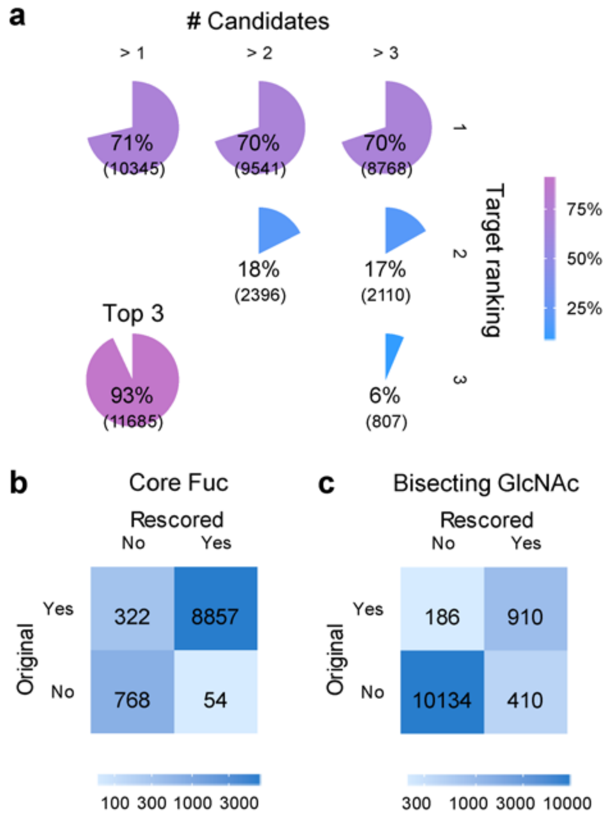

Supplementary Fig. 34. Differentiating structural isomeric glycopeptides from the holdout set of Human1 using predicted spectral libraries.

(**a**) Candidate ranking results. The percentage of spectra in which the correct identity was ranked as the first, second or third candidate are calculated out of the number of cases with more than 1, 2 or 3 candidates in total. The top-three chart shows the percentage of cases in which the correct identity is ranked among the top three candidates of the total of cases with more than three candidates. (**b**) Confusion matrix of core fucosylation recognition. (**c**) Confusion matrix of bisecting HexNAc recognition. GPSMs with all the candidates belonging to the same category are excluded when calculating the confusion matrices.

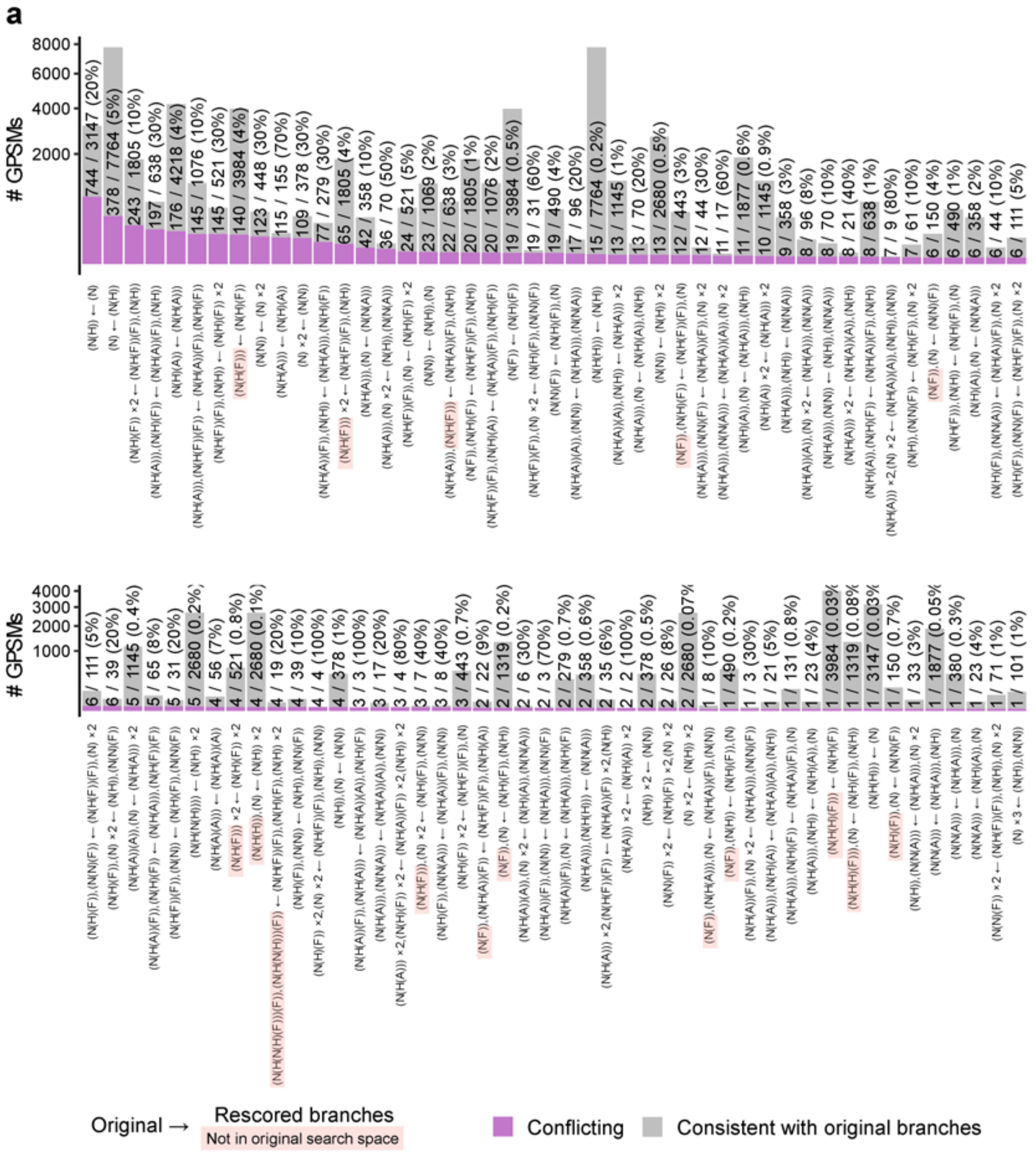

Supplementary Fig. 35. Conflicting results between spectral library searching and the original StrucGP annotations from the holdout set of Human1.

(**a**) Results differing in branch identification only. (Continued on next page)

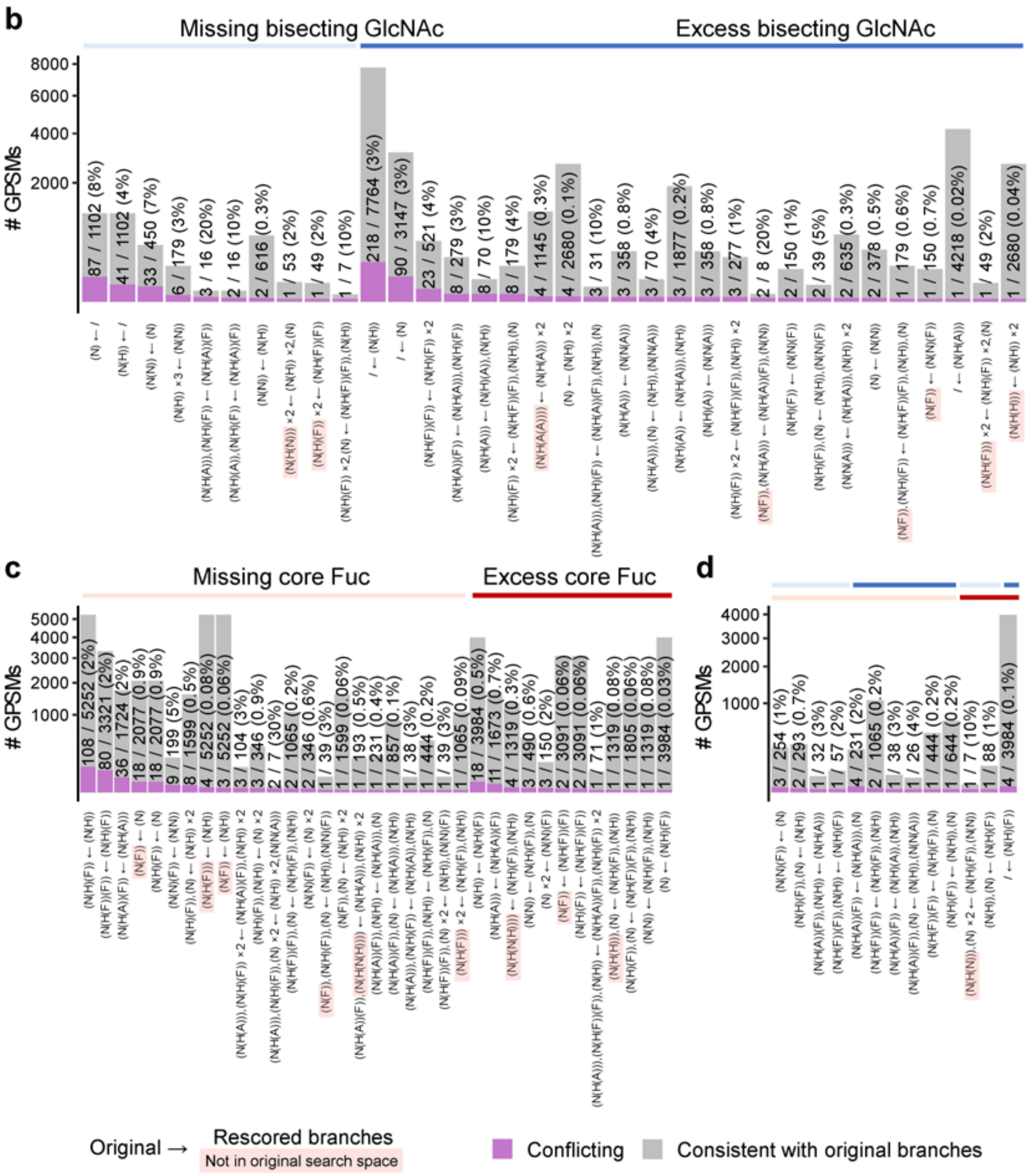

Supplementary Fig. 35 (Continued).

(**b**) Results differing in bisecting HexNAc recognition. (**c**) Results differing in core fucosylation recognition. (**d**) Results differing in both bisecting HexNAc and core fucosylation recognition. Results are categorized into types of conflictions (labels of x-axis), defined by glycan branches (in pGlyco format) of the original annotations and the top candidates rescored by spectral library searching. For each type of branch confliction, the percentage of GPSMs with conflicting results (purple) is calculated out of those that have a possibility of this type of confliction, i.e., all the GPSMs whose original annotation includes the original glycan branches (as well as core fucosylation and bisecting HexNAc if any) of the type of confliction (purple + grey).

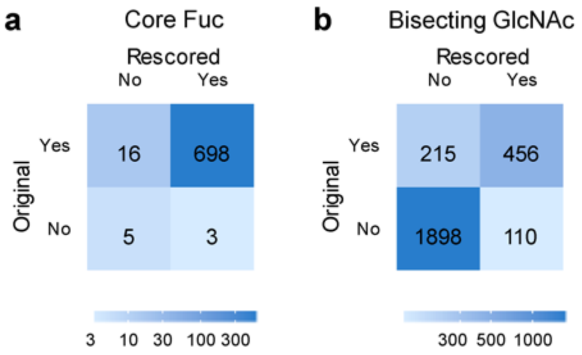

Supplementary Fig. 36. Differentiating structural isomeric glycopeptides from the standard glycoprotein dataset using predicted spectral libraries.

(**a**) Confusion matrix of core fucosylation recognition. (**b**) Confusion matrix of bisecting HexNAc recognition. GPSMs with all the candidates belonging to the same category are excluded when calculating the confusion matrices.

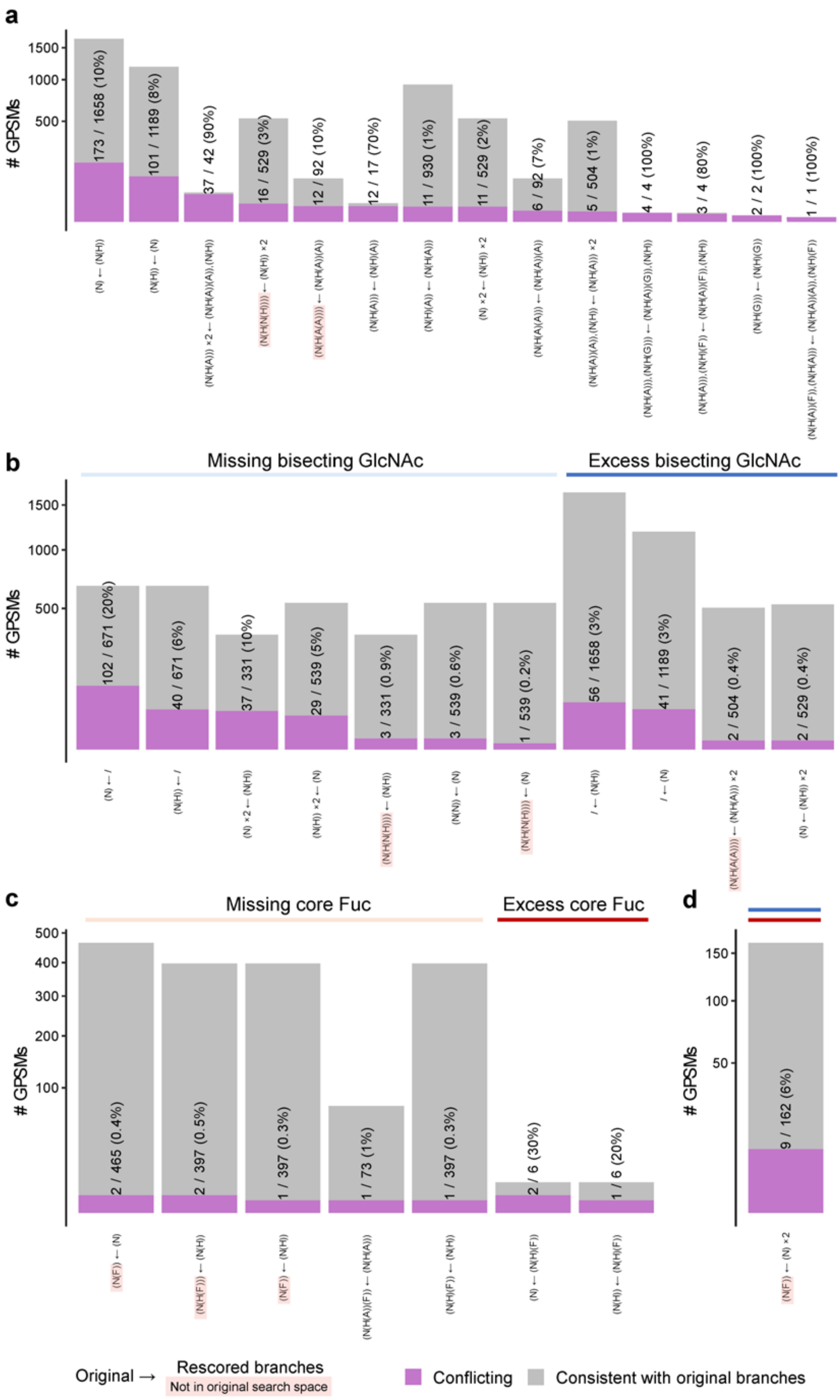
a
GPSMs
1500
1000
500
173 / 1658 (10%)
101 / 1189 (8%)
37 / 42 (90%)
16 / 529 (3%)
12 / 92 (10%)
12 / 17 (70%)
11 / 930 (1%)
11 / 529 (2%)
6 / 92 (7%)
5 / 504 (1%)
4 / 4 (100%)
3 / 4 (80%)
2 / 2 (100%)
1 / 1 (100%)
b
Missing bisecting GlcNAc
Excess bisecting GlcNAc
GPSMs
1500
1000
500
102 / 671 (20%)
40 / 671 (6%)
37 / 331 (10%)
29 / 539 (5%)
3 / 331 (0.9%)
3 / 539 (0.6%)
1 / 539 (0.2%)
56 / 1658 (3%)
41 / 1189 (3%)
2 / 504 (0.4%)
2 / 529 (0.4%)
c
Missing core Fuc
Excess core Fuc
GPSMs
500
400
300
200
100
2 / 465 (0.4%)
2 / 397 (0.5%)
1 / 397 (0.3%)
1 / 73 (1%)
1 / 397 (0.3%)
2 / 6 (30%)
1 / 6 (20%)
d
GPSMs
150
100
50
9 / 162 (6%)
Original →
Rescored branches
Not in original search space
Conflicting
Consistent with original branches

Supplementary Fig. 37. Conflicting results between spectral library searching and the original StrucGP annotations from the standard glycoprotein dataset.

(**a**) Results differing in branch identification only. (**b**) Results differing in bisecting HexNAc recognition. (**c**) Results differing in core fucosylation recognition. (**d**) Results differing in both bisecting HexNAc and core fucosylation recognition. Results are categorized into types of confliction (labels of x-axis), defined by glycan branches (in pGlyco format) of the original annotations and the top candidates rescored by spectral library searching. For each type of branch confliction, the percentage of GPSMs with conflicting results (purple) is calculated out of those that have a possibility of this type of confliction, i.e., all the GPSMs whose original annotation includes the original glycan branches (as well as core fucosylation and bisecting HexNAc if any) of the type of confliction (purple + grey).

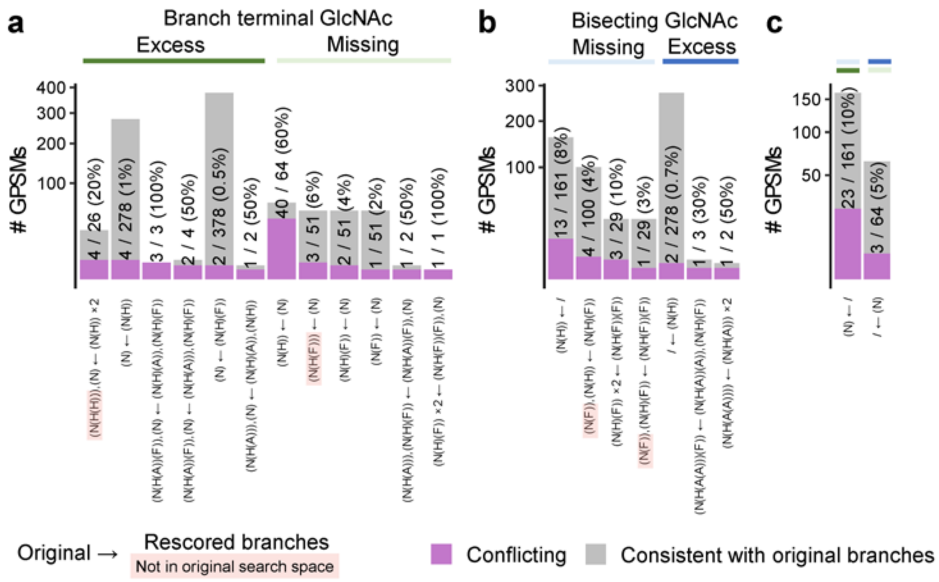

Supplementary Fig. 38. Conflicting results of terminal HexNAc recognition between spectral library searching with the original StrucGP annotations from the exoglycosidase treated mouse brain dataset.

(**a**) Results differing in branch terminal HexNAc recognition. (**b**) Results differing in bisecting HexNAc recognition. (**c**) Results differing in both branch terminal and bisecting HexNAc recognition. Results are categorized into types of conflictions (labels of x-axis), defined by glycan branches (in pGlyco format) of the original annotations and the top candidates rescored by spectral library searching. For each type of branch confliction, the percentage of GPSMs with conflicting results (purple) is calculated out of those that have a possibility of this type of confliction, i.e., all the GPSMs whose original annotation includes the original glycan branches (as well as core fucosylation and bisecting HexNAc if any) of the type of confliction (purple + grey).

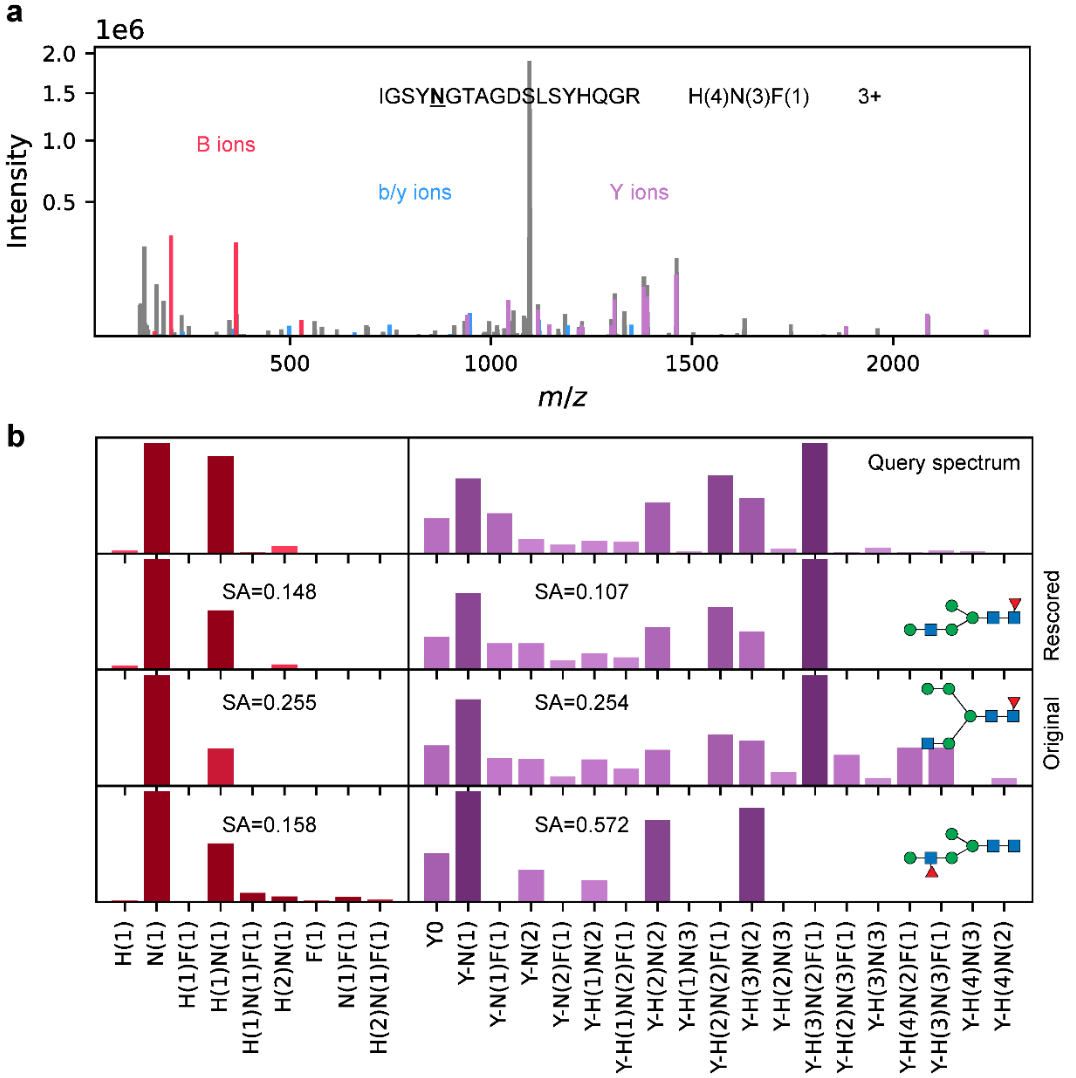

Supplementary Fig. 39. Example spectral match where spectral library searching dismisses the branch terminal HexNAc in the original StrucGP annotations.

(**a**) The query spectrum. (**b**) Intensity patterns of the query spectrum and predicted spectra of the candidate glycan structures. Spectra angle loss (SA) values are indicated for each candidate. Other candidates are not shown.

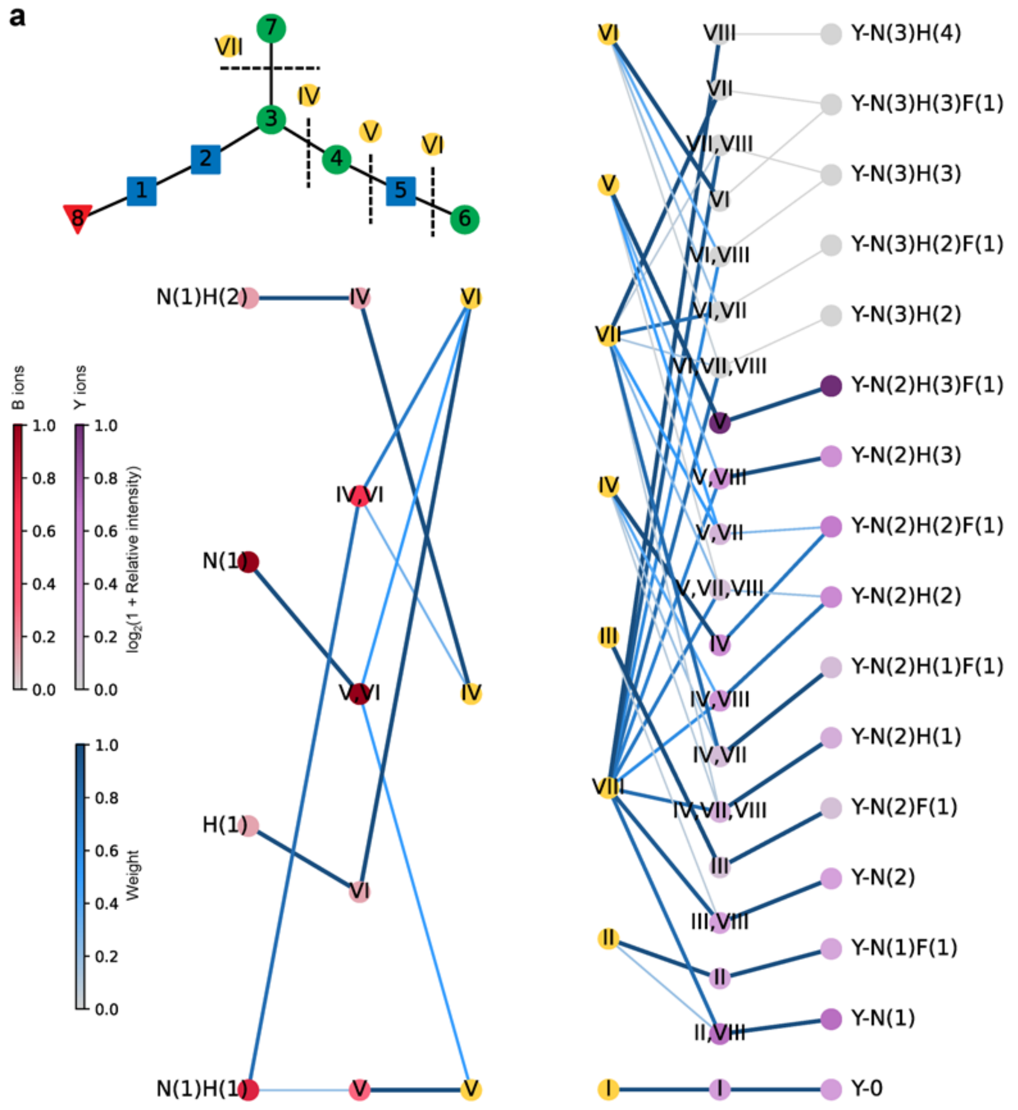

Supplementary Fig. 40. Glycan fragmentation graphs of example glycopeptides with and without a branch terminal HexNAc.

(**a**) The first candidate in **Supplementary Fig. 39** without a branch terminal HexNAc. (Continued on next page)

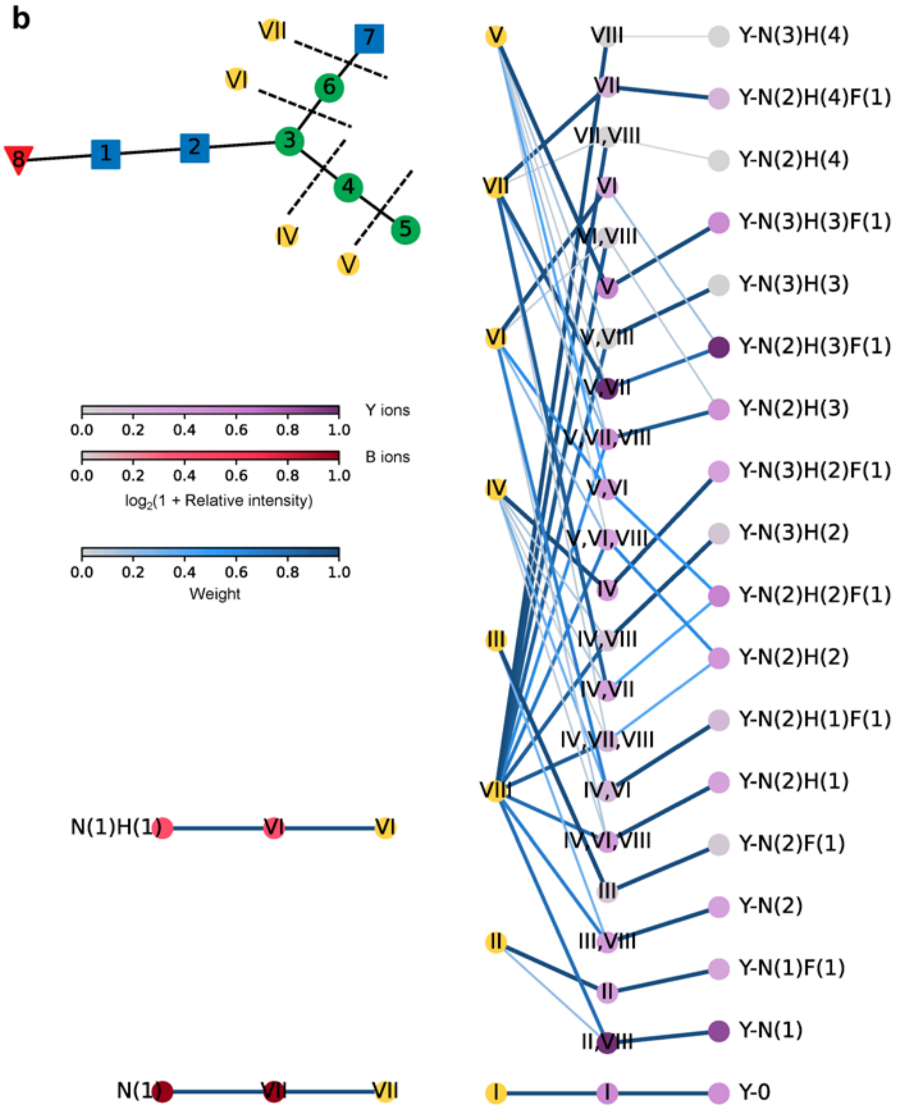

Supplementary Fig. 40 (Continued).

(**b**) The second candidate in **Supplementary Fig. 39** with a branch terminal HexNAc. The cleavage site (yellow nodes) at the non-reducing end of each monosaccharide is represented in Roman numerals. The color of structure-specific fragment nodes and composition-based fragment nodes indicated the relative intensity of B ions (red) or Y ions (purple). The color of edges linking cleavage nodes to structure-specific fragment nodes indicates the attention weights. The color of edges linking structure-specific fragment nodes to composition-based fragment nodes indicates the proportion of intensity that each isomeric fragment contributes to the MS peak.

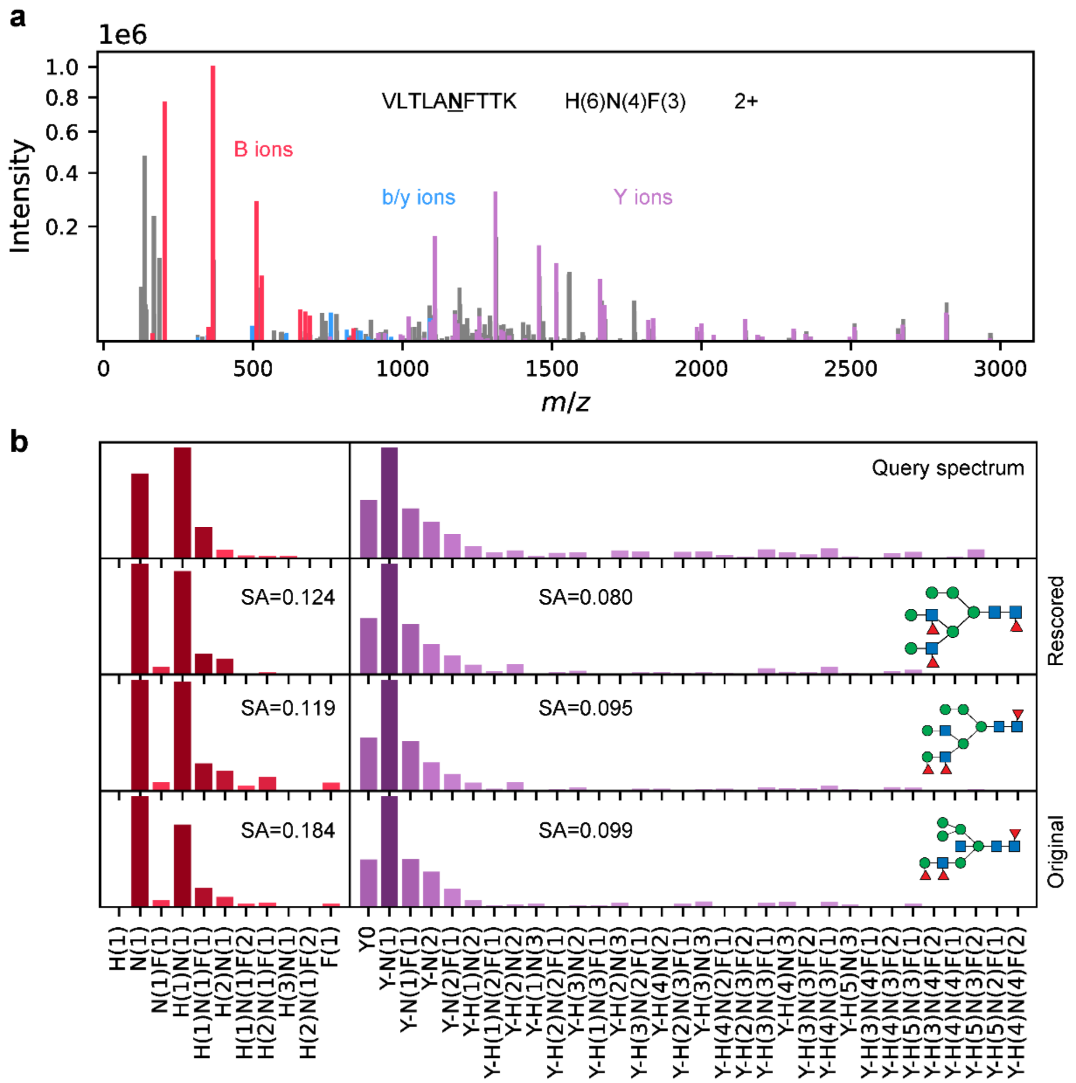

Supplementary Fig. 41. Example spectral match where spectral library searching dismisses the bisecting HexNAc in the original StrucGP annotations.

(**a**) The query spectrum. (**b**) Intensity patterns of the query spectrum and predicted spectra of the candidate glycan structures. Spectra angle loss (SA) values are indicated for each candidate. Other candidates are not shown.

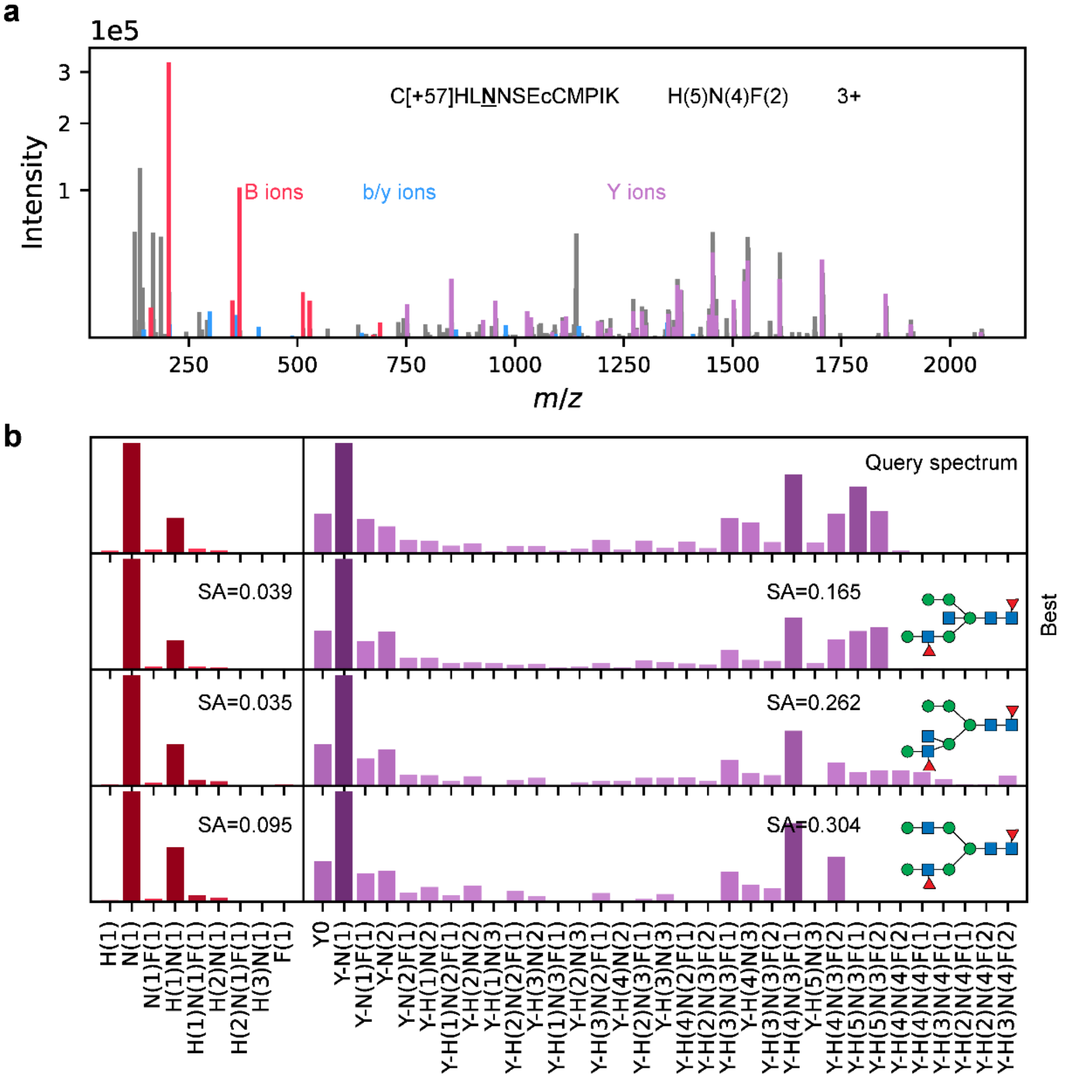

Supplementary Fig. 42. Example spectral match where spectral library searching confirms the bisecting HexNAc in the original StrucGP annotations.

(**a**) The query spectrum. (**b**) Intensity patterns of the query spectrum and predicted spectra of the candidate glycan structures. Spectra angle loss (SA) values are indicated for each candidate. Other candidates are not shown.

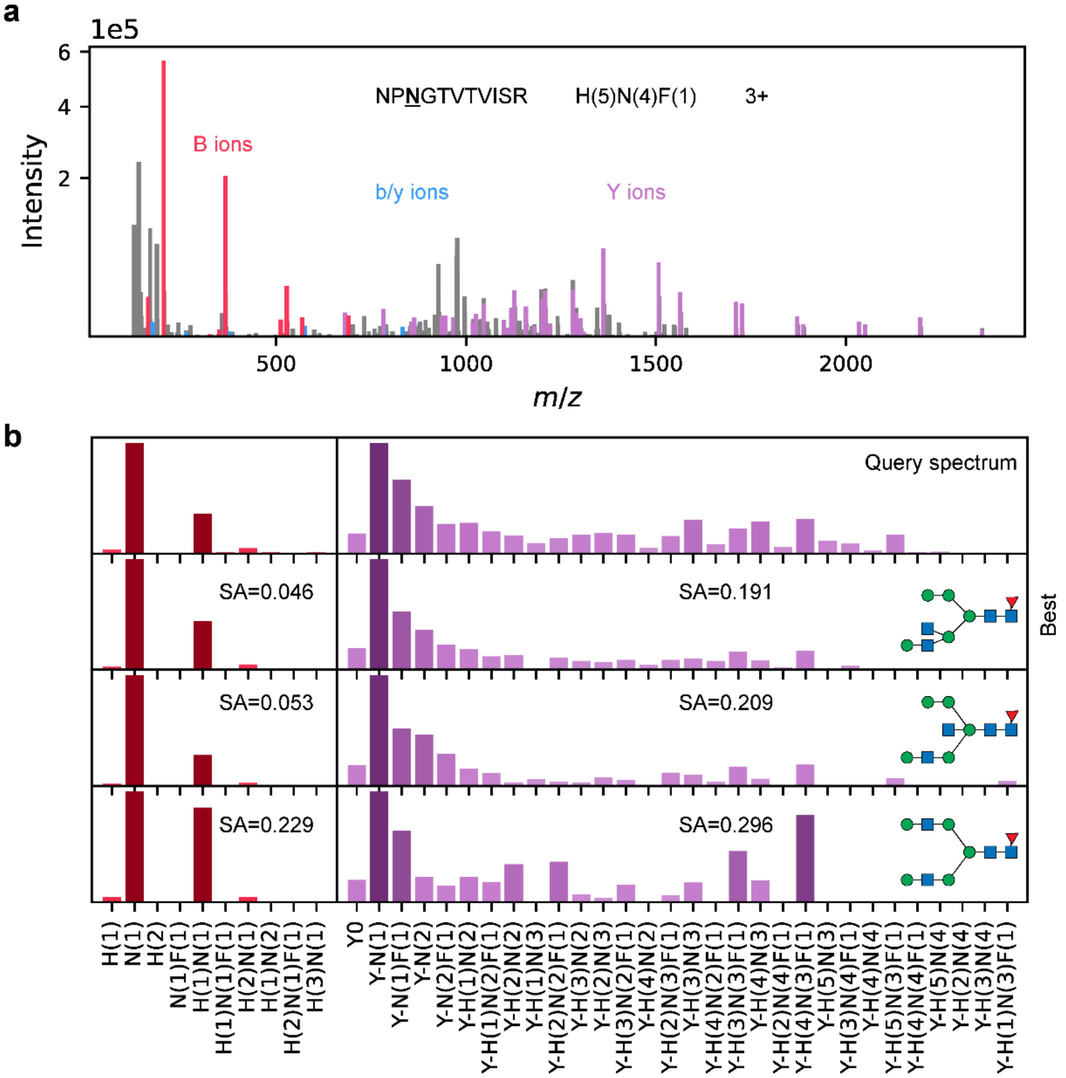

Supplementary Fig. 43. Example spectral match where spectral library searching confirms the branch terminal HexNAc in the original StrucGP annotations.

(**a**) The query spectrum. (**b**) Intensity patterns of the query spectrum and predicted spectra of the candidate glycan structures. Spectra angle loss (SA) values are indicated for each candidate. Other candidates are not shown.

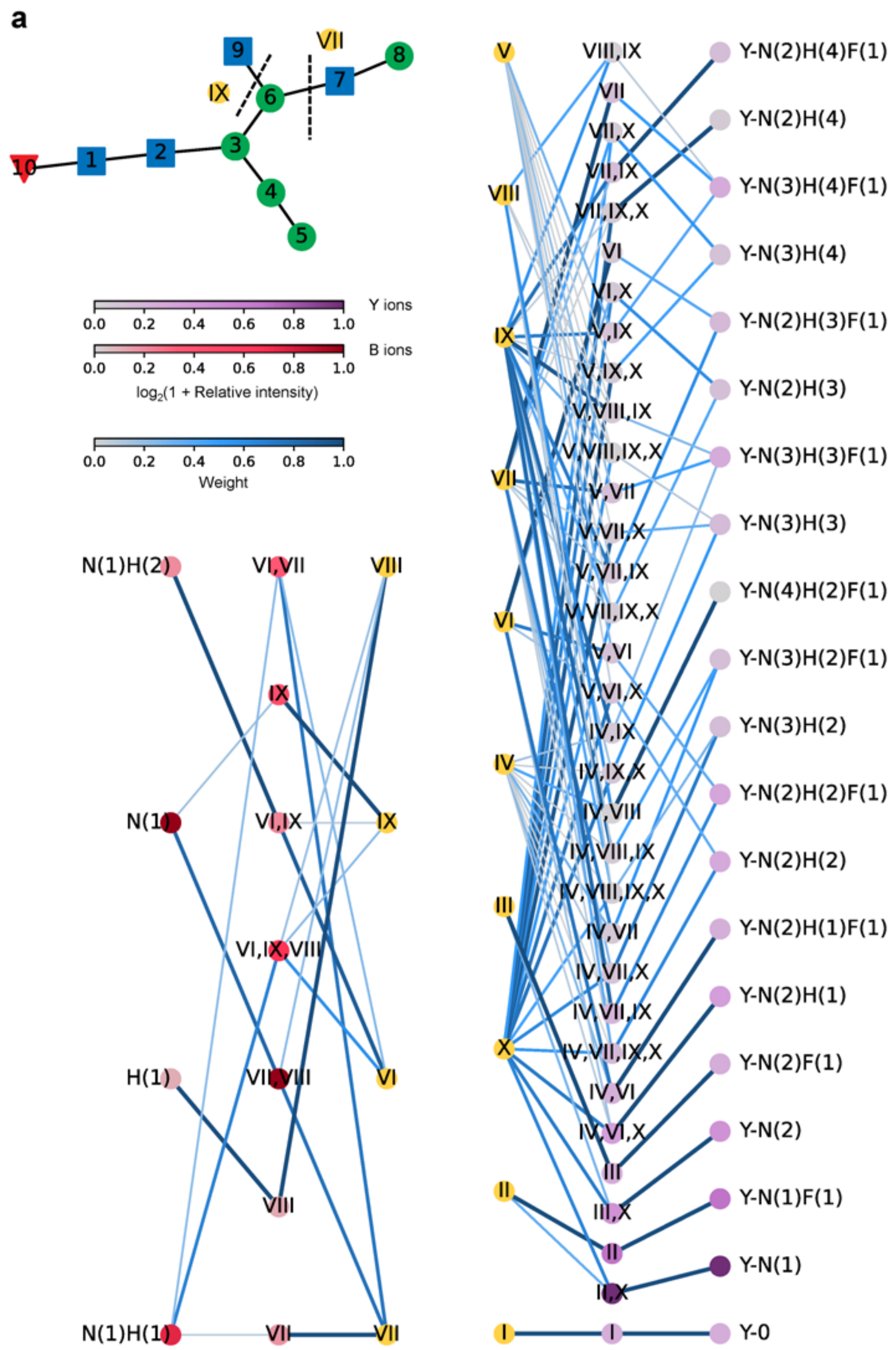

Supplementary Fig. 44. Glycan fragmentation graphs of example glycopeptides with a branch terminal HexNAc, with a bisecting HexNAc, and without any terminal HexNAc.

(**a**) The first candidate in **Supplementary Fig. 43** with a branch terminal HexNAc. (Continued on next page)

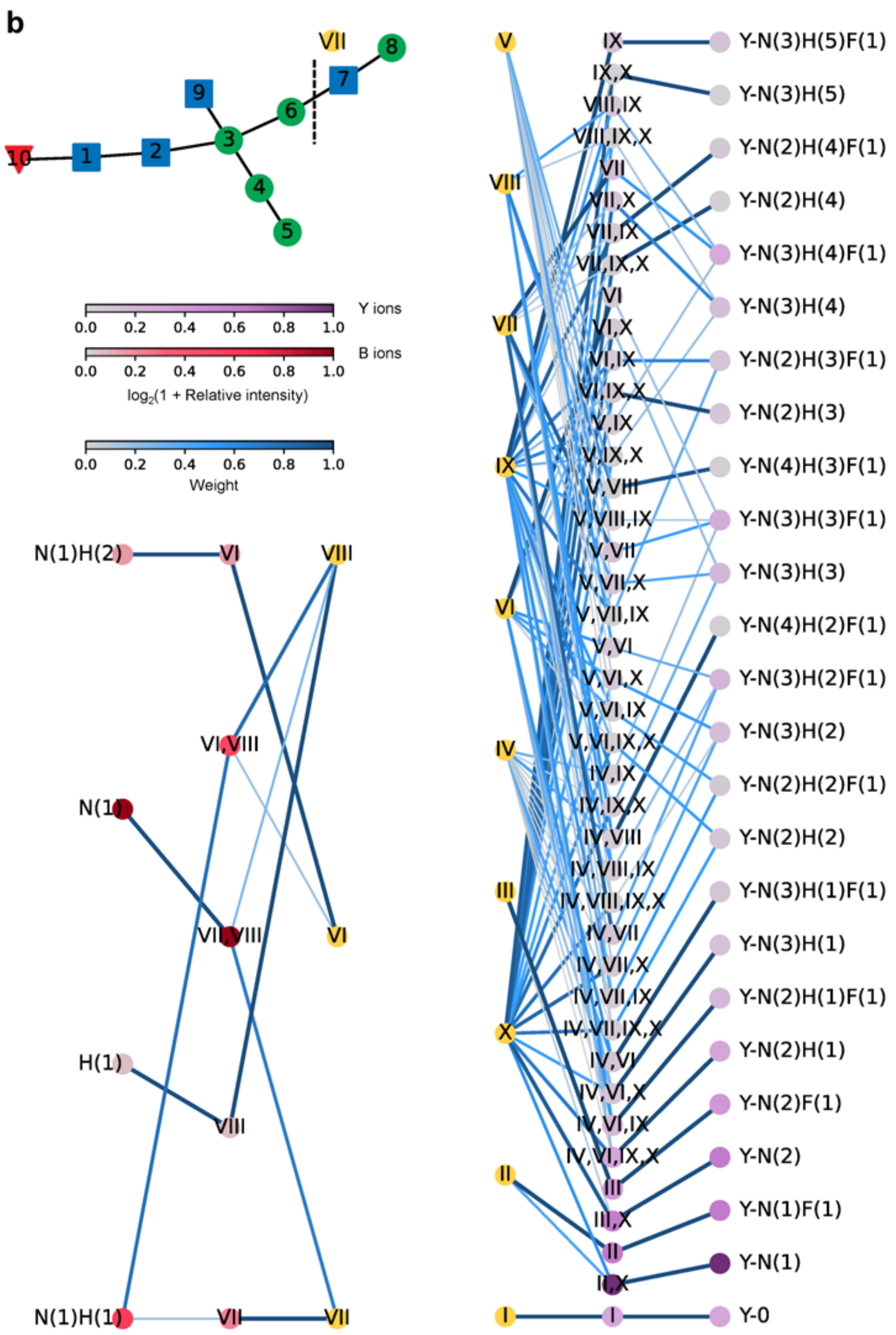

Supplementary Fig. 44 (Continued).

(**b**) The second candidate in **Supplementary Fig. 43** with a bisecting HexNAc. (Continued on next page)

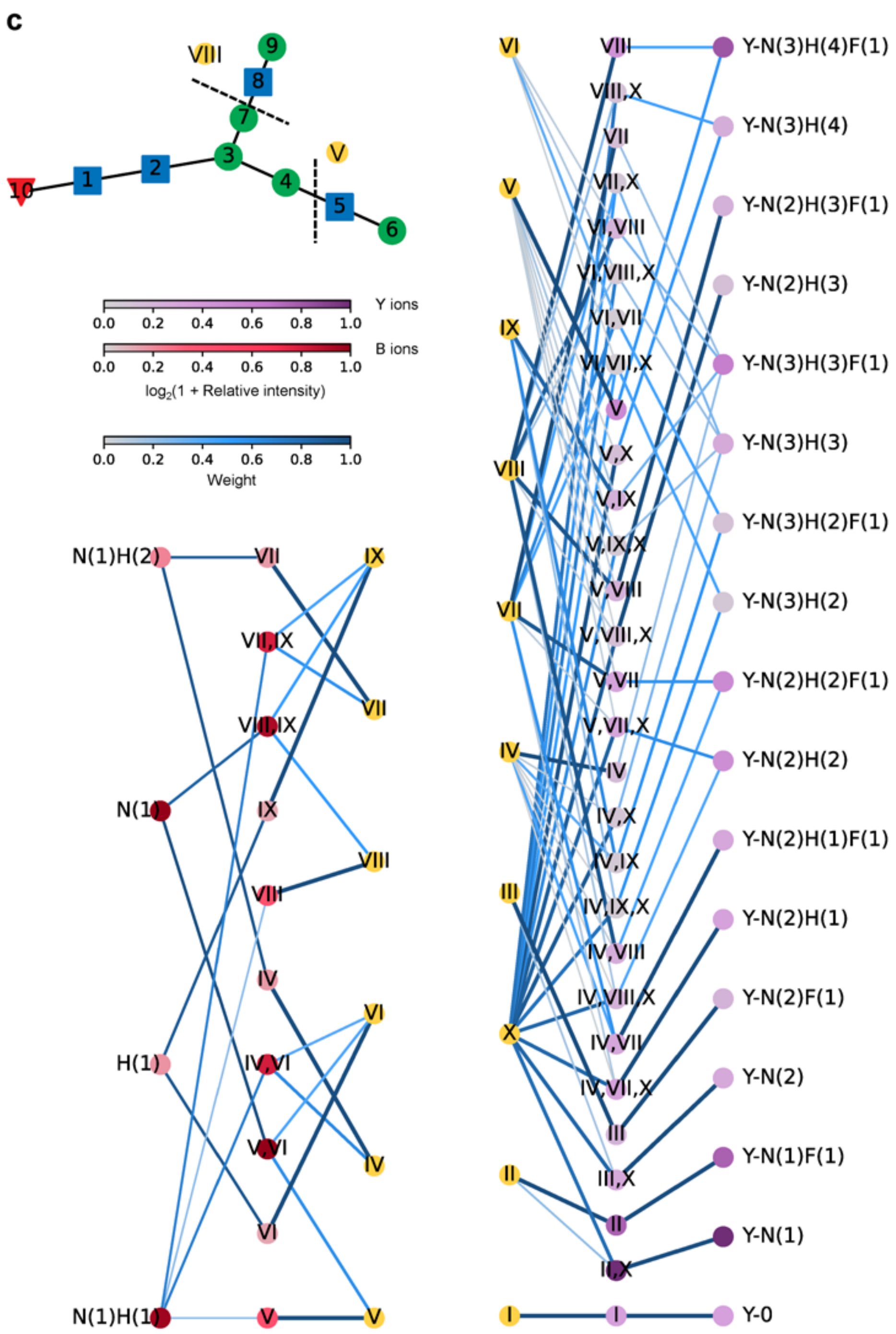

Supplementary Fig. 44 (Continued).

(**c**) The third candidate in **Supplementary Fig. 43** without any terminal HexNAc. The cleavage site (yellow nodes) at the non-reducing end of each monosaccharide is represented in Roman numerals. The color of structure-specific fragment nodes and composition-based fragment nodes indicated the relative intensity of B ions (red) or Y ions (purple). Nodes with relative intensity <0.01 are not shown. The color of edges linking cleavage nodes to structure-specific fragment nodes indicates the attention weights. The color of edges linking structure-specific fragment nodes to composition-based fragment nodes indicates the proportion of intensity that each isomeric fragment contributes to the MS peak.

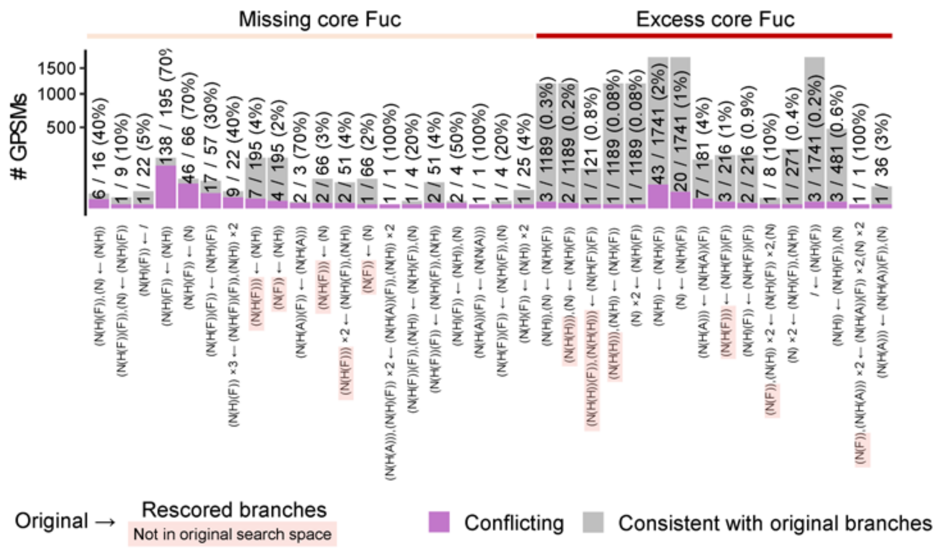

Supplementary Fig. 45. Conflicting results of core fucosylation recognition between spectral library searching with the original StrucGP annotations from the Fut8 knockout mouse brain dataset.

Results are categorized into types of conflictions (labels of x-axis), defined by glycan branches (in pGlyco format) of the original annotations and the top candidates rescored by spectral library searching. For each type of branch confliction, the percentage of GPSMs with conflicting results (purple) is calculated out of those that have a possibility of this type of confliction, i.e., all the GPSMs whose original annotation includes the original glycan branches (as well as core fucosylation and bisecting HexNAc if any) of the type of confliction (purple + grey).

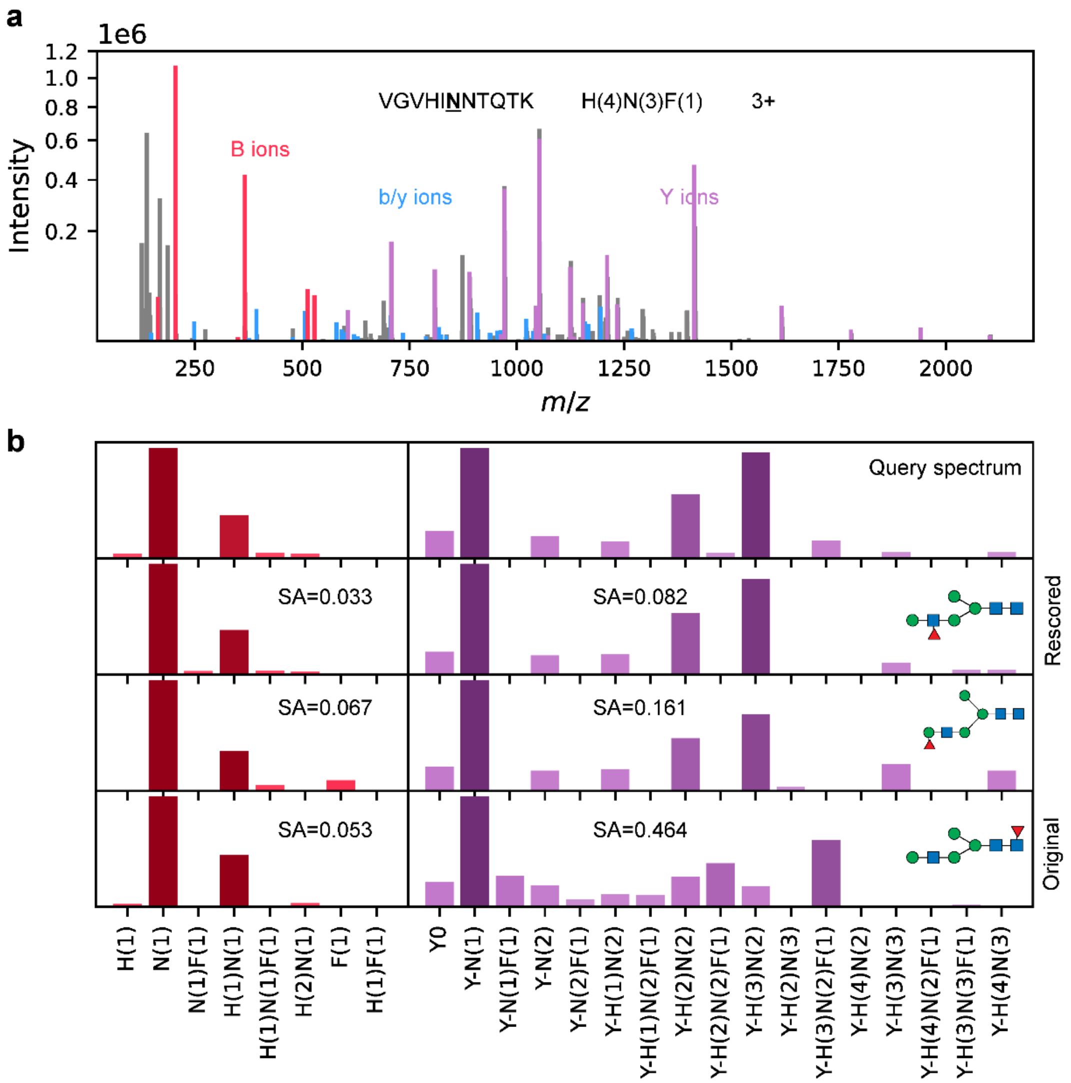

Supplementary Fig. 46. Example spectral match where spectral library searching dismisses the core fucosylation in the original StrucGP annotations.

(**a**) The query spectrum. (**b**) Intensity patterns of the query spectrum and predicted spectra of the candidate glycan structures. Spectra angle loss (SA) values are indicated for each candidate. Other candidates are not shown.

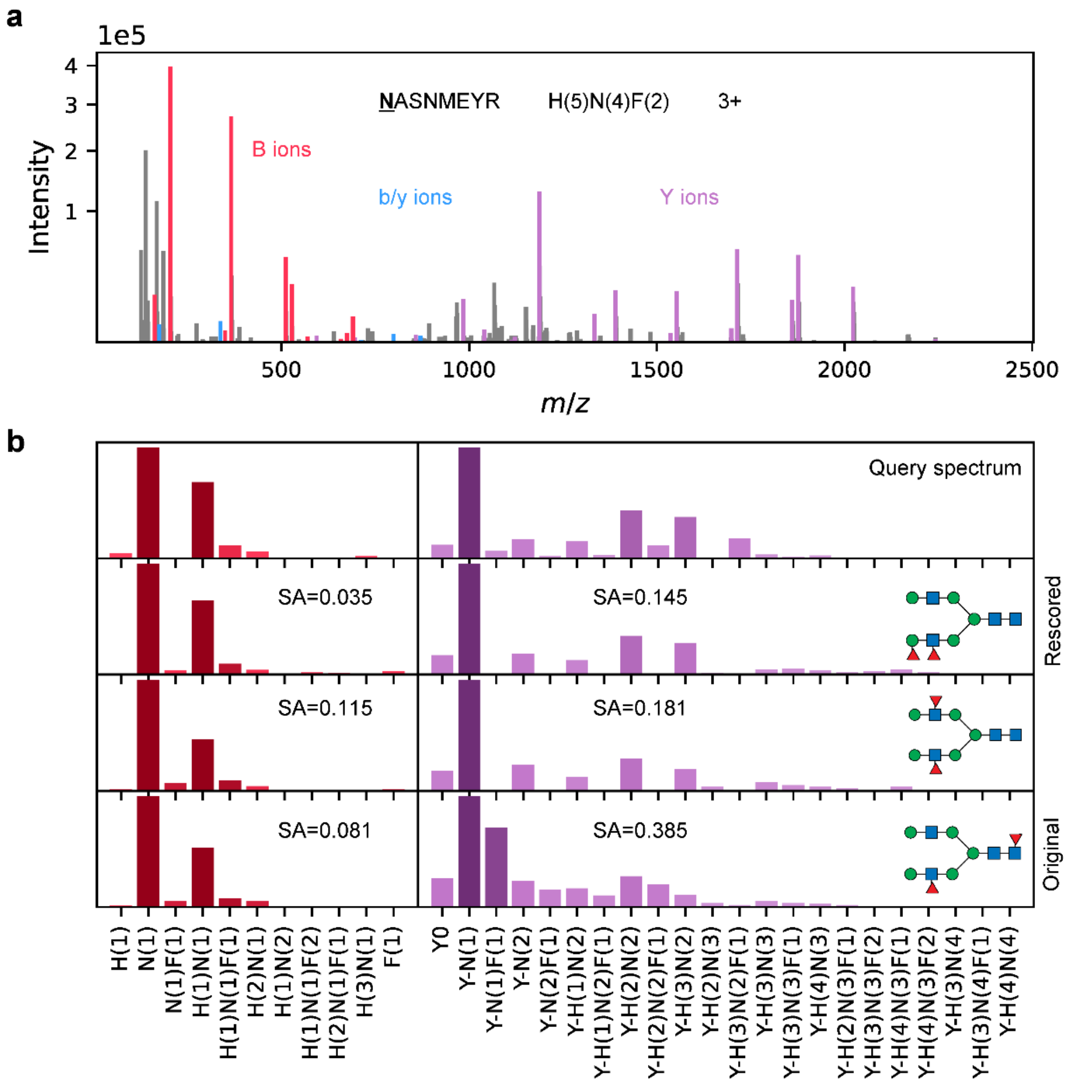

Supplementary Fig. 47. Example spectral match where spectral library searching dismisses the core fucosylation in the original StrucGP annotations.

(**a**) The query spectrum. (**b**) Intensity patterns of the query spectrum and predicted spectra of the candidate glycan structures. Spectra angle loss (SA) values are indicated for each candidate. Other candidates are not shown.

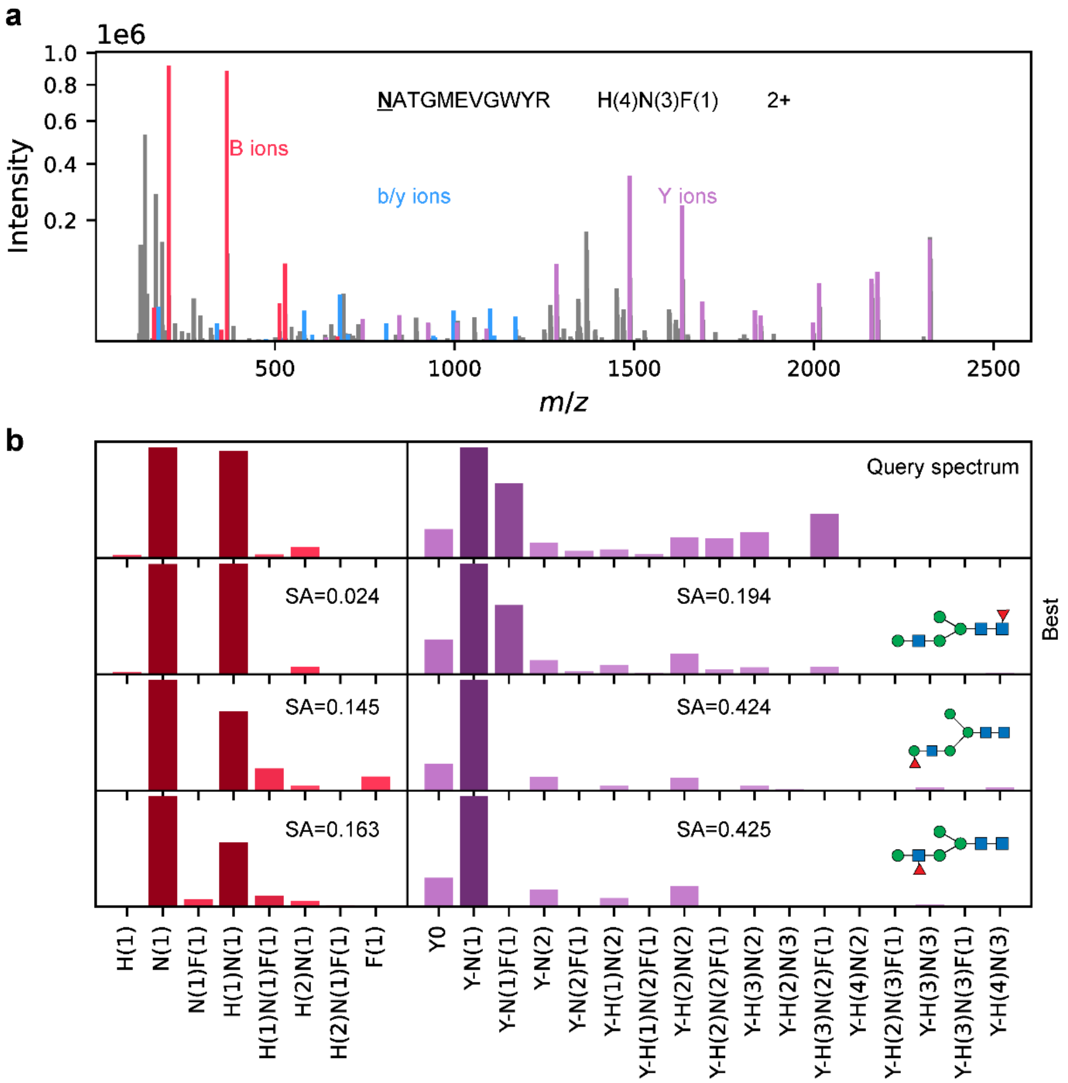

Supplementary Fig. 48. Example spectral match where spectral library searching confirms the core fucosylation in the original StrucGP annotations.

(**a**) The query spectrum. (**b**) Intensity patterns of the query spectrum and predicted spectra of the candidate glycan structures. Spectra angle loss (SA) values are indicated for each candidate. Other candidates are not shown.

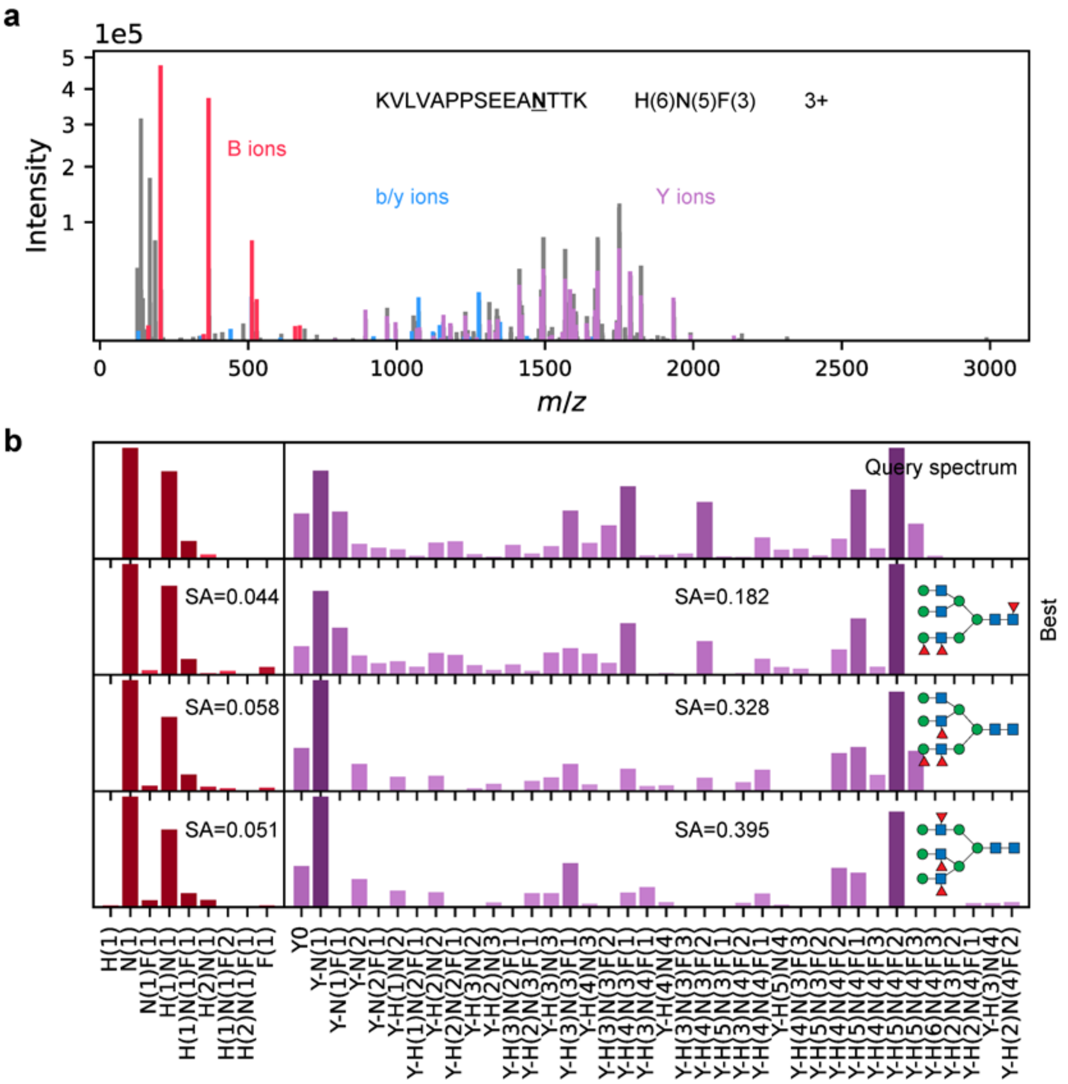

Supplementary Fig. 49. Example spectral match where spectral library searching confirms the core fucosylation in the original StrucGP annotations.

(**a**) The query spectrum. (**b**) Intensity patterns of the query spectrum and predicted spectra of the candidate glycan structures. Spectra angle loss (SA) values are indicated for each candidate. Other candidates are not shown.

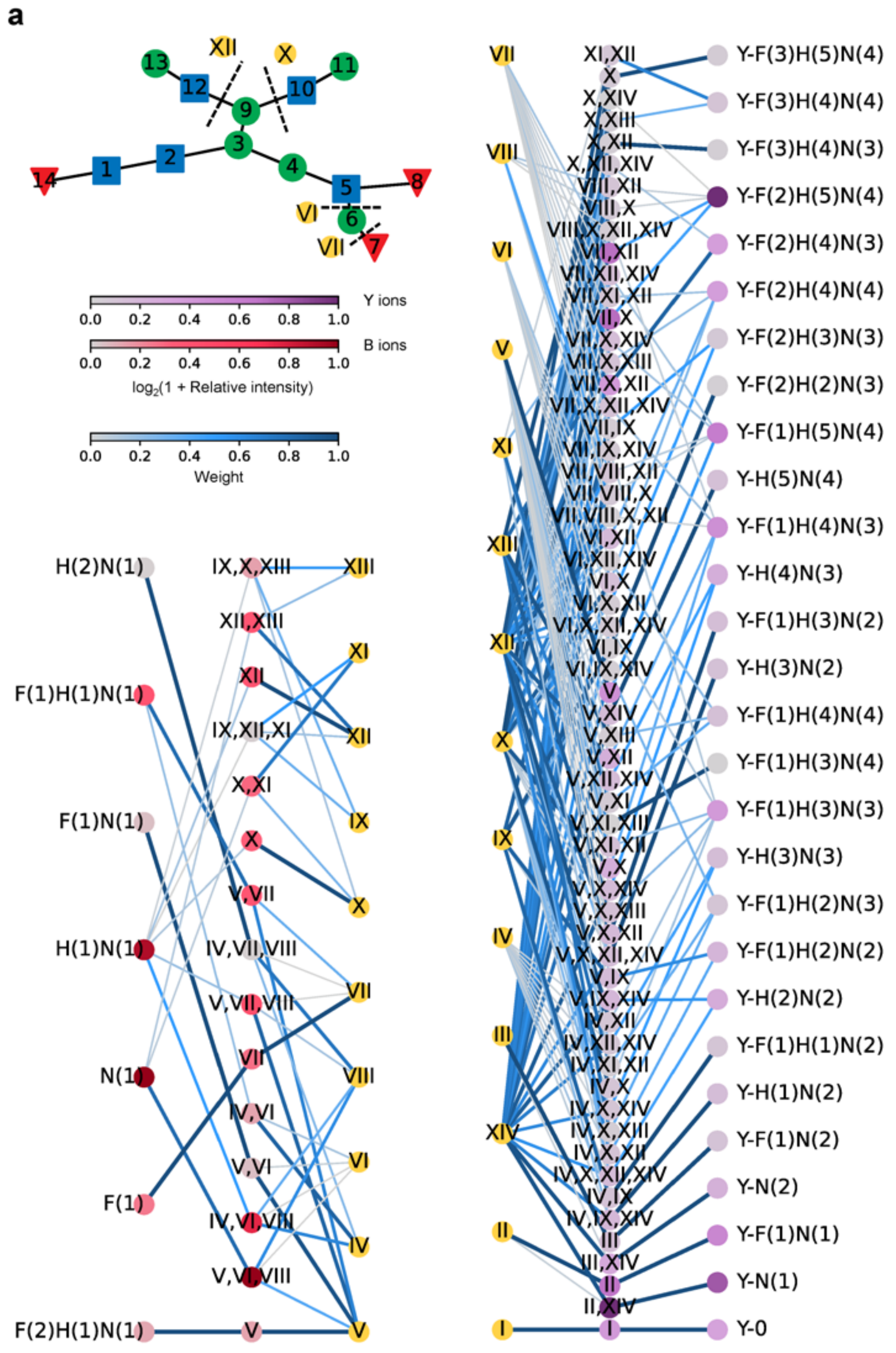

Supplementary Fig. 50. Glycan fragmentation graphs of example glycopeptides with and without core fucosylation.

(**a**) The first candidate in **Supplementary Fig. 49** with core fucosylation. (Continued on next page)

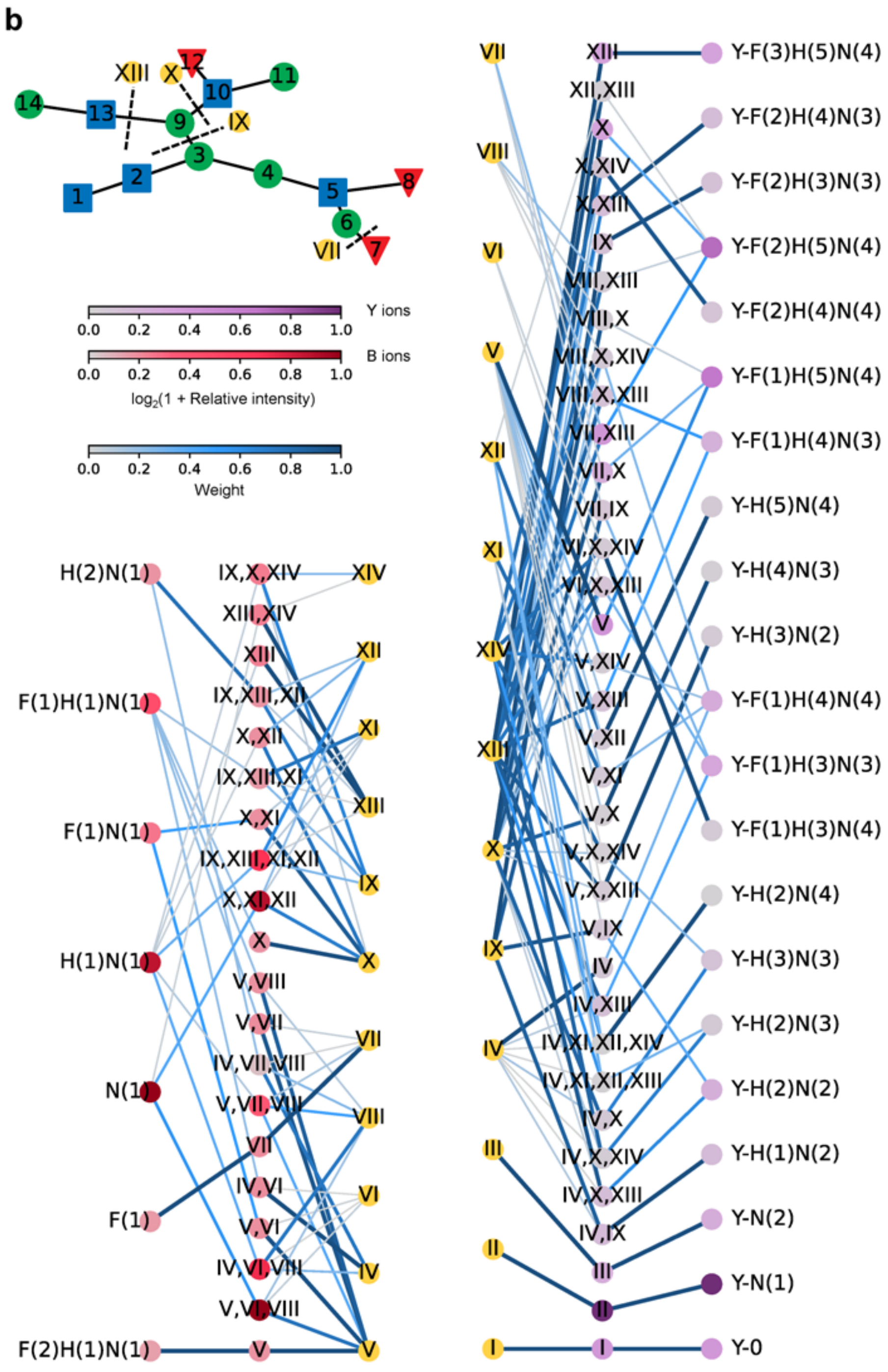

Supplementary Fig. 50 (Continued).

(**b**) The second candidate in **Supplementary Fig. 49** without core fucosylation. (Continued on next page)

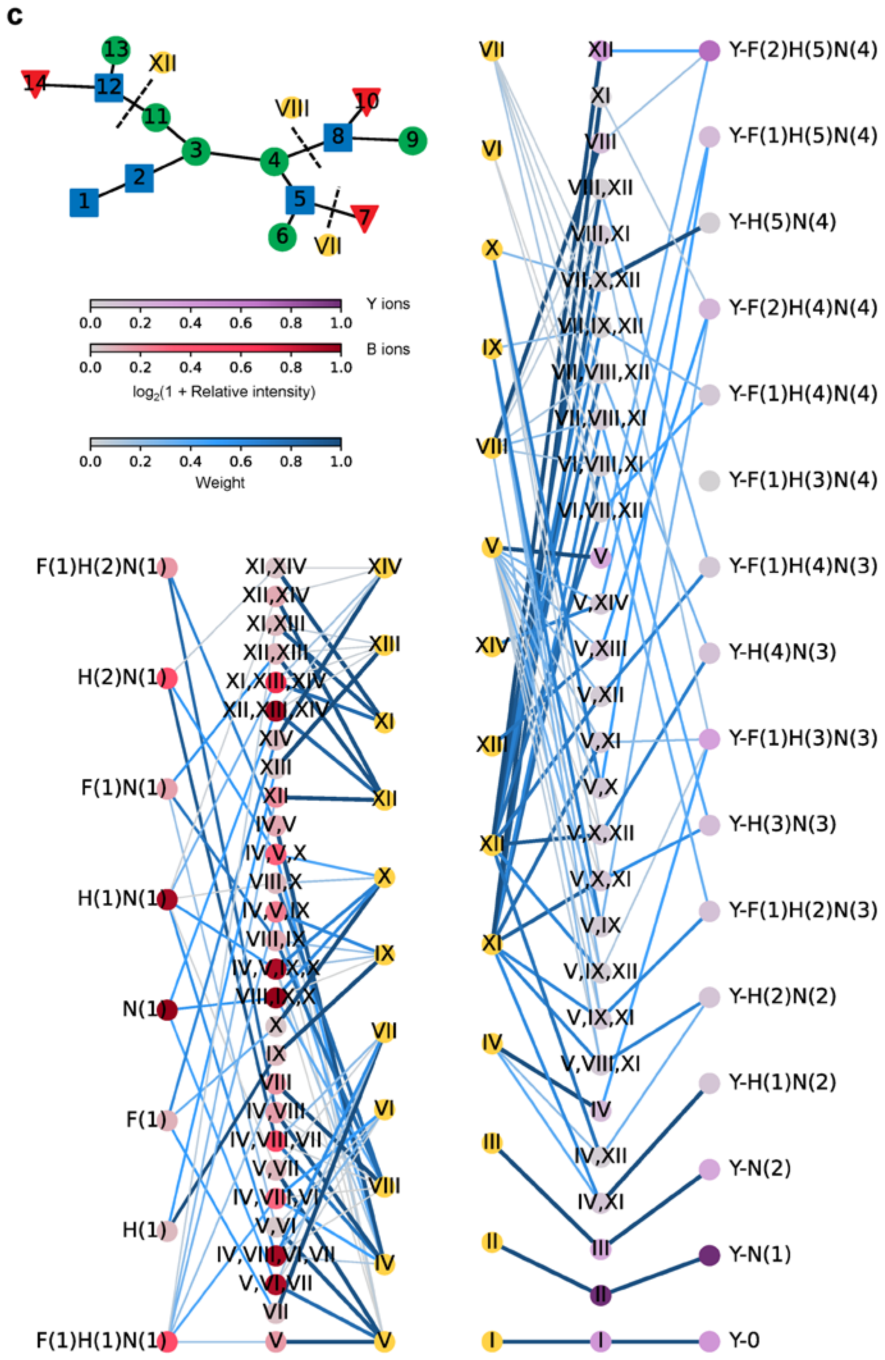

Supplementary Fig. 50 (Continued).

(**c**) The third candidate in **Supplementary Fig. 49** without core fucosylation. The cleavage site (yellow nodes) at the non-reducing end of each monosaccharide is represented in Roman numerals. The color of structure-specific fragment nodes and composition-based fragment nodes indicated the relative intensity of B ions (red) or Y ions (purple). Nodes with relative intensity <0.01 are not shown. The color of edges linking cleavage nodes to structure-specific fragment nodes indicates the attention weights. The color of edges linking structure-specific fragment nodes to composition-based fragment nodes indicates the proportion of intensity that each isomeric fragment contributes to the MS peak.

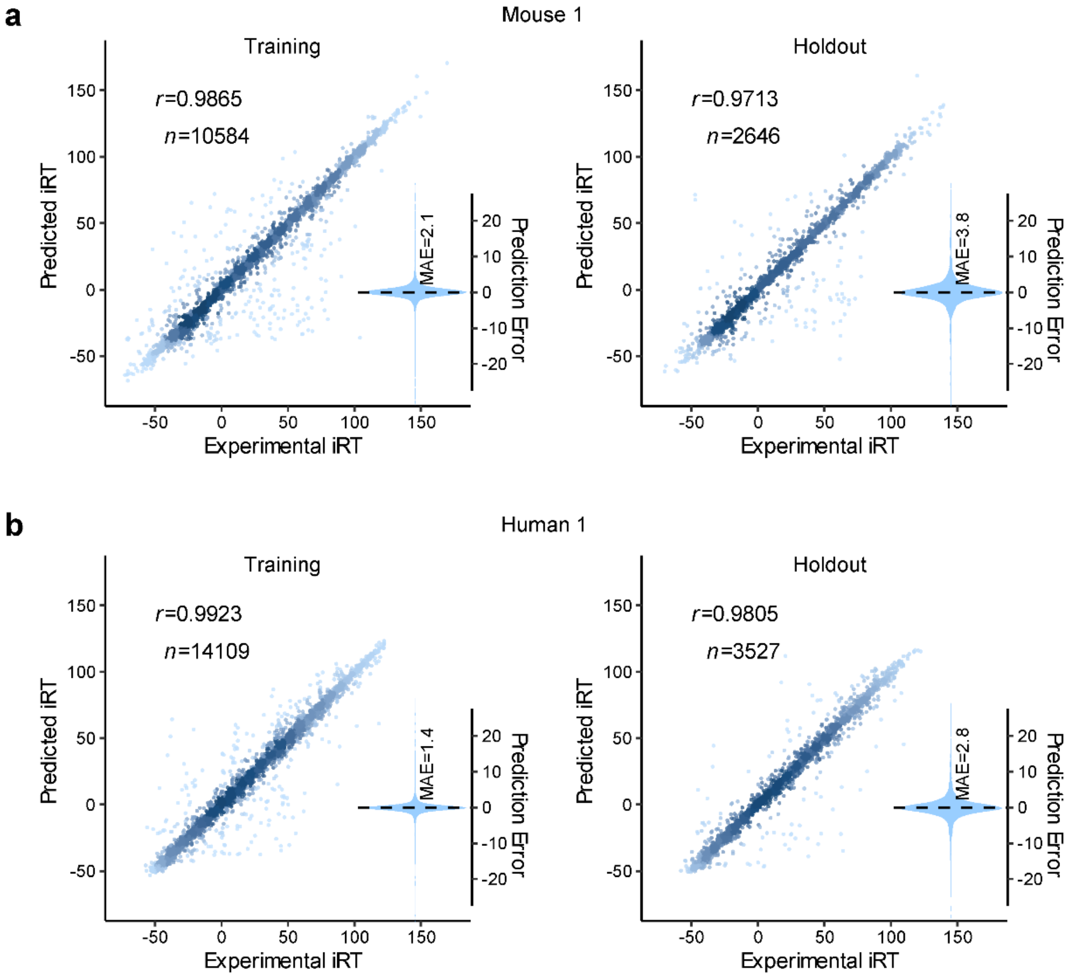

Supplementary Fig. 51. Performance of glycopeptide retention time prediction.

(**a**) Correlations of normalized retention time (iRT) between predicted and experimental values using a model trained and tested on the Mouse1 dataset. (**b**) Results tested on Human1 datasets. The Pearson correlation coefficients (*r*), as well as data size (*n*), are indicated for the training set and holdout set. The right inset shows distributions of prediction error, with mean absolute error (MAE) indicated. Source data are provided as a Source Data file.

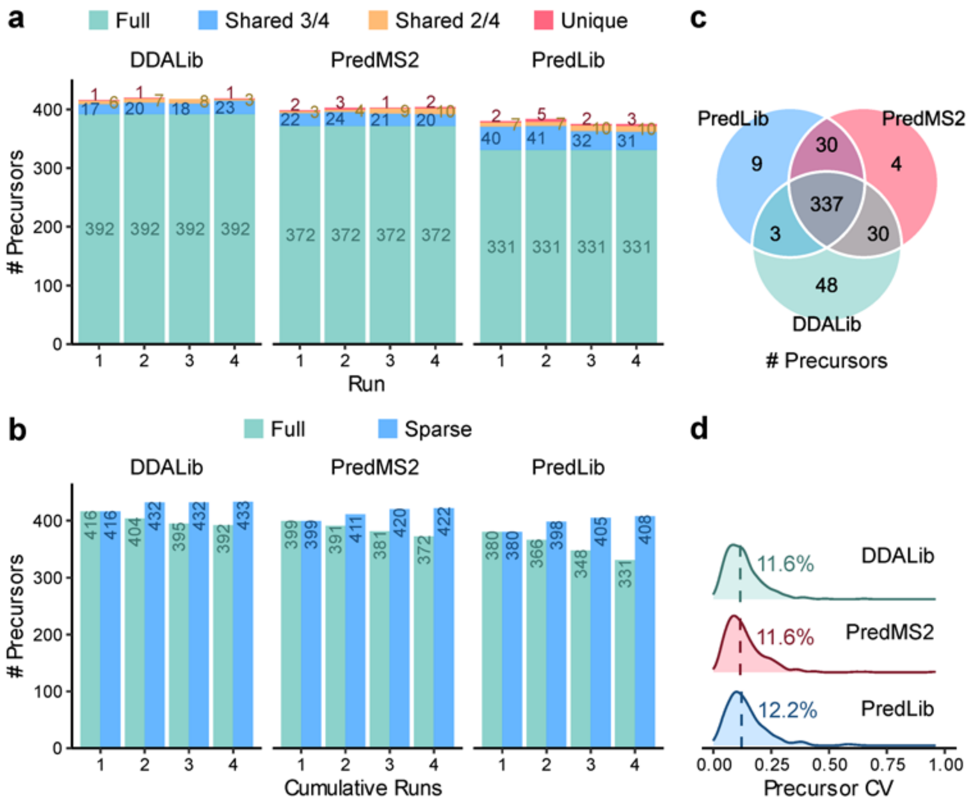

Supplementary Fig. 52. DIA results of the fission yeast sample at the precursor level using the experimental (DDALib) and predicted spectral libraries (PredMS2 and PredLib).

(**a**) Numbers of identifications per run. “Full” represents identifications observed in all the runs; “shared 3/4” and “shared 2/4” represent identifications observed in 3 and 2 runs, respectively; “unique” represents identifications observed in only 1 run. (**b**) Numbers of cumulative identifications from run 1 to 4. “Full” represents identifications shared in the cumulative runs; “sparse” represents identifications observed in at least one run in the cumulative runs. (**c**) Comparison of numbers of identifications shared in >50% runs. (**d**) Coefficient of variation (CV) values of quantification. Medians are indicated. Results of DDALib are from the original publication of GproDIA. Source data are provided as a Source Data file.

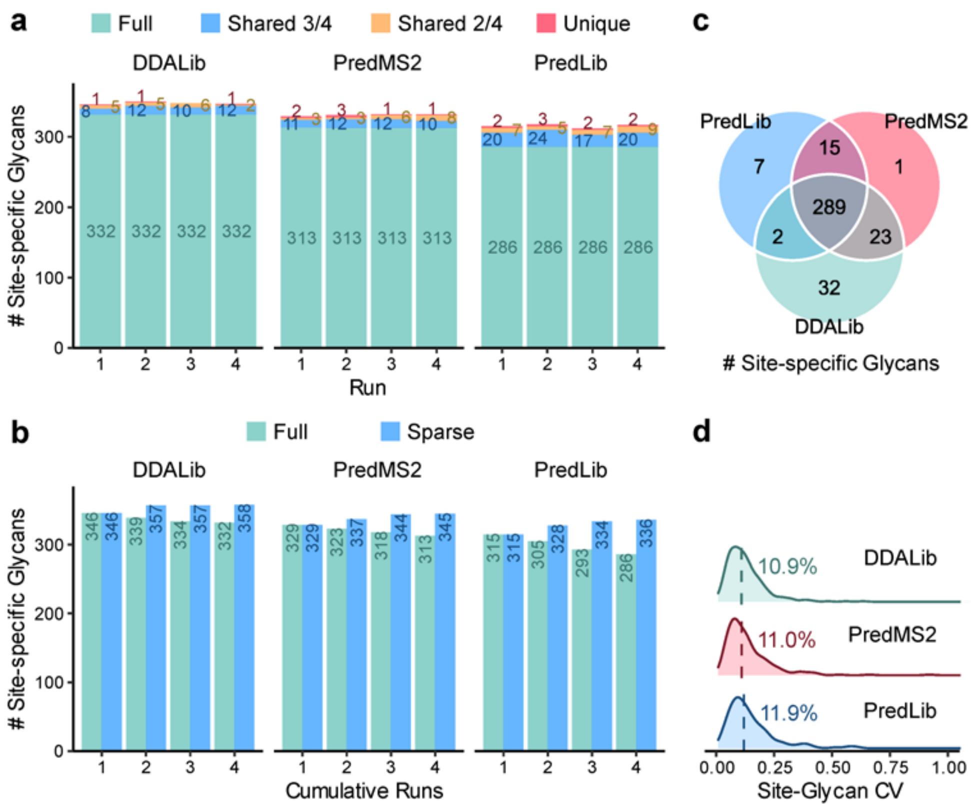

Supplementary Fig. 53. DIA results of the fission yeast sample at the site-specific glycan level using the experimental (DDALib) and predicted spectral libraries (PredMS2 and PredLib).

(**a**) Numbers of identifications per run. “Full” represents identifications observed in all the runs; “shared 3/4” and “shared 2/4” represent identifications observed in 3 and 2 runs, respectively; “unique” represents identifications observed in only 1 run. (**b**) Numbers of cumulative identifications from run 1 to 4. “Full” represents identifications shared in the cumulative runs; “sparse” represents identifications observed in at least one run in the cumulative runs. (**c**) Comparison of numbers of identifications shared in >50% runs. (**d**) Coefficient of variation (CV) values of quantification. Medians are indicated. Results of DDALib are from the original publication of GproDIA. Source data are provided as a Source Data file.

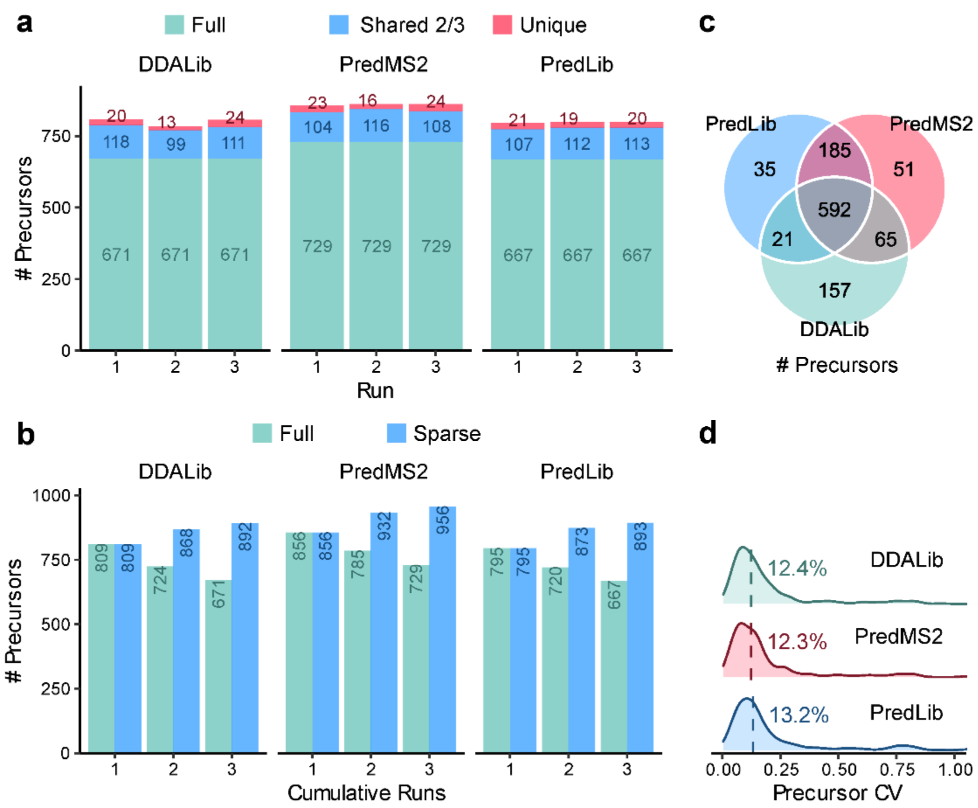

Supplementary Fig. 54. DIA results of the human serum sample at the precursor level using the experimental (DDALib) and predicted spectral libraries (PredMS2 and PredLib).

(**a**) Numbers of identifications per run. “Full” represents identifications observed in all the runs; “shared 2/3” represents identifications observed in 2 runs; “unique” represents identifications observed in only 1 run. (**b**) Numbers of cumulative identifications from run 1 to 3. “Full” represents identifications shared in the cumulative runs; “sparse” represents identifications observed in at least one run in the cumulative runs. (**c**) Comparison of numbers of identifications shared in >50% runs. (**d**) Coefficient of variation (CV) values of quantification. Medians are indicated. Results of DDALib are from the original publication of GproDIA. Source data are provided as a Source Data file.

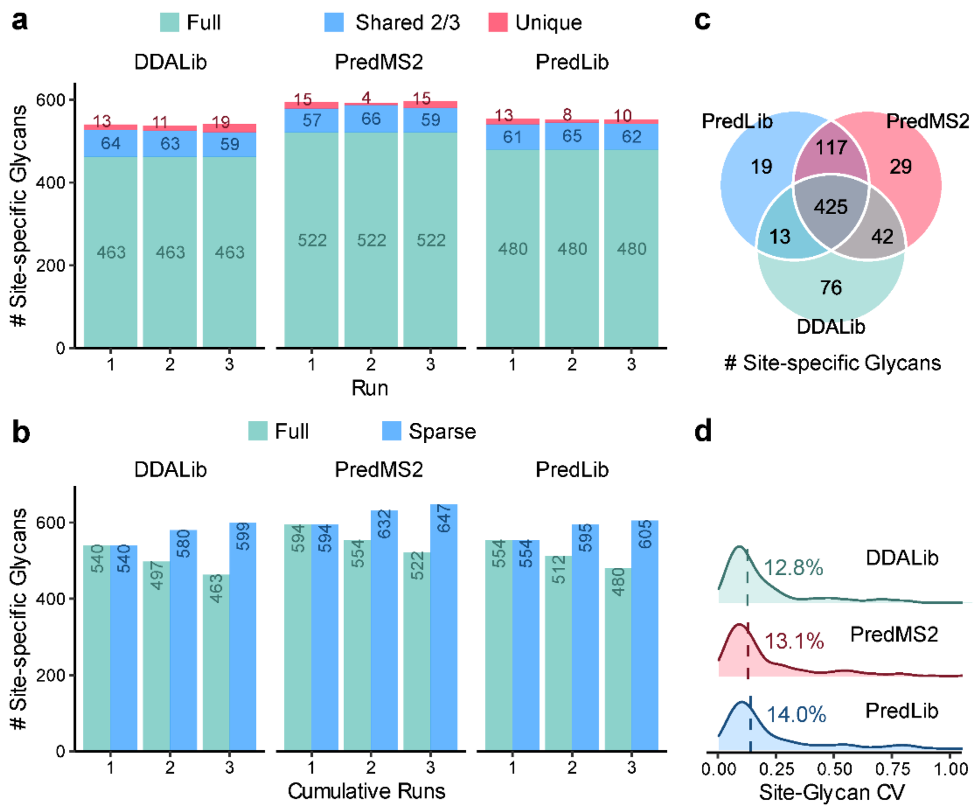

Supplementary Fig. 55. DIA results of the human serum sample at the site-specific glycan level using the experimental (DDALib) and predicted spectral libraries (PredMS2 and PredLib).

(**a**) Numbers of identifications per run. “Full” represents identifications observed in all the runs; “shared 2/3” represents identifications observed in 2 runs; “unique” represents identifications observed in only 1 run. (**b**) Numbers of cumulative identifications from run 1 to 4. “Full” represents identifications shared in the cumulative runs; “sparse” represents identifications observed in at least one run in the cumulative runs. (**c**) Comparison of numbers of identifications shared in >50% runs. (**d**) Coefficient of variation (CV) values of quantification. Medians are indicated. Results of DDALib are from the original publication of GproDIA. Source data are provided as a Source Data file.

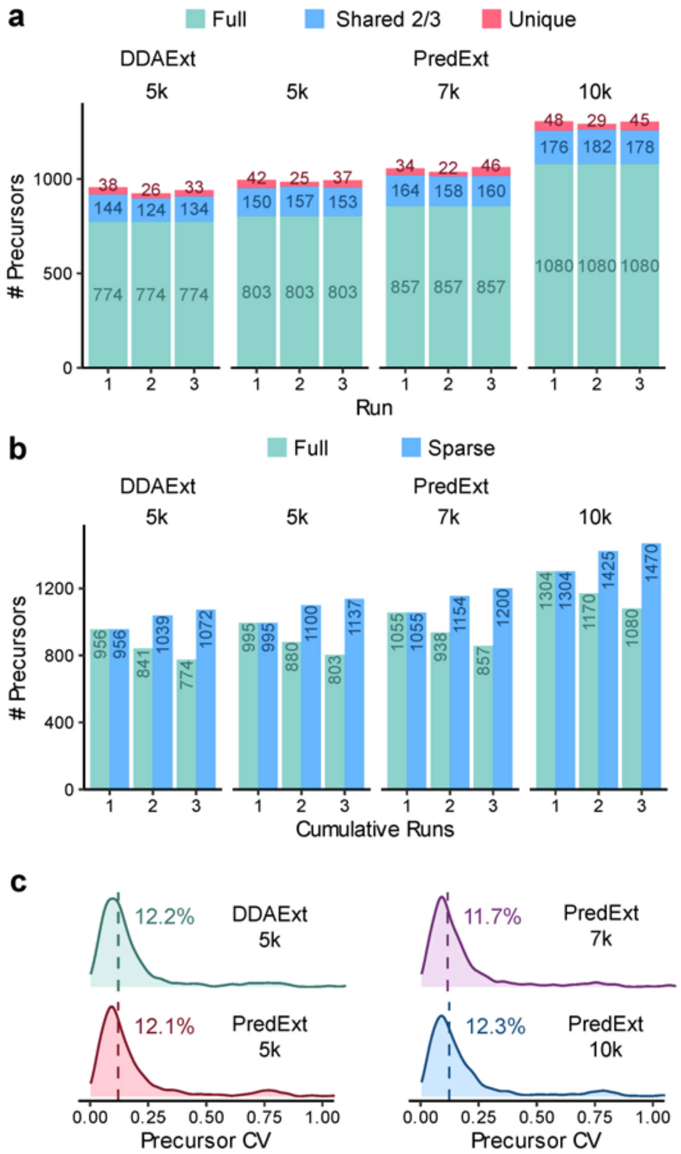

Supplementary Fig. 56. DIA results of the human serum sample at the precursor level using the semi-empirical spectral library by GproDIA (DDAExt) and the extended predicted spectral libraries with increasing coverage (PredExt 5k, 7k, and 10k).

(**a**) Numbers of identifications per run. "Full" represents identifications observed in all the runs; "shared 2/3" represents identifications observed in 2 runs; "unique" represents identifications observed in only 1 run. (**b**) Numbers of cumulative identifications from run 1 to 3. "Full" represents identifications shared in the cumulative runs; "sparse" represents identifications observed in at least one run in the cumulative runs. (**c**) Coefficient of variation (CV) values of quantification. Medians are indicated. Source data are provided as a Source Data file.

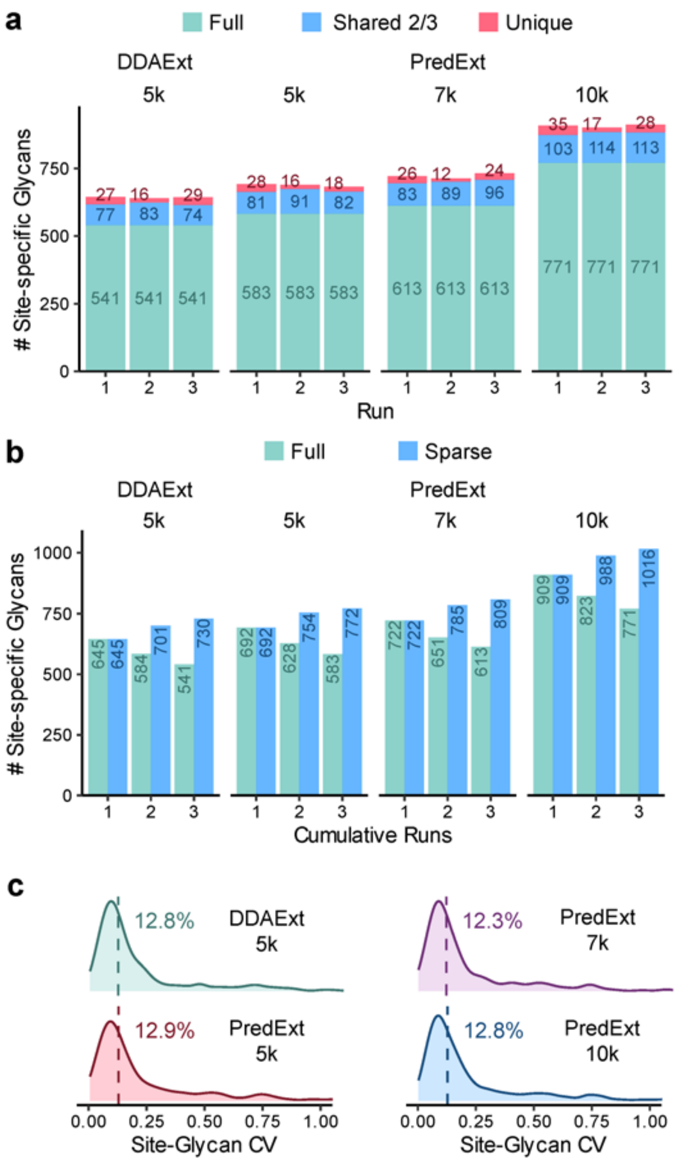

Supplementary Fig. 57. DIA results of the human serum sample at the site-specific glycan level using the semi-empirical spectral library by GproDIA (DDAExt) and the extended predicted spectral libraries with increasing coverage (PredExt 5k, 7k, and 10k).

(**a**) Numbers of identifications per run. "Full" represents identifications observed in all the runs; "shared 2/3" represents identifications observed in 2 runs; "unique" represents identifications observed in only 1 run. (**b**) Numbers of cumulative identifications from run 1 to 3. "Full" represents identifications shared in the cumulative runs; "sparse" represents identifications observed in at least one run in the cumulative runs. (**c**) Coefficient of variation (CV) values of quantification. Medians are indicated. Source data are provided as a Source Data file.

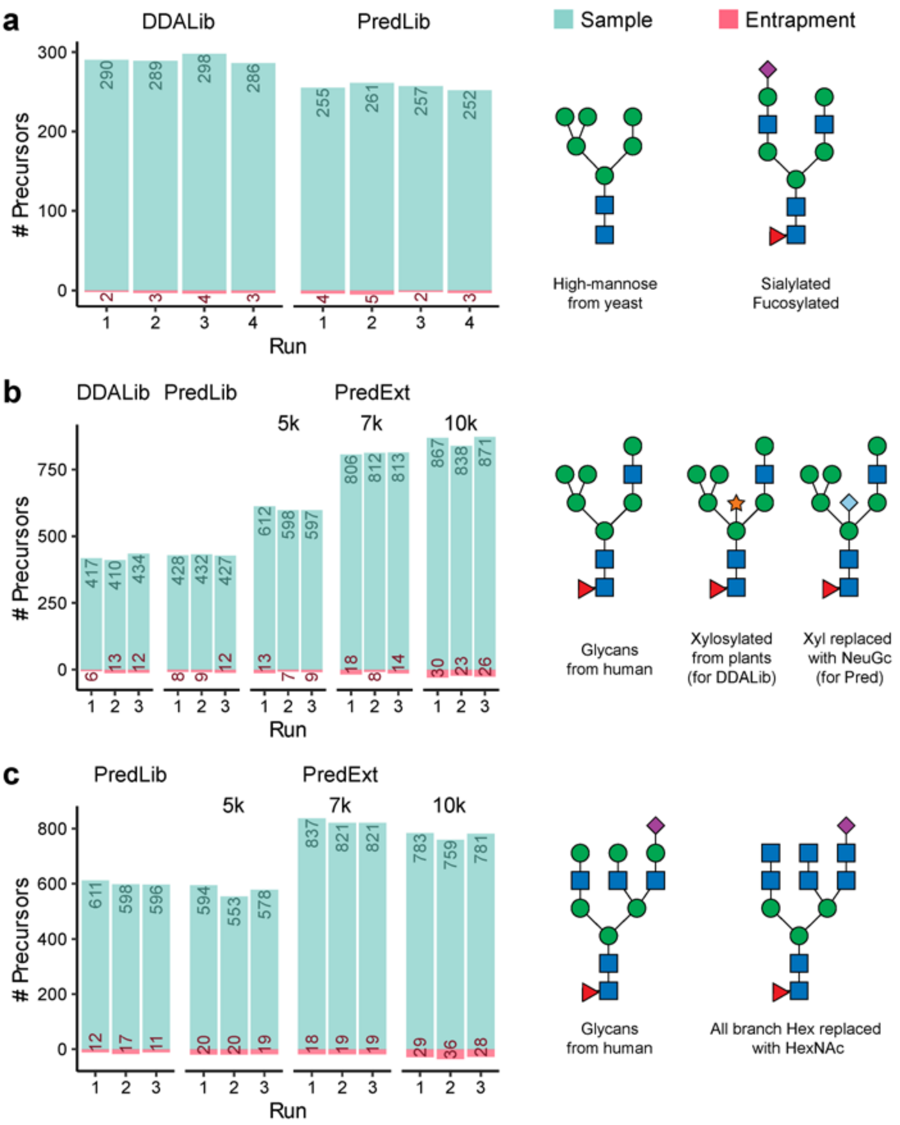

Supplementary Fig. 58. DIA results of using the entrapment libraries.

(**a**) Numbers of identifications from the fission yeast sample using the glycan entrapment libraries (containing glycopeptides with peptide sequences from yeast and glycans from human). (**b**) Results from the human serum sample using the monosaccharide-based entrapments. (**c**) Results from the human serum sample using the composition-based entrapments. The entrapment libraries are generated based on the experimental (DDALib) and the predicted spectral libraries (PredLib and PredExt), where library entries with glycans not present in the sample are append to the original libraries with a size ratio of approximately 1:1. Results of DDALib are from the original publication of GproDIA.

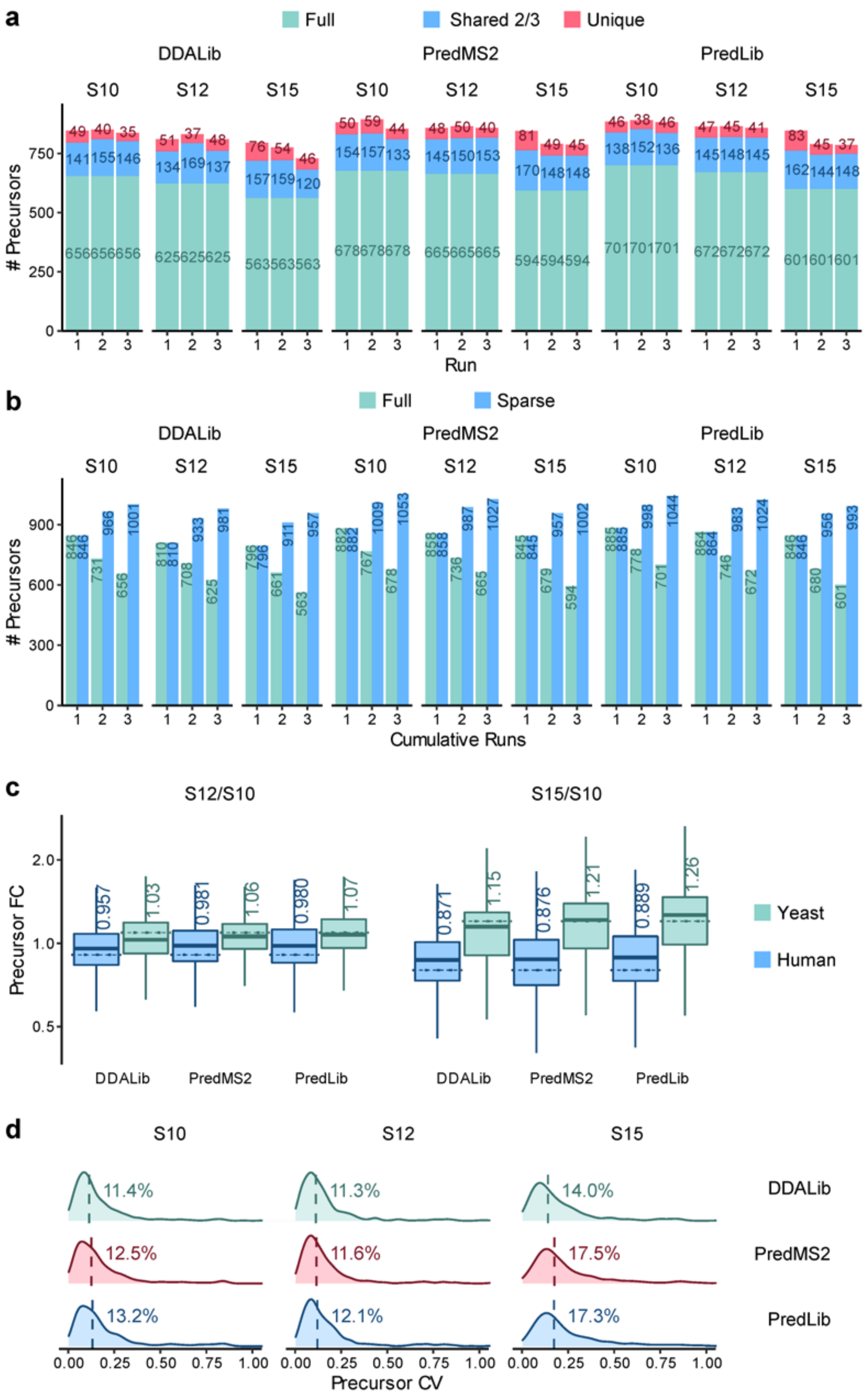

a
Full
Shared 2/3
Unique
DDALib
PredMS2
PredLib
S10
S12
S15
Precursors
Run
b
Full
Sparse
Cumulative Runs
c
S12/S10
S15/S10
Precursor FC
Yeast
Human
d
Precursor CV

Supplementary Fig. 59. DIA results of the mixed-organism samples at the precursor level using the experimental (DDALib) and predicted spectral libraries (PredMS2 and PredLib).

(**a**) Numbers of identifications per run of each sample. “Full” represents identifications observed in all the runs; “shared 2/3” represents identifications observed in 2 runs; “unique” represents identifications observed in only 1 run. (**b**) Numbers of cumulative identifications from run 1 to 3 of each sample. “Full” represents identifications shared in the cumulative runs; “sparse” represents identifications observed in at least one run in the cumulative runs. (**c**) Box plot visualization of fold change (FC) values of the quantification results of the mixed-organism samples. Percent changes are calculated based on the mean quantities in three replicates of each sample. The medians are indicated. The boxes indicate the interquartile ranges (IQR), and whiskers indicate $1.5 \times$ IQR values; no outliers are shown. The dashed lines indicate theoretical fold changes of the organisms (S10:S12:S15 = 1:0.9:0.8 for human and 1:1.1:1.2 for yeast). (**d**) Coefficient of variation (CV) values of quantification. Medians are indicated. Results of DDALib are from the original publication of GproDIA. Source data are provided as a Source Data file.

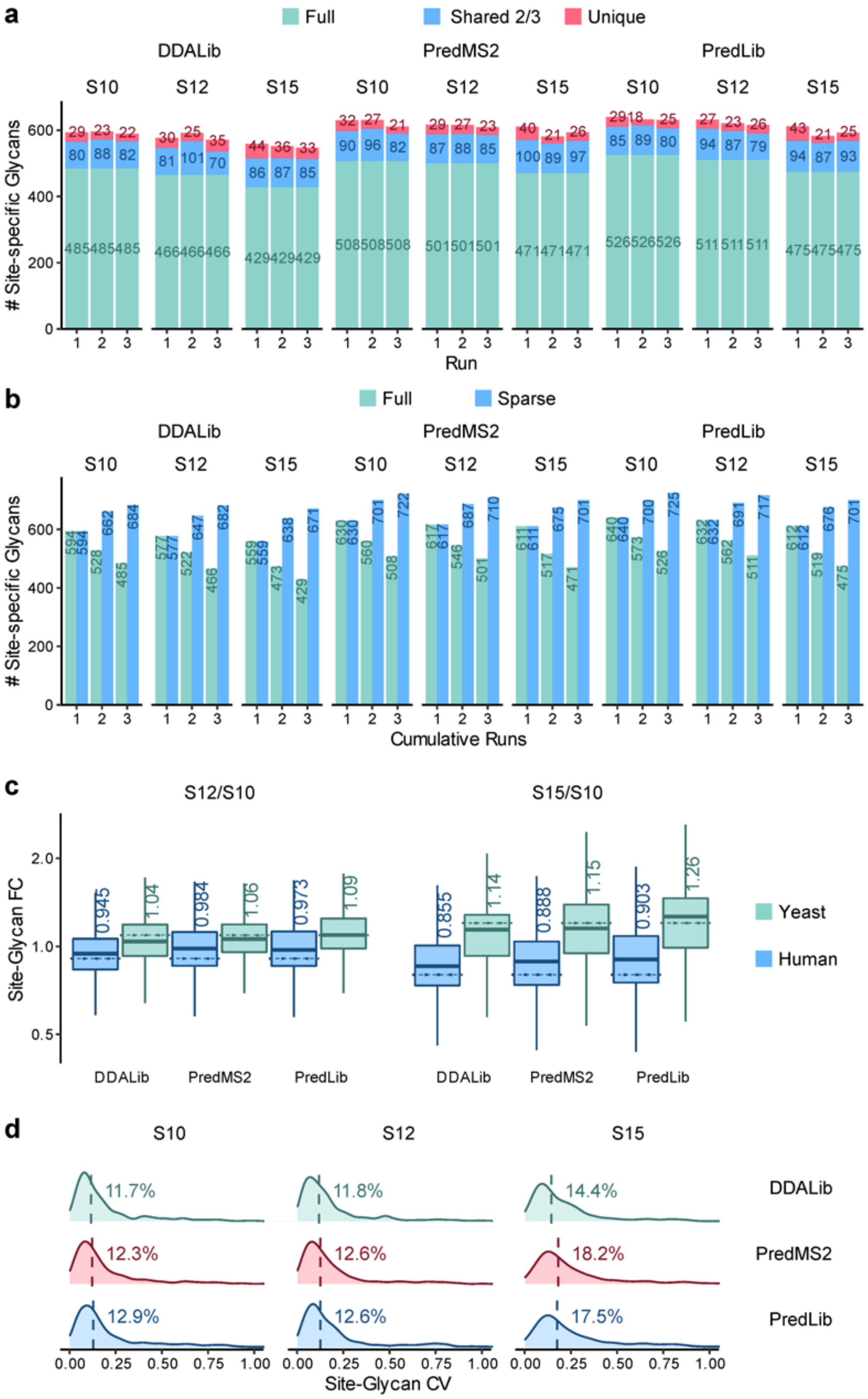
a
Full
Shared 2/3
Unique
DDALib
PredMS2
PredLib
S10
S12
S15
Site-specific Glycans
0
200
400
600
29 23 22
80 88 82
485 485 485
30 25 35
81 101 70
466 466 466
44 36 33
86 87 85
429 429 429
32 27 21
90 96 82
508 508 508
29 27 23
87 88 85
501 501 501
40 21 26
100 89 97
471 471 471
29 18 25
85 89 80
526 526 526
27 23 26
94 87 79
511 511 511
43 21 25
94 87 93
475 475 475
1 2 3
Run
b
Full
Sparse
594 594 528 662 485 684
577 577 522 647 466 682
559 559 473 638 429 671
630 630 560 701 508 722
617 617 546 687 501 710
611 611 517 675 471 701
640 640 573 700 526 725
632 632 562 691 511 717
612 612 519 676 475 701
Cumulative Runs
c
S12/S10
S15/S10
Site-Glycan FC
0.5
1.0
2.0
0.945
1.04
0.984
1.06
0.973
1.09
0.855
1.14
0.888
1.15
0.903
1.26
DDALib
PredMS2
PredLib
Yeast
Human
d
S10
S12
S15
11.7%
11.8%
14.4%
12.3%
12.6%
18.2%
12.9%
12.6%
17.5%
DDALib
PredMS2
PredLib
0.00 0.25 0.50 0.75 1.00
Site-Glycan CV

Supplementary Fig. 60. DIA results of the mixed-organism samples at the site-specific glycan level using the experimental (DDALib) and predicted spectral libraries (PredMS2 and PredLib).

(**a**) Numbers of identifications per run of each sample. “Full” represents identifications observed in all the runs; “shared 2/3” represents identifications observed in 2 runs; “unique” represents identifications observed in only 1 run. (**b**) Numbers of cumulative identifications from run 1 to 3 of each sample. “Full” represents identifications shared in the cumulative runs; “sparse” represents identifications observed in at least one run in the cumulative runs. (**c**) Box plot visualization of fold change (FC) values of the quantification results of the mixed-organism samples. Percent changes are calculated based on the mean quantities in three replicates of each sample. The medians are indicated. The boxes indicate the interquartile ranges (IQR), and whiskers indicate $1.5 \times$ IQR values; no outliers are shown. The dashed lines indicate theoretical fold changes of the organisms (S10:S12:S15 = 1:0.9:0.8 for human and 1:1.1:1.2 for yeast). (**d**) Coefficient of variation (CV) values of quantification. Medians are indicated. Results of DDALib are from the original publication of GproDIA. Source data are provided as a Source Data file.

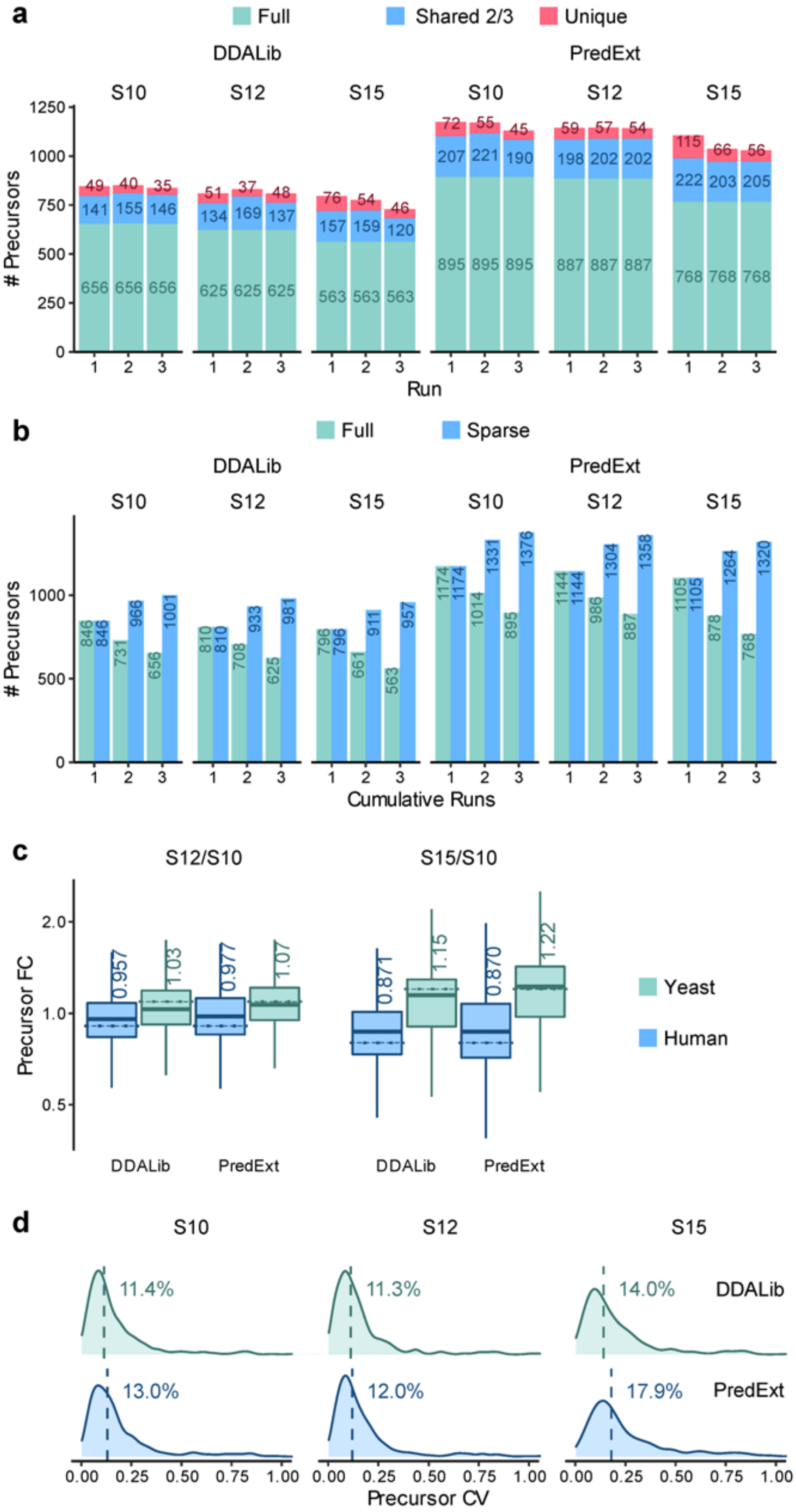
a
Full
Shared 2/3
Unique
DDALib
PredExt
S10
S12
S15
Precursors
Run
b
Full
Sparse
Cumulative Runs
c
S12/S10
S15/S10
Precursor FC
DDALib
PredExt
Yeast
Human
d
DDALib
PredExt
Precursor CV

Supplementary Fig. 61. DIA results of the mixed-organism samples at the precursor level using the experimental spectral library (DDALib) and the extended predicted spectral library (PredExt).

(**a**) Numbers of identifications per run of each sample. “Full” represents identifications observed in all the runs; “shared 2/3” represents identifications observed in 2 runs; “unique” represents identifications observed in only 1 run. (**b**) Numbers of cumulative identifications from run 1 to 3 of each sample. “Full” represents identifications shared in the cumulative runs; “sparse” represents identifications observed in at least one run in the cumulative runs. (**c**) Box plot visualization of fold change (FC) values of the quantification results of the mixed-organism samples. Percent changes are calculated based on the mean quantities in three replicates of each sample. The medians are indicated. The boxes indicate the interquartile ranges (IQR), and whiskers indicate $1.5 \times$ IQR values; no outliers are shown. The dashed lines indicate theoretical fold changes of the organisms (S10:S12:S15 = 1:0.9:0.8 for human and 1:1.1:1.2 for yeast). (**d**) Coefficient of variation (CV) values of quantification. Medians are indicated. Results of DDALib are from the original publication of GproDIA. Source data are provided as a Source Data file.

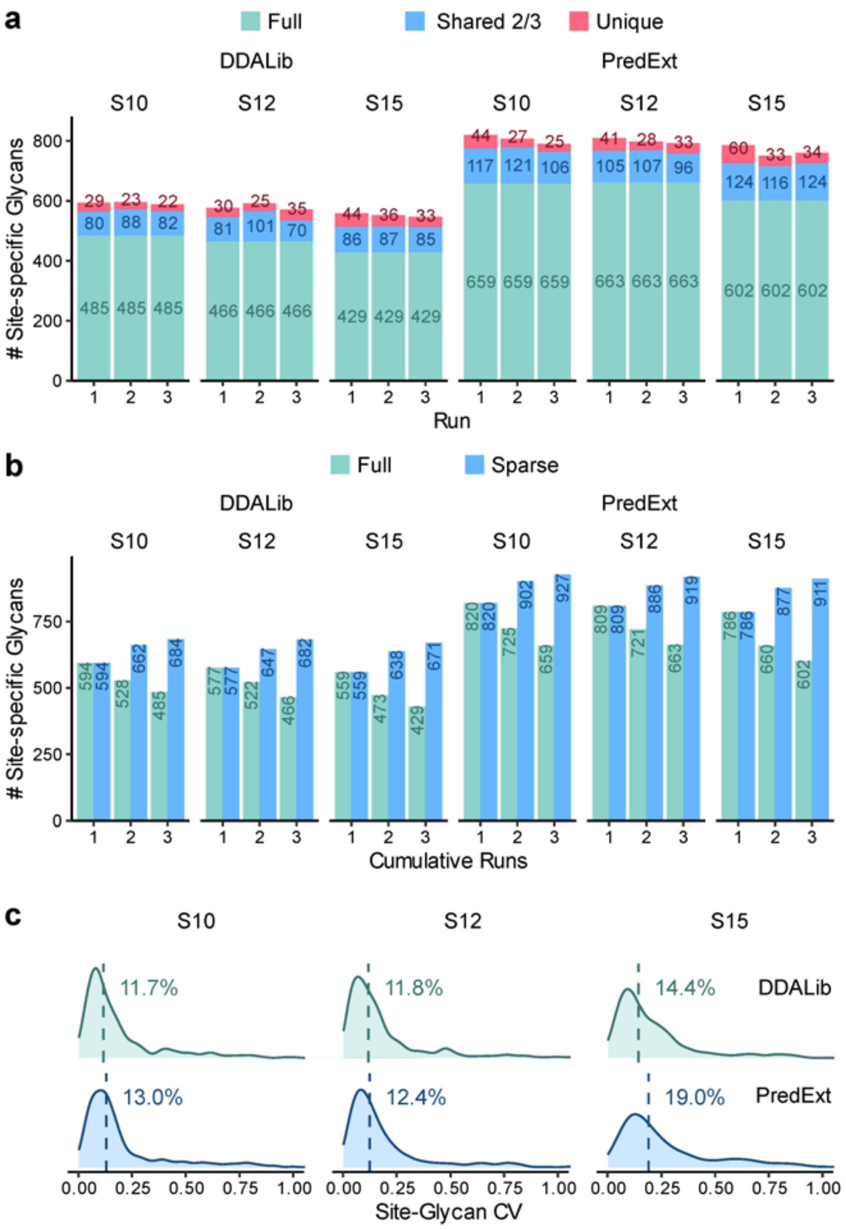

Supplementary Fig. 62. DIA results of the mixed-organism samples at the site-specific glycan level using the experimental spectral library (DDALib) and the extended predicted spectral library (PredExt).

(**a**) Numbers of identifications per run of each sample. “Full” represents identifications observed in all the runs; “shared 2/3” represents identifications observed in 2 runs; “unique” represents identifications observed in only 1 run. (**b**) Numbers of cumulative identifications from run 1 to 3 of each sample. “Full” represents identifications shared in the cumulative runs; “sparse” represents identifications observed in at least one run in the cumulative runs. (**c**) Coefficient of variation (CV) values of quantification. Medians are indicated. Results of DDALib are from the original publication of GproDIA. Source data are provided as a Source Data file.

Supplementary Table 1. Nomenclature of monosaccharides used in this study.

Char	Symbol	Description
H	green circle	Hexose (Hex), including mannose (Man), glucose (Glc), and galactose (Gal)
N	blue square	*N*-Acetylhexosamine (HexNAc), including *N*-acetylglucosamine (GlcNAc) and *N*-acetylgalactosamine (GalNAc)
A	purple diamond	*N*-Acetylneuraminic acid (NeuAc)
G	light blue diamond	*N*-Glycolylneuraminic acid (NeuGc)
F	red triangle	Fucose (Fuc)

The nomenclature of monosaccharides is defined by pGlyco and slightly different from the standard Symbol Nomenclature for Glycans (SNFG). Monosaccharides with the same mass are in the same color.

Supplementary Table 2. MS/MS datasets for model training and validation.

Name	Instrument	Description	Accession
Mouse1	Orbitrap Fusion<br>Stepped CE: 30±10	Mouse tissues, including the brain, heart, kidney, liver, and lung.<br>Raw data contains 60 runs, with 81173 GPSMs identified (after removing those with more than 2 Fuc), finally combined into consensus spectra of 12818 precursors of 10949 glycopeptides. Among them, 6202 precursors of 5492 glycopeptides are not of high-mannose type.	PXD005411,<br>PXD005413,<br>PXD005412,<br>PXD005553,<br>PXD005555
Mouse2	Orbitrap Fusion Lumos<br>Dual CEs: 20 & 33	Mouse brains.<br>Raw data contains 24 runs, with 80695 GPSMs identified, finally combined into consensus spectra of 5942 precursors of 5112 glycopeptides. Among them, 2739 precursors of 2402 glycopeptides are not of high-mannose type.	PXD025859
Human1	Orbitrap Fusion Lumos<br>Dual CEs: 20 & 33	Human spermatozoa and seminal plasma.<br>Raw data contains 73 runs, with 160965 GPSMs identified, finally combined into consensus spectra of 19317 precursors of 16163 glycopeptides. Among them, 15042 precursors of 12456 glycopeptides are not of high-mannose type.	PXD026649,<br>PXD030804
Human2	Orbitrap Exploris 480<br>Stepped CE: 30±10	Human serum.<br>Raw data contains 6 runs, with 22789 GPSMs identified, finally combined into consensus spectra of 3364 precursors of 2823 glycopeptides. Among them, 3052 precursors of 2553 glycopeptides are not of high-mannose type.	PXD031025

Stepped CE: HCD MS/MS is performed with multiple CEs and the data are recorded in a single spectrum for each precursor. Dual CEs: oxonium ions triggered data-dependent HCD MS/MS is performed, where data of different CEs are recorded in separate spectra; the isolation width can be different for each CE; the low and high CE spectra of the same precursor are merged in our data preprocessing procedure.

Supplementary Table 3. MS/MS datasets for model validation.

Name	Instrument	Description	Accession
Mouse3	Orbitrap Fusion Lumos<br>Dual CEs: 20 & 33	Mouse brains of wild type and FUT8 knockout.<br>Raw data contains 6 runs, with 23907 GPSMs identified, finally combined into consensus spectra of 5206 precursors of 4615 glycopeptides. Among them, 3806 precursors of 3129 glycopeptides are not of high-mannose type.	PXD035158
Mouse4	Orbitrap Fusion<br>Stepped CE: 30±10	Mouse peritoneal macrophages.<br>Raw data contains 27 runs, with 161277 GPSMs identified, finally combined into consensus spectra of 8512 precursors of 6763 glycopeptides. Among them, 2099 precursors of 1909 glycopeptides are not of high-mannose type.	PXD026629
Human3	Orbitrap Fusion Lumos<br>Dual CEs: 20 & 33	Human serum.<br>Raw data contains 6 runs, with 28210 GPSMs identified, finally combined into consensus spectra of 2487 precursors of 1972 glycopeptides. Among them, 2253 precursors of 1782 glycopeptides are not of high-mannose type.	PXD035158
Human4	Orbitrap Fusion<br>Stepped CE: 30±10	Human serum.<br>Raw data contains 21 runs, with 11069 GPSMs identified, finally combined into consensus spectra of 2576 precursors of 1988 glycopeptides. Among them, 2091 precursors of 1610 glycopeptides are not of high-mannose type.	PXD023980
Yeast	Orbitrap Fusion<br>Stepped CE: 30±10	Fission yeast (*Schizosaccharomyces pombe*).<br>Raw data contains 7 runs, with 2824 GPSMs identified, finally combined into consensus spectra of 424 precursors of 355 glycopeptides.	PXD023980

Stepped CE: HCD MS/MS is performed with multiple CEs and the data are recorded in a single spectrum for each precursor. Dual CEs: oxonium ions triggered data-dependent HCD MS/MS is performed, where data of different CEs are recorded in separate spectra; the isolation width can be different for each CE; the low and high CE spectra of the same precursor are merged in our data preprocessing procedure.

Supplementary Table 4. MS/MS datasets for testing the differentiation of glycopeptide structural isomers.

Name	Instrument	Description	Accession
Mouse1 holdout	Orbitrap Fusion<br>Stepped CE: 30±10	Holdout datasets of Mouse1 that are not used in model training.<br>Containing 6248 GPSMs that are not of high-mannose type.	PXD005411, PXD005413, PXD005412, PXD005553, PXD005555
Human1 holdout	Orbitrap Fusion Lumos<br>Dual CEs: 20 & 33	Holdout datasets of Human1 that are not used in model training.<br>Containing 15308 GPSMs that are not of high-mannose type.	PXD026649, PXD030804
Standard glyco-proteins	Orbitrap Fusion Lumos<br>Dual CEs: 20 & 33	Mixtures of fetuin, ovalbumin, human IgG, as well as RNase B (ignored since most glycosites are occupied with high-mannose glycans), before and after exoglycosidase treatments.<br>Raw data contains 14 runs with 3766 GPSMs originally identified. Among them, 2791 GPSMs that are not of high-mannose type are used.	PXD025859
Mouse CutN	Orbitrap Fusion Lumos<br>Dual CEs: 20 & 33	Mouse brain after removing terminal HexNAc by β-N-acetylglucosaminidase S.<br>Raw data contains 3 runs with 3162 GPSMs originally identified. Among them, 701 GPSMs that are not of high-mannose type are used.	PXD025859
Mouse Fut8	Orbitrap Fusion Lumos<br>Dual CEs: 20 & 33	The Mouse3 dataset.<br>Raw data contains 3 runs of wild type samples and 3 runs of knockout samples, with 11365 and 12542 GPSMs originally identified, respectively. Among them, 5973 and 4501 GPSMs that are not of high-mannose type are used.	PXD035158

Stepped CE: HCD MS/MS is performed with multiple CEs and the data are recorded in a single spectrum for each precursor. Dual CEs: oxonium ions triggered data-dependent HCD MS/MS is performed, where data of different CEs are recorded in separate spectra; the isolation width can be different for each CE; the low and high CE spectra of the same precursor are merged in our data preprocessing procedure.

Supplementary Table 5. DIA datasets.

**Name**	**Description**
Yeast	Fission yeast (*Schizosaccharomyces pombe*). Raw data contains 4 technical replicated runs.
Serum	Human serum. Raw data contains 3 technical replicated runs.
Mix	Mixture of human serum and budding yeast (*Saccharomyces cerevisiae*). The samples were mixed at definite ratios (human/yeast): (1) sample S10, 1:1; (2) sample S12, 1:1.2; (3) sample S15, 1:1.5. The final concentration ratio among these samples (S10:S12:S15) is 1:0.9:0.8 for human and 1:1.1:1.2 for yeast. Raw data contains 9 runs, with 3 technical replicated runs per sample.

All the datasets were acquired on Orbitrap Fusion with stepped CE of 30±10. Raw data are available with the accession PXD023980.

Supplementary Table 6. Experimental and predicted libraries for DIA data analysis.

Name	Description
Yeast DDALib	A sample-specific spectral library of fission yeast generated from DDA data.<br>Containing 502 precursors of 434 glycopeptides, 412 site-specific glycans, 156 protein glycosites.
Yeast PredMS2	A spectral library predicted from glycopeptides in yeast DDALib, with predicted fragment intensities and original retention time values in yeast DDALib. The model trained with Mouse1 was used for fragment intensity prediction.<br>Containing 497 precursors of 430 glycopeptides, 407 site-specific glycans, 154 protein glycosites.
Yeast PredLib	A spectral library predicted from glycopeptides in yeast DDALib, with predicted fragment intensities and retention time values. The models trained with Mouse1 was used for fragment intensity and iRT prediction. Predicted iRT values were calibrated to retention time space of the experimental LC gradient.<br>Containing 497 precursors of 475 glycopeptides, 469 site-specific glycans, 154 protein glycosites.
Serum DDALib	A sample-specific spectral library of human serum generated from DDA data.<br>Containing 3518 precursors of 2402 glycopeptides, 2082 site-specific glycans, 396 protein glycosites.
Serum PredMS2	A spectral library predicted from glycopeptides in serum DDALib, with predicted fragment intensities and original retention time values in yeast DDALib. A model trained with Mouse1 and finetuned with serum data was used for fragment intensity prediction.<br>An extended spectral library of fission yeast predicted from glycopeptide lists in our previous study. The models trained with Mouse1 was used for fragment intensity and iRT prediction. Predicted iRT values were calibrated to retention time space of the experimental LC gradient.<br>Containing 3528 precursors of 2410 glycopeptides, 2081 site-specific glycans, 395 protein glycosites.
Serum PredLib	A spectral library predicted from glycopeptides in serum DDALib, with predicted fragment intensities and retention time values. Models trained with Mouse1 and finetuned with serum data was used for fragment intensity and iRT prediction. Predicted iRT values were calibrated to retention time space of the experimental LC gradient.<br>Containing 3528 precursors of 2410 glycopeptides, 2081 site-specific glycans, 395 protein glycosites.

GproDIA applies filters to spectral libraries, leading to slightly different numbers of entries finally present in the spectral libraries from the starting glycopeptide lists. Therefore, the sizes of predicted spectral libraries do not exactly equal to the DDA-based libraries.

Supplementary Table 7. Extended libraries for DIA data analysis.

Name	Description
Serum DDAExt (5k)	A semi-empirically extended spectral library of human serum generated by GproDIA from DDALib.<br>Containing 5508 precursors of 3433 glycopeptides, 3009 site-specific glycans, 396 protein glycosites.
Serum PredExt (5k)	An extended spectral library of human serum predicted from the same starting glycopeptide list as DDAExt. Models trained with Mouse1 and finetuned with serum data was used for fragment intensity and iRT prediction. Predicted iRT values were calibrated to retention time space of the experimental LC gradient.<br>Containing 5780 precursors of 3543 glycopeptides, 3092 site-specific glycans, 395 protein glycosites.
Serum PredExt (7k)	An extended spectral library of human serum predicted from glycopeptide lists collected from previous studies. Models trained with Mouse1 and finetuned with serum data was used for fragment intensity and iRT prediction. Predicted iRT values were calibrated to retention time space of the experimental LC gradient.<br>Containing 6807 precursors of 4313 glycopeptides, 3778 site-specific glycans, 482 protein glycosites.
Serum PredExt (10k)	An extended spectral library of human serum predicted from glycopeptide lists collected from previous studies. Models trained with Mouse1 and finetuned with serum data was used for fragment intensity and iRT prediction. Predicted iRT values were calibrated to retention time space of the experimental LC gradient.<br>Containing 9443 precursors of 6532 glycopeptides, 5795 site-specific glycans, 621 protein glycosites.

Glycans in the glycopeptide precursors are identified as monosaccharide compositions. A “site-specific glycan” is referred to a glycan composition on a protein glycosite, which contains a group of glycopeptide variants resulting from missed cleavages in protein digestion.

Supplementary Table 8. Monosaccharide-based entrapment libraries.

Name	Description
Yeast DDALib Entrap	A spectral library generated by combining the fission yeast DDALib and entrapment entries from DDA-based libraries. The entrapment entries have the peptide sequences of yeast and the glycans of human.<br>Containing 502 yeast precursors from yeast DDALib and 500 entrapment precursors.
Yeast PredLib Entrap	A spectral library generated by combining the fission yeast PredLib and entrapment entries predicted from peptide sequences of yeast and glycans of human.<br>Containing 497 yeast precursors from yeast PredLib and 499 entrapment precursors.
Serum DDALib Entrap	A spectral library generated by combining the serum DDALib and entrapment entries from DDA-based libraries. The entrapment entries have the peptide sequences of human and glycans of *Arabidopsis thaliana*.<br>Containing 3518 serum precursors from yeast DDALib and 3500 entrapment precursors.
Serum PredLib Entrap	A spectral library generated by combining the serum PredLib and entrapment entries predicted from peptide sequences of human serum and fake glycans, which have similar structures with those of *A. thaliana* except that xyloses not supported by the prediction model are replaced with NeuGc.<br>Containing 3528 serum precursors from serum PredLib and 3459 entrapment precursors.
Serum PredExt (5k) Entrap	A spectral library generated by combining the serum PredExt (5k) and entrapment entries predicted from peptide sequences of human serum and NeuGc-containing fake glycans.<br>Containing 5480 serum precursors from serum PredExt (5k) and 6095 entrapment precursors.
Serum PredExt (7k) Entrap	A spectral library generated by combining the serum PredExt (7k) and entrapment entries predicted from peptide sequences of human serum and NeuGc-containing fake glycans.<br>Containing 6807 serum precursors from serum PredExt (7k) and 6840 entrapment precursors.
Serum PredExt (10k) Entrap	A spectral library generated by combining the serum PredExt (10k) and entrapment entries predicted from peptide sequences of human serum and NeuGc-containing fake glycans.<br>Containing 9443 serum precursors from serum PredExt (10k) and 9443 entrapment precursors.

GproDIA applies filters to spectral libraries, leading to slightly different numbers of entries finally present in the spectral libraries from the starting glycopeptide lists. Therefore, the sizes of predicted spectral libraries do not exactly equal to the DDA-based libraries. Neither do the numbers of entrapment entries equal to those of the sample-specific precursors.

Supplementary Table 9. Composition-based entrapment libraries.

Name	Description
Serum PredLib Entrap 2	A spectral library generated by combining the serum PredLib and entrapment entries predicted from peptide sequences of human serum and fake glycans, which were generated from human glycans by replacing all the branch Hex with HexNAc.<br>Containing 3528 serum precursors from serum PredLib and 3500 entrapment precursors.
Serum PredExt (5k) Entrap 2	A spectral library generated by combining the serum PredExt (5k) and entrapment entries predicted from peptide sequences of human serum and full-HexNAc fake glycans.<br>Containing 5480 serum precursors from serum PredExt (5k) and 6060 entrapment precursors.
Serum PredExt (7k) Entrap 2	A spectral library generated by combining the serum PredExt (7k) and entrapment entries predicted from peptide sequences of human serum and full-HexNAc fake glycans.<br>Containing 6807 serum precursors from serum PredExt (7k) and 6807 entrapment precursors.
Serum PredExt (10k) Entrap 2	A spectral library generated by combining the serum PredExt (10k) and entrapment entries predicted from peptide sequences of human serum and full-HexNAc fake glycans.<br>Containing 9443 serum precursors from serum PredExt (10k) and 9450 entrapment precursors.

GproDIA applies filters to spectral libraries, leading to slightly different numbers of entries finally present in the spectral libraries from the starting glycopeptide lists. Therefore, the sizes of predicted spectral libraries do not exactly equal to the DDA-based libraries. Neither do the numbers of entrapment entries equal to those of the sample-specific precursors.

Supplementary Table 10. Spectral libraries for quantification performance evaluation.

Name	Description
Mix DDALib	A combined spectral library of budding yeast and human serum generated from DDA data.<br>Containing 850 precursors of 667 glycopeptides, 613 site-specific glycans, 241 protein glycosites from budding yeast; 3518 precursors of 2402 glycopeptides, 2082 site-specific glycans, 396 protein glycosites from human serum.
Mix PredMS2	A spectral library predicted from glycopeptides in mix DDALib, with predicted fragment intensities and original retention time values in mix DDALib. A model trained with Mouse1 and finetuned with serum data was used for fragment intensity prediction.<br>Containing 848 precursors of 665 glycopeptides, 609 site-specific glycans, 243 protein glycosites from budding yeast; 3528 precursors of 2410 glycopeptides, 2081 site-specific glycans, 395 protein glycosites from human serum.
Mix PredLib	A spectral library predicted from glycopeptides in mix DDALib, with predicted fragment intensities and retention time values. Models trained with Mouse1 and finetuned with serum data was used for fragment intensity and iRT prediction. Predicted iRT values were calibrated to retention time space of the experimental LC gradient.<br>Containing 848 precursors of 665 glycopeptides, 609 site-specific glycans, 243 protein glycosites from budding yeast; 3528 precursors of 2410 glycopeptides, 2081 site-specific glycans, 395 protein glycosites from human serum.
Mix PredExt	An extended spectral library predicted from glycopeptides in mix DDALib and human serum glycopeptide lists collected from our previous study. Models trained with Mouse1 and finetuned with serum data was used for fragment intensity and iRT prediction. Predicted iRT values were calibrated to retention time space of the experimental LC gradient.<br>Containing 848 precursors of 665 glycopeptides, 609 site-specific glycans, 243 protein glycosites from budding yeast; 5780 precursors of 3543 glycopeptides, 3092 site-specific glycans, 395 protein glycosites from human serum.

GproDIA applies filters to spectral libraries, leading to slightly different numbers of entries finally present in the spectral libraries from the starting glycopeptide lists. Therefore, the sizes of predicted spectral libraries do not exactly equal to the DDA-based libraries.

Glycans in the glycopeptide precursors are identified as monosaccharide compositions. A “site-specific glycan” is referred to a glycan composition on a protein glycosite, which contains a group of glycopeptide variants resulting from missed cleavages in protein digestion.

Supplementary Note 1. Evaluation of prediction error of the intensity ratio between peptide and glycan fragments.

Prediction error of the intensity ratio between peptide and glycan fragments are visualized in **Supplementary Figs. 9**, **10**, **17**, **18**, **21**, and **22**. Evaluated on the same datasets used for model training, the predicted ratio was not biased on the holdout subset. For cross-dataset evaluation, the model trained with Mouse 1 underestimated the ratio on some datasets and overestimated it on others. The model trained with Human 1 underestimated the ratio (biased towards glycan ions) on most of the datasets, while models trained with Mouse 2 or Human 2 overestimated the ratio (biased towards peptide ions) on some datasets. The results also suggest the variation in glycopeptide fragmentation among these datasets.

In order to explore how this ratio affects the overall spectral similarity, we intentionally introduced an "error" by adding an offset to the predicted ratio value before merging the peptide and glycan fragments into the whole predicted spectra. The spectral similarities decreased with the absolute value of ratio offset growing (**Supplementary Figs. 11** and **12**). The spectral similarities were less susceptible to negative ratio error as glycan fragments are more intensive.

Supplementary Note 2. Evaluation of the impact of incorrectly identified spectra in the training data.

As the training data were collected from identification results of biological samples, they were not perfect ground truth and a proportion of the glycopeptides may have incorrectly assigned compositions or structures. For example, glycopeptides from the Mouse1 dataset were extensively adducted with ammonium, which was not included in the StrucGP search used to identify the glycopeptides prior to building the training data. GPSMs with excessive fucoses were likely incorrect since a NeuAc with an ammonium adduct can have a nearly identical mass with a Hex and a Fuc. Therefore, glycopeptide spectra with more than two fucoses in the Mouse 1 dataset were removed.

Even after excluding common cases of incorrect glycan assignments, there will remain some incorrectly identified glycans in the training data. To explore their impact on the resulting spectrum predictions, we created a “dirty” dataset by intentionally disturbing 5% of GPSMs in the Mouse1 datasets. These GPSMs were randomly selected from those containing Hex and Fuc, or containing NeuAc. For each GPSM, the glycan annotation was changed to another glycan with one more NeuAc, one fewer Hex and one fewer Fuc than the original glycan, or inversely. Following the same data preprocessing procedure (including consensus spectra generation), the dirty dataset was used to training a model, which was then evaluated on the original Mouse 1 dataset (**Supplementary Figs. 13** and **14**). Compared with the original model, the dirty model performed slightly worse for the prediction of glycan fragment intensities (with the median SA of 0.14, corresponding to DP of 0.97, for the holdout subset) and the whole spectrum (SA of 0.16, DP of 0.97). The prediction accuracy for the glycopeptides related to the disturbed GPSMs was affected (with SA increased from 0.13 to 0.20 for the glycan part and from 0.14 to 0.20 for the whole spectrum, corresponding to DP decreasing from 0.98 to 0.95). These results suggest the importance of quality control of training data. We hope that the community could provide more high-quality glycoproteomic datasets in the future, which would lead to better performance for spectrum prediction.

Supplementary Note 3. Performance evaluation of a model trained using a combined dataset.

We trained a model using a larger dataset combined from six datasets of mouse and human (i.e., Mouse 1, Mouse 3, Mouse 4, Human 1, Human 3, and Human 4). The combined dataset (consensus spectra) was randomly partitioned into 4/5 involved in training and 1/5 holdout. The results are shown in **Supplementary Figs. 19–22**. The model achieved high prediction accuracy for the six datasets (with the median SA values of 0.25–0.17 for the peptide part, 0.18–0.13 for the glycan part, and 0.22–0.15 for the whole spectrum, corresponding to DP of 0.93–0.96, 0.96–0.98, and 0.94–0.97, respectively, for the holdout subsets). The model was then evaluated on the remaining three datasets (i.e., Mouse 2, Human 2, and Yeast). On the Mouse 2 and Human 2 datasets, the model outperformed the models trained using the Mouse 1 or Human 1 dataset alone. The median SA was 0.27–0.22 for the peptide part, 0.21–0.19 for the glycan part, and 0.26–0.21 for the whole spectrum, corresponding to DP of 0.91–0.94, 0.94–0.96, and 0.92–0.95, respectively. On the Yeast dataset, the prediction similarities (with the median SA of 0.29 for the peptide part, 0.13 for the glycan part, and 0.19 for the whole spectrum, corresponding to DP of 0.90, 0.98, and 0.95, respectively) were close to those using the model trained by Mouse 1.

We also trained a model with B ions using the combined dataset (**Supplementary Figs. 29** and **30**). These results suggest that incorporating more datasets of different instruments or organisms for model training would lead to better generalization in the future.

Supplementary Note 4. Representative types of conflicting identifications by spectral library searching.

The conflicting results of spectral library searching with the original StrucGP annotations were counted according to the misidentified branch types (**Supplementary Figs. 33**, **35** and **37**). Representative types of conflicting identifications include:

(1) Positions of branch Hex. For 3% – 10% of the GPSMs with a HexNAc-Hex branch, the Hex was moved to the oligomannose substructure of a hybrid type glycan after rescoring. Inversely, for 3% – 20% of the cases with a HexNAc branch, a Hex was moved to the branch from the oligomannose substructure.

(2) Isomeric branches. For 4% of the GPSMs with a HexNAc-Hex-NeuGc branch, the NeuGc was moved to the HexNAc after rescoring. Inversely, for 50% of the cases with a HexNAc(-NeuGc)-Hex branch, the NeuGc was moved to the Hex. Similarly, 60% – 70% cases of HexNAc(-NeuAc)-Hex was rescored as HexNAc-Hex-NeuAc, and 2% – 4% cases of HexNAc(-Fuc)-Hex rescored as HexNAc-Hex-Fuc.

(3) Monosaccharide translocation between two branches. For 30% of the GPSMs with a HexNAc-Hex-NeuAc branch and a HexNAc(-Fuc)-Hex branch, a HexNAc(-Fuc)-Hex-NeuAc branch and a HexNAc-Hex branch were identified after rescoring. Translocation occurred inversely for 30% – 50% of the cases with a HexNAc(-Fuc)-Hex-NeuAc branch and a HexNAc-Hex branch.

(4) Bisecting HexNAc and branch HexNAc. For 8% – 20% of the GPSMs with a bisecting HexNAc, the bisecting HexNAc was rescored as a HexNAc branch. For another 3% – 6% cases, the bisecting HexNAc was rescored as a HexNAc-Hex branch (taking a Hex from the oligomannose substructure). Inversely, for 3% – 6% of the cases with a HexNAc branch, they were rescored as a bisecting HexNAc.

(5) Core Fuc and branch Fuc. For 0.5% – 8% of the GPSMs with a HexNAc(-Fuc)-Hex branch, the Fuc was rescored as a core Fuc, with a HexNAc-Hex branch remaining. For 0.1% – 10% of the GPSMs with a HexNAc(-Fuc)-Hex-Fuc branch, one Fuc was rescored as a core Fuc, with a HexNAc(-Fuc)-Hex branch remaining. Translocation occurred inversely for 2% of the cases with a HexNAc(-Fuc)-Hex branch and a core Fuc.

Supplementary Note 5. Example spectral matches where spectral library searching dismisses or confirms the terminal HexNAc in the original StrucGP annotations.

For the largest proportion of GPSMs where spectral library searching dismisses the terminal HexNAc in the original StrucGP annotations, the original terminal HexNAc moved into a HexNAc-Hex branch or a fucosylated branch (**Supplementary Fig. 38**). An example spectral match where the top candidate had a HexNAc-Hex branch is shown in **Supplementary Figs. 39** and **40**, which is explained in the main text.

For a spectral match of VLTLAN[H(6)N(4)F(3)]FTTK^{2+}, the original glycan structure annotation by StrucGP has a core fucose and a bisecting HexNAc, whereas a candidate glycan structure with no bisecting HexNAc was ranked first after rescoring by spectral library searching (**Supplementary Fig. 41**). The original glycan had been recognized as bisecting HexNAc-containing structure by StrucGP as the query spectrum contained the characteristic ion Y-H(1)N(3). However, this peak was of very low intensity (probably noise signals) in the query spectrum. The intensity pattern of Y ions in the query spectrum was slightly more similar to that of the rescored structure. The B ion H(1)N(1) was quite intense in the query spectrum, bearing a strong resemblance to that of the rescored glycan structure. While the query spectrum contained the B ion H(1)N(1)F(2) of low relative intensity, spectral library searching was not able to determine whether the two outer fucoses were in the same branch since the predicted spectra for the two cases were too similar. Anyway, either of the top two candidates did not contain a bisecting HexNAc.

For some other GPSMs, spectral library searching confirmed the original StrucGP annotations. For a spectral match of C[+57]HLN[H(5)N(4)F(2)]NSEcCMPIK^{3+}, spectral library searching reported a glycan structure with a bisecting HexNAc, the same as the original glycan structure annotation by StrucGP (**Supplementary Fig. 42**). The characteristic ions Y-H(1)N(3) and Y-H(1)N(3)F(1) were very weak in the query spectrum. However, the intensity pattern of Y ions in the query spectrum was more similar to that of the bisecting structure than other candidates. The candidate structure with no terminal HexNAc should not produce the ions Y-H(5)N(3)F(x) (x = 0, 1, 2), which were observed in the query spectrum. These ions were expected to be more

intense for the bisecting structure than the candidate structure with a branch terminal HexNAc. In addition, the intensity pattern of B ions in the query spectrum inclined to the bisecting structure as the B ion H(1)N(1) was relatively weaker.

For a spectral match of NPN[H(5)N(4)F(1)]GTVTVISR^{3+}, spectral library searching reported a glycan structure with a branch terminal HexNAc, the same as the original glycan structure annotation by StrucGP (**Supplementary Fig. 43**). The candidate structure with two HexNAc-Hex branches should result in highly intense peaks of the Y ion Y-H(4)N(3)F(1) and B ion H(1)N(1) originated from the cleavage of either branch, while they were lower in the query spectrum. The candidate structure with a bisecting HexNAc should produces the characteristic ion Y-H(1)N(3), together with its core fucosylated counterpart Y-H(1)N(3)F(1) of higher relative intensity. It was less convincing to recognize the bisecting HexNAc by observing the former solely in the query spectrum. The structure with branch terminal HexNAc was finally chosen in the light of slightly more similar intensity patterns of Y ions and B ions compared to those of the bisecting structure. The glycan fragmentation graph in the model can explain the subtle difference between the predicted spectra of the candidate structures with bisecting or branch terminal HexNAc (**Supplementary Fig. 44**). The ion Y-H(4)N(2) and its core fucosylated counterpart Y-H(4)N(2)F(1) were originated from cleavages of the HexNAc-Hex branch (VII) and the bisecting or branch terminal HexNAc (IX). The ions Y-H(3)N(2) and Y-H(3)N(2)F(1) could originated from cleavages VII and IX, together with the cleavage of a Hex (V). For the structure with branch terminal HexNAc, they could also originate from a single cleavage of a core hexose with the HexNAc-Hex branch (VI), while cleavage IX was still necessary in this context for the bisecting structure. Likewise for the ions Y-H(2)N(2) and Y-H(2)N(2)F(1). The attention weights indicated that cleavage IX was the determinative step. The lower relative intensities of these ions for the bisecting structure could probably be related to the distinct properties in MS/MS cleavages of bisecting and branch terminal HexNAc.

Supplementary Note 6. Example spectral matches where spectral library searching dismisses or confirms the core fucosylation in the original StrucGP annotations.

For the largest proportion of GPSMs where spectral library searching dismisses the core fucosylation in the original StrucGP annotations, the original core fucose moved into a HexNAc(-Fuc)-Hex branch (**Supplementary Fig. 45**).

For a spectral match of VGVHIN[H(4)N(3)F(1)]NTQTK^{3+}, the original glycan structure annotation by StrucGP has a core fucose, whereas a candidate glycan structure with a branch fucose was ranked first after rescoring by spectral library searching (**Supplementary Fig. 46**). The original glycan had been recognized as core fucosylated structure by StrucGP as the query spectrum contained the characteristic ion Y-H(2)N(2)F(1) and Y-H(3)N(2)F(1). These ions should be more intense than their Y-H(2)N(2) and Y-H(3)N(2) in the predicted spectrum. However, these ions were not relatively weak in the query spectrum and were probably "ghost" peaks caused by fucose migration in MS/MS[1]. The ions Y-N(1)F(1) and Y-N(2)F(1) were missing in the query spectrum, refuting the core fucosylated candidate. In addition, the intensity pattern of B ions in the query spectrum inclined to the branch fucosylated structure as the relative intensity of H(1)N(1)F(1) and H(1)N(1) was very close between the query and predicted spectra.

Likewise for a spectral match of N[H(5)N(4)F(2)]ASNMEYR^{3+}, spectral library searching did not support the original core fucosylated structure based on the intensity pattern of the ions Y-N(1)F(1) to Y-H(3)N(2)F(1) and their non-fucosylated counterparts (**Supplementary Fig. 47**). The candidate structure with two outer fucoses in the same branch was ranked first because of better spectral similarity than that with fucoses in separate branches. Despite lack of additional evidences to discriminate the two cases, either of the them was not core fucosylated.

For some other GPSMs, spectral library searching confirmed the original StrucGP annotations. For a spectral match of N[H(4)N(3)F(1)]ATGMEVGWYR^{2+}, the core fucose was confirmed by the intensity pattern of Y ions (**Supplementary Fig. 48**), mainly the intense characteristic ion Y-N(1)F(1), while assumptions of a branch fucose were negated with the presence of highly intense B ion H(1)N(1) and bare of

H(1)N(1)F(1).

For a spectral match of KVLVAPPSEEAN[H(6)N(5)F(3)]TTK^{3+}, at least two of the three fucoses were in the branches, with one fucose left over that was potentially linked to the core (**Supplementary Fig. 49** and **50**). The core fucose was confirmed by not only the characteristic ions Y-N(1)F(1), Y-N(2)F(1), and Y-H(2)N(2)F(1), but also the intensity pattern of Y-H(3)N(3)F(2) and Y-H(4)N(3)F(2). Y-H(3)N(3)F(2) was theoretically nonexistent for the candidate structure with fucoses in separate branches and unique to cleavage of the core Hex with the two branches (IX) for the candidate structure with two fucosylated branches. For the core fucosylated structure, it could be generated via two fragmentation pathways: (1) the cleavages of the two HexNAc-Hex branches (X and XII) and the terminal Hex-Fuc (VI); (2) the cleavages of a branch terminal fucose (either VII or VIII) and the core Hex with the two branches (IX), where the attention weights indicated that the cleavage IX was the determinative step. The higher intensity of Y-H(3)N(3)F(2) for the core fucosylated structure than the other candidates was probably resulted from the first pathway without the need to break the core. Y-H(4)N(3)F(2) was mainly originated from the cleavages of the two HexNAc-Hex branches (X and XII) and the terminal fucose (VII) for the core fucosylated structure. It could be generated by the cleavages of two branches (X and XIII) with the avoidance of cleavage VII for the candidate structure with two fucosylated branches. The attention weights of cleavage VII to other fragments were low, indicating that this cleavage step could easily happen and the terminal fucose was too fragile to keep. This explained the lower intensity of Y-H(4)N(3)F(2) for the candidate structure with two fucosylated branches than the core fucosylated structure.

Supplementary Note 7. Prediction of glycopeptide retention time.

The model architecture for glycopeptide retention time prediction was very similar to that for fragment spectrum prediction. The charge states of glycopeptides were ignored. The peptide features output by the second two LSTM layers were summed over the peptide sequence with attention weights computed by a dense layer followed by the softmax function. The glycan node features by the second tree LSTM layer were aggregated to graph-level features using a global attention pooling readout module[2]. The aggregated features were separately transformed by a parametric ReLU and a dense layer, and finally summed to output a scalar value. Mean absolute error (MAE) was used as a loss function.

We first built a model containing only the modules to process peptide sequences and predict normalized retention time (iRT[3]) values of peptides, which was trained with peptide data collected from a HeLa proteome dataset[4]. This model allowed us to predict the iRT values of non-glycosylated peptides identified from LC-MS/MS data of glycopeptides. These peptides were then used as anchors to calibrate experimental retention time values of glycopeptides to iRT space. We envision methods can be developed using artificially synthetic or endogenous conserved glycopeptides (similar to the iRT standard kit and CiRT for peptides[5]), achieving high-precision iRT calibration and prediction in the future.

When training the model for glycopeptides, parameters of corresponding modules were port form the pretrained model for peptides and the first two BiLSTM layers were frozen. We used the Adam optimizer and 16 samples per batch. The learning rate started from 0 to 0.001 in 5 warmup epochs, and was then scheduled by cosine annealing with warm restarts[6] (with an initial interval of 45 epochs and multiplied by 2 after each restart). For model finetuning, an initial of learning rate 0.0001 was used without warmup, and iteratively reduced to 10% when the metrics had stopped improving for 5 epochs.

Supplementary Note 8. DIA analysis of the fission yeast datasets using predicted spectral libraries.

The numbers of detected glycopeptides resulting from the predicted libraries (PredMS2 with predicted fragment intensities and experimental retention times, as well as PredLib with predicted fragment intensities and retention times) were compared to the experimental library (DDALib). In average of 4 technical replicate runs, 402 ± 2 precursors of 331 ± 1 site-specific glycans were detected using PredMS2 and 378 ± 4 precursors of 315 ± 3 site-specific glycans were detected using PredLib, compared to 418 ± 2 precursors of 348 ± 2 site-specific glycans when using DDALib (**Supplementary Figs. 52a** and **53a**). Accumulating the 4 replicate runs, 422 precursors of 345 site-specific glycans were detected totally using PredMS2, among which 95% (399) precursors and 95% (329) site-specific glycans were shared in all the replicates (**Supplementary Figs. 52b** and **53b**). PredLib resulted in 93% (380/408) precursors and 94% (315/336) site-specific glycans shared in all the replicates, compared to 91% (392/433) precursors and 93% (332/358) site-specific glycans when using DDALib, indicating a slightly better data completeness. Considering identifications shared in >50% replicate runs, using the predicted MS/MS spectra resulted in a loss of 4% (401 compared to 418) precursors and 9% (328 compared to 346) site-specific glycans (**Supplementary Figs. 52c** and **53c**). Replacing the retention time values further led to a loss of 5% precursors (379 compared to 401) and site-specific glycans (313 compared to 328).

The coefficients of variation (CV) values of quantification results were calculated among the replicate runs for the evaluation of quantitative precision (**Supplementary Fig. 52d** and **53d**). The median CV values were ~12% at both precursor and site-specific glycan level using PredLib, very close to those using PredMS2 and DDALib.

Supplementary Note 9. DIA analysis using predicted spectral libraries with extended coverage.

DeepGlyco enables spectral library prediction direct from a glycopeptide list and thus can break through the limitation of spectral library coverage by DDA experiments. This feature is different from the semi-empirical library extension method implemented in GproDIA. The latter uses a *k*-nearest neighbor strategy so that a target glycopeptide can be predicted only when an experimental library contains at least *k* glycopeptides with the same peptide sequence and at least *k* ones with the same glycan as the target. In contrast, DeepGlyco does not have this limitation.

To demonstrate this difference, we generated a series of predicted spectral libraries with increasing coverage, i.e., PredExt 5k (containing ~5000 glycopeptide precursors), PredExt 7k (~7000 precursors), and PredExt 10k (~10,000 glycopeptide precursors). The coverage of PredExt 5k was close to the semi-empirical library by GproDIA (DDAExt 5k). The number of detected glycopeptides by the predicted libraries grew with the increasing library coverage (**Supplementary Fig. 56** and **57**). The CV values of quantification results among the replicate runs were very close to those using DDAExt 5k.

We further evaluated the level of false positive identifications using the entrapment strategy (**Supplementary Note 10** and **Supplementary Fig. 58**). The results demonstrate that larger library coverage was adverse to error rate control, which is currently the main limitation of predicted spectral libraries.

Supplementary Note 10. DIA analysis using entrapment spectral libraries.

We adopted the entrapment strategy to approximately estimate false positive identifications for DIA analysis. Glycopeptides with glycans not present in the samples were added to the original predicted libraries. The entrapment glycopeptides were generated in two ways:

(1) Entrapments with unlikely monosaccharides. For the yeast data, entrapment entries were glycopeptides with peptide sequence from yeast and human glycans containing Fuc or NeuAc monosaccharides. For the serum data, entrapment entries in the GproDIA publication (used for experimental libraries) were glycopeptides with peptide sequences from human and xylosylated glycans from *Arabidopsis thaliana*. Since our current model do not support xylose due to lack of training data, we kept the topology of glycan structures and merely replaced xylose with NeuGc. Therefore, the entrapment entries should still be absent in the serum sample.

(2) Entrapments with common monosaccharides but nonsensical compositions. For each human glycan (with or without Fuc or NeuAc) in the glycan database used in the study, all of its branch Hex were replaced with HexNAc (while the core Hex were kept). Among the modified glycans, those with monosaccharide compositions not shared with the original human glycans were selected as entrapment glycans. These glycans (with 3 Hex and at least 8 HexNAc) are unlikely found in human. Entrapment entries were generated using glycopeptides with peptide sequences from human and the entrapment glycans, whereafter those with *m*/*z* out of DIA window range were removed. The composition-based entrapment benchmarking is stricter than the monosaccharide-based one. Notably, it cannot be used for DDA-based spectral libraries since there are not experimental spectra of these nonsensical glycopeptides.

In all the analyses, we ensured that the entrapment glycans were different from those in the original library, and kept the number of entrapment glycopeptide precursors similar to the sizes of the original library. The entrapment hits in the results (**Supplementary Data 8** and **Supplementary Fig. 58**) were considered as false positives, and we used entrapment percentage (percentage of the number of entrapment

hits to the target hits) to compare the false positive rates relatively, although it did not measure the true error rates exactly.

Using the monosaccharide-based entrapments, the entrapment percentage using PredLib was higher than that using DDALib (1.4% compared to 1.0%) for the yeast dataset, while it was close to that using DDALib (2.3% compared to 2.5%) for the serum dataset. Using PredExt 5k and 7k, the number of entrapment hits (1.6%) did not raise with the increased glycopeptide coverage. The PredExt 10k library resulted in a higher entrapment percentage (3.1%).

Using the composition-based entrapments, the entrapment percentage using PredLib was 2.2%. For the extended libraries, PredExt 5k and 7k resulted in entrapment percentages of 3.4% and 2.3%, respectively. As the library coverage continued increasing (PredExt 10k), substantially more entrapment hits occurred, while the number of detected target glycopeptides plateaued, leading to a higher entrapment percentage (4.0%). Large libraries contain a significant fraction of “false target” glycopeptides not detectable in the samples, which would not boost the number of detected analytes. Instead, it is adverse to error rate control, which is currently the main limitation of predicted spectral libraries.

Supplementary Note 11. Potential applications of DeepGlyco in users' informatic workflows.

Although StrucGP (for DDA) and GproDIA (for DIA) was used in this study, the application of DeepGlyco is not limited to them. To prepare the data of model training/finetuning, glycopeptide MS/MS spectra with confidently identified glycan structures are needed. In addition to StrucGP, pGlyco also reports plausible glycan structures in canonical form (without strict structure-specific quality control), while most of the other common software tools for glycoproteomics data analysis identify the glycans at the level of monosaccharide compositions. Users can convert results from other search engines to DeepGlyco-supported format with the glycan structure information provided manually.

The model without B ions contains 7,111,440 parameters in total (5,456,396 for the peptide part, 1,653,507 for the glycan part, and 1537 for the intensity ratio). The model with B ions contains 7,900,433 parameters in total (additional 788,993 for the glycan B ions). In this study, model training was performed on a workstation with an Intel Core i9-12900K CPU, 64 GB RAM, and a NVIDIA GeForce RTX 3090 GPU (24 GB memory). Training the model (470 epochs) without B ions using the Mouse 1 (12,818 spectra) and Human 1 (19,137 spectra) dataset took ~20 h and ~41h, respectively. Finetuning the model (20 epochs) with Mouse 2 (5942 spectra) and Human 2 (3364 spectra) took ~21 min and ~14 min, respectively. Training the model (470 epochs) with B ions with Mouse 1 (6202 spectra) and Human 1 (15,042 spectra) took ~14 h and ~33 h, respectively. Predicting ~10,000 glycopeptide spectra took ~5 min. Users can evaluate the computational requirements for integrating DeepGlyco in their informatics workflows.

Supplementary References

1. Campos, D. et al. “Ghost” fragment ions in structure and site-specific glycoproteomics analysis. *Analytical Chemistry* **95**, 10145-10148 (2023).
2. Li, Y., Tarlow, D., Brockschmidt, M. & Zemel, R. Gated graph sequence neural networks. Preprint at https://arxiv.org/abs/1511.05493 (2015).
3. Bruderer, R., Bernhardt, O. M., Gandhi, T. & Reiter, L. High-precision iRT prediction in the targeted analysis of data-independent acquisition and its impact on identification and quantitation. *Proteomics* **16**, 2246-2256 (2016).
4. Bekker-Jensen, D. B. et al. An optimized shotgun strategy for the rapid generation of comprehensive human proteomes. *Cell Systems* **4**, 587-599.e584 (2017).
5. Parker, S. J. et al. Identification of a set of conserved eukaryotic internal retention time standards for data-independent acquisition mass spectrometry. *Molecular & Cellular Proteomics* **14**, 2800-2813 (2015).
6. Loshchilov, I. & Hutter, F. SGDR: Stochastic gradient descent with warm restarts. Preprint at https://arxiv.org/abs/1608.03983 (2016).
