# Supplementary material for: Prediction of glycopeptide fragment mass spectra by deep learning
Source: Nat Commun. 2024 Mar 19;15:2448. doi: 10.1038/s41467-024-46771-1 (PMC10951270; doi:10.1038/s41467-024-46771-1)
Supplement: Supplementary file 3 — Description of Additional Supplementary Files [file 41467_2024_46771_MOESM3_ESM.pdf]

Description of Additional Supplementary Files:

Supplementary Data 1: Example spectral matches between predicted and experimental fragment intensities.

Supplementary Data 2: Results of the holdout set of Mouse1 by searching with predicted spectral libraries.

Supplementary Data 3: Results of the holdout set of Human1 by searching with predicted spectral libraries.

Supplementary Data 4: Results of the standard glycoprotein dataset by searching with predicted spectral libraries.

Supplementary Data 5: Results of the exoglycosidase treated mouse brain dataset by searching with predicted spectral libraries.

Supplementary Data 6: Results of the dataset of wild type and Fut8 knockout mouse brain by searching with predicted spectral libraries.

Supplementary Data 7: DIA results of the fission yeast dataset using predicted spectral libraries.

Supplementary Data 8: DIA results of the human serum dataset using predicted spectral libraries.

Supplementary Data 9: DIA results using the entrapment libraries.

Supplementary Data 10: DIA results of the mixed-organism dataset using predicted spectral libraries.
