# Supplementary material for: Prediction of glycopeptide fragment mass spectra by deep learning
Source: Nat Commun. 2024 Mar 19;15:2448. doi: 10.1038/s41467-024-46771-1 (PMC10951270; doi:10.1038/s41467-024-46771-1)
Supplement: Supplementary file 4 — Supplementary Data 1 [file 41467_2024_46771_MOESM4_ESM.pdf]

Prediction of glycopeptide fragment mass spectra by deep learning

Yi Yang et al.

Supplementary Data 1. Example spectral matches between predicted and experimental fragment intensities.

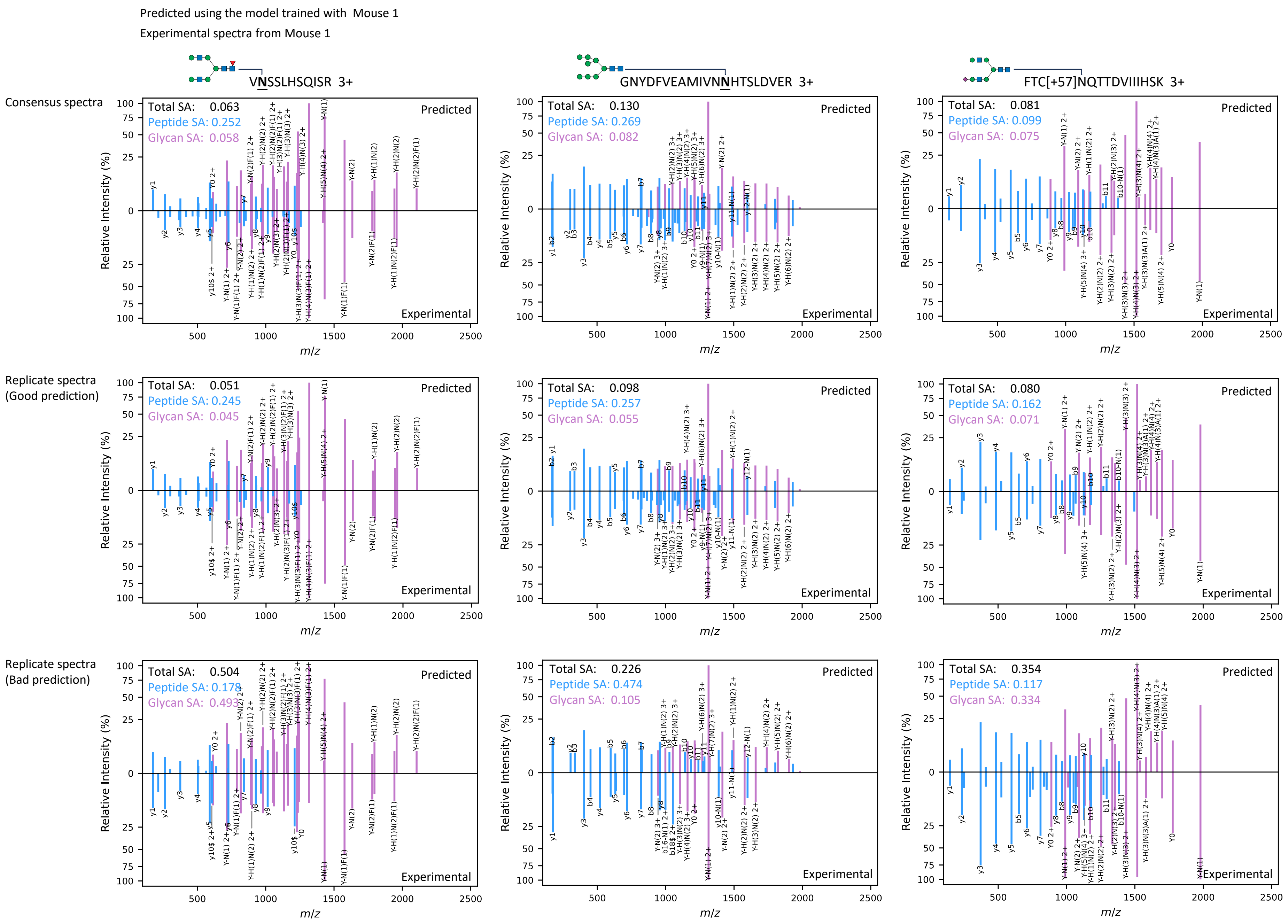

Predicted using the model trained with Mouse 1
Experimental spectra from Mouse 1
VNSSLHSQISR 3+
GNYDFVEAMIVNNHTSLDVER 3+
FTC[+57]NQTTDVIIIHSK 3+
Consensus spectra
Replicate spectra (Good prediction)
Replicate spectra (Bad prediction)
Total SA: 0.063
Peptide SA: 0.252
Glycan SA: 0.058
Total SA: 0.130
Peptide SA: 0.269
Glycan SA: 0.082
Total SA: 0.081
Peptide SA: 0.099
Glycan SA: 0.075
Total SA: 0.051
Peptide SA: 0.245
Glycan SA: 0.045
Total SA: 0.098
Peptide SA: 0.257
Glycan SA: 0.055
Total SA: 0.080
Peptide SA: 0.162
Glycan SA: 0.071
Total SA: 0.504
Peptide SA: 0.178
Glycan SA: 0.493
Total SA: 0.226
Peptide SA: 0.474
Glycan SA: 0.105
Total SA: 0.354
Peptide SA: 0.117
Glycan SA: 0.334
Predicted
Experimental
Relative Intensity (%)
m/z

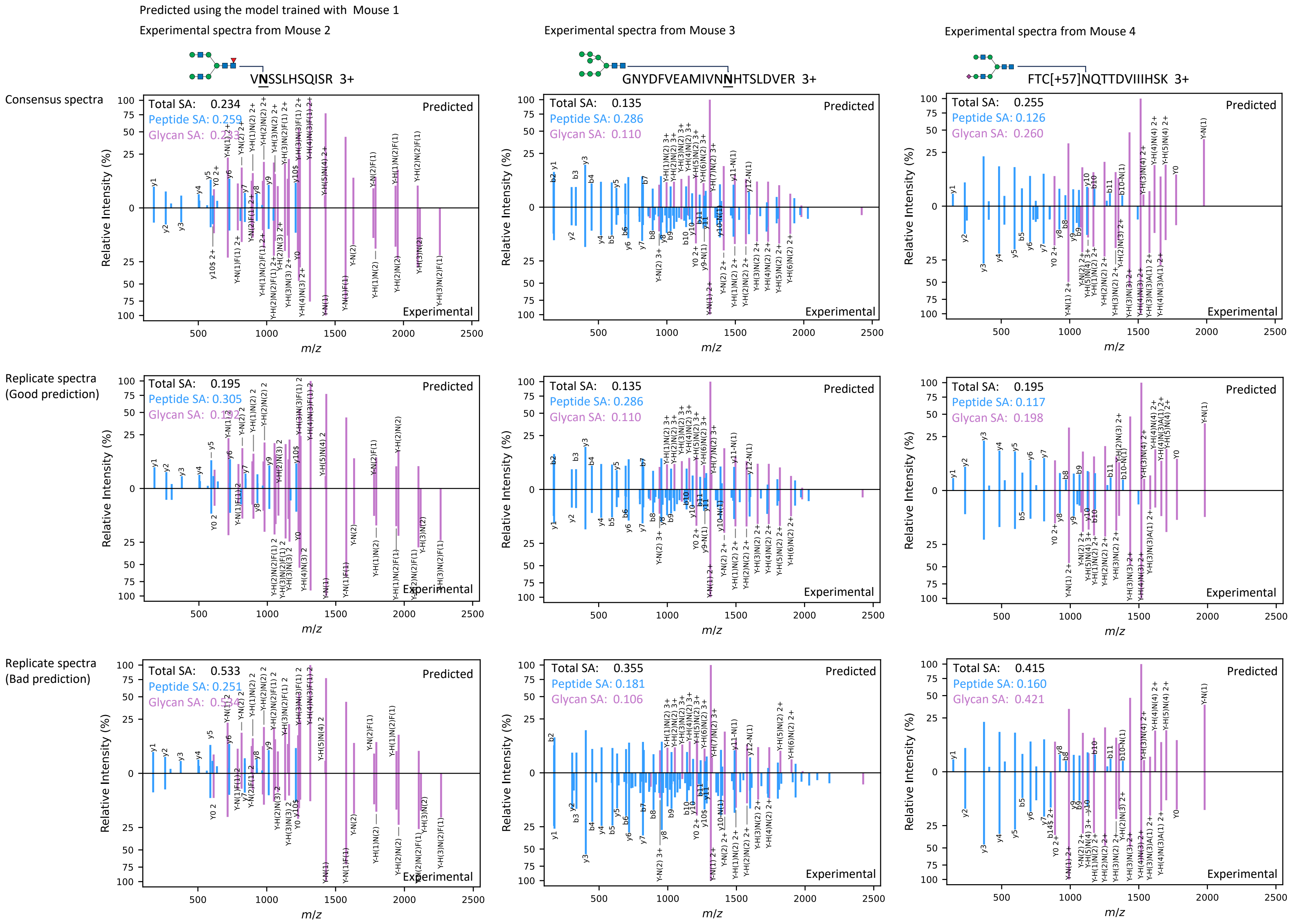

Predicted using the model trained with Mouse 1
Experimental spectra from Mouse 2
Experimental spectra from Mouse 3
Experimental spectra from Mouse 4
VNSSLHSQISR 3+
GNYDFVEAMIVNNHTSLDVER 3+
FTC[+57]NQTTDVIIIHSK 3+
Consensus spectra
Replicate spectra (Good prediction)
Replicate spectra (Bad prediction)
Total SA: 0.234
Peptide SA: 0.259
Total SA: 0.135
Peptide SA: 0.286
Glycan SA: 0.110
Total SA: 0.255
Peptide SA: 0.126
Glycan SA: 0.260
Total SA: 0.195
Peptide SA: 0.305
Total SA: 0.135
Peptide SA: 0.286
Glycan SA: 0.110
Total SA: 0.195
Peptide SA: 0.117
Glycan SA: 0.198
Total SA: 0.533
Peptide SA: 0.251
Total SA: 0.355
Peptide SA: 0.181
Glycan SA: 0.106
Total SA: 0.415
Peptide SA: 0.160
Glycan SA: 0.421
Predicted
Experimental
Relative Intensity (%)
m/z

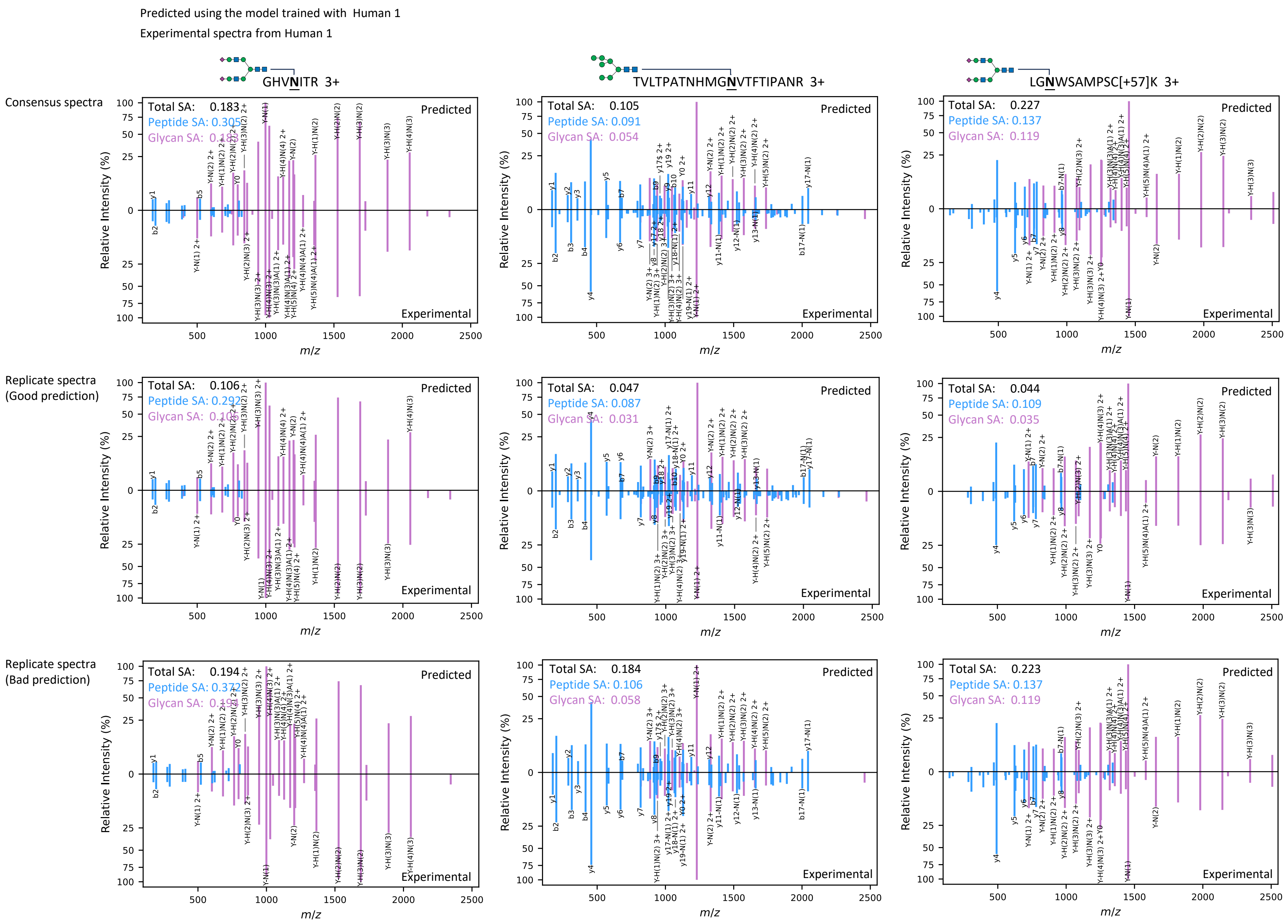

Predicted using the model trained with Human 1
Experimental spectra from Human 1
GHVNITR 3+
TVLTPATNHMGNVTFTIPANR 3+
LGNWSAMPSC[+57]K 3+
Consensus spectra
Replicate spectra (Good prediction)
Replicate spectra (Bad prediction)
Predicted
Experimental
Relative Intensity (%)
m/z
Total SA: 0.183
Peptide SA: 0.305
Glycan SA: 0.182
Total SA: 0.105
Peptide SA: 0.091
Glycan SA: 0.054
Total SA: 0.227
Peptide SA: 0.137
Glycan SA: 0.119
Total SA: 0.106
Peptide SA: 0.292
Glycan SA: 0.102
Total SA: 0.047
Peptide SA: 0.087
Glycan SA: 0.031
Total SA: 0.044
Peptide SA: 0.109
Glycan SA: 0.035
Total SA: 0.194
Peptide SA: 0.372
Glycan SA: 0.192
Total SA: 0.184
Peptide SA: 0.106
Glycan SA: 0.058
Total SA: 0.223
Peptide SA: 0.137
Glycan SA: 0.119

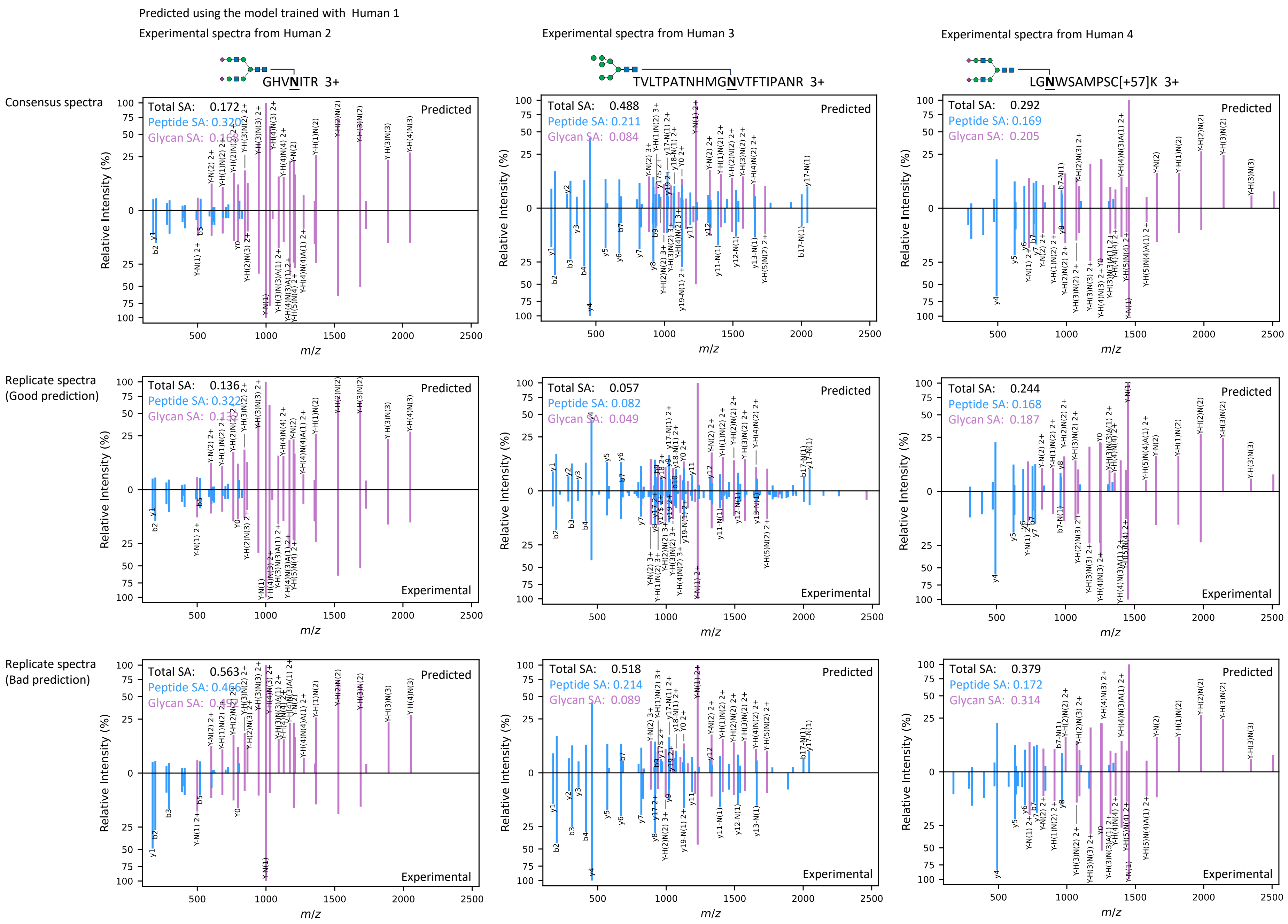
Predicted using the model trained with Human 1
Experimental spectra from Human 2
Experimental spectra from Human 3
Experimental spectra from Human 4
GHVNITR 3+
TVLTPATNHMGNVTFTIPANR 3+
LGNWSAMPSC[+57]K 3+
Consensus spectra
Replicate spectra (Good prediction)
Replicate spectra (Bad prediction)
Total SA: 0.172
Peptide SA: 0.320
Total SA: 0.488
Peptide SA: 0.211
Glycan SA: 0.084
Total SA: 0.292
Peptide SA: 0.169
Glycan SA: 0.205
Total SA: 0.136
Peptide SA: 0.322
Total SA: 0.057
Peptide SA: 0.082
Glycan SA: 0.049
Total SA: 0.244
Peptide SA: 0.168
Glycan SA: 0.187
Total SA: 0.563
Total SA: 0.518
Peptide SA: 0.214
Glycan SA: 0.089
Total SA: 0.379
Peptide SA: 0.172
Glycan SA: 0.314
Predicted
Experimental
Relative Intensity (%)
m/z
